# Characterizing Long COVID Symptoms During Early Childhood

**DOI:** 10.1001/jamapediatrics.2025.1066

**Published:** 2025-05-27

**Authors:** Rachel S. Gross, Tanayott Thaweethai, Amy L. Salisbury, Lawrence C. Kleinman, Sindhu Mohandas, Kyung E. Rhee, Jessica N. Snowden, Kelan G. Tantisira, David Warburton, John C. Wood, Patricia A. Kinser, Joshua D. Milner, Erika B. Rosenzweig, Katherine Irby, Valerie J. Flaherman, Elizabeth W. Karlson, Lori B. Chibnik, Deepti B. Pant, Aparna Krishnamoorthy, Richard Gallagher, Michelle F. Lamendola-Essel, Denise C. Hasson, Stuart D. Katz, Shonna Yin, Benard P. Dreyer, Frank Blancero, Megan Carmilani, K. Coombs, Megan L. Fitzgerald, Rebecca J. Letts, Aimee K. Peddie, Andrea S. Foulkes, Melissa S. Stockwell, and the RECOVER-Pediatrics Group Authors, Judy L. Aschner, Andrew M. Atz, Dithi Banerjee, Amanda Bogie, Hulya Bukulmez, Katharine Clouser, Lesley A. Cottrell, Kelly Cowan, Viren A. D’Sa, Allen Dozor, Amy J. Elliott, E. Vincent S. Faustino, Alexander G. Fiks, Sunanda Gaur, Maria L. Gennaro, Stewart Gordon, Uzma N. Hasan, Christina M. Hester, Alexander Hogan, Daniel S. Hsia, David C. Kaelber, Jessica S. Kosut, Sankaran Krishnan, Russell J. McCulloh, Ian C. Michelow, Sheila M. Nolan, Carlos R. Oliveira, Lynn M. Olson, Wilson D. Pace, Paul Palumbo, Hengameh Raissy, Andy Reyes, Judith L. Ross, Juan C. Salazar, Rangaraj Selvarangan, Cheryl R. Stein, Michelle D. Stevenson, Ronald J. Teufel, Alan Werzberger, John M. Westfall, Kathleen Zani, William T. Zempsky, Emily Zimmerman, Marie-Abele C. Bind, James Chan, Zoe Guan, Richard E. Morse, Harrison T. Reeder, Torri D. Metz, Jane W. Newburger, Dongngan T. Truong

**Affiliations:** 1 Department of Pediatrics, Division of General Pediatrics, NYU Grossman School of Medicine, New York, New York; 2 Massachusetts General Hospital Biostatistics, Boston; 3 Department of Medicine, Harvard Medical School, Boston, Massachusetts; 4 School of Nursing, Virginia Commonwealth University, Richmond; 5 Department of Pediatrics, Division of Population Health, Quality, and Implementation Sciences (PopQuIS), Rutgers Robert Wood Johnson Medical School, New Brunswick, New Jersey; 6 Bristol Myers Squibb Children’s Hospital, New Brunswick, New Jersey; 7 Department of Pediatrics, Division of Infectious Diseases, Children’s Hospital Los Angeles, Keck School of Medicine, University of Southern California, Los Angeles; 8 Department of Pediatrics, Division of Child and Community Health, UC San Diego School of Medicine, Rady Children’s Hospital, San Diego, California; 9 Department of Pediatrics, Division of Infectious Diseases, University of Tennessee Health Sciences Center, Memphis; 10 Department of Pediatrics, Division of Respiratory Medicine, UC San Diego School of Medicine, Rady Children’s Hospital, San Diego, California; 11 Department of Pediatrics, Division of Neonatal-Perinatal Medicine, Children’s Hospital Los Angeles, Keck School of Medicine, University of Southern California, Los Angeles; 12 Department of Pediatrics, Division of Cardiology, Children’s Hospital Los Angeles, Keck School of Medicine, University of Southern California, Los Angeles; 13 Department of Pediatrics, Division of Pediatric Allergy, Immunology and Rheumatology, Columbia University Vagelos College of Physicians and Surgeons, New York, New York; 14 Department of Pediatrics, Division of Cardiology, Columbia University Vagelos College of Physicians and Surgeons, New York, New York; 15 Department of Pediatrics, Division of Critical Care, University of Arkansas for Medical Sciences, Little Rock; 16 Department of Pediatrics, Division of General Pediatrics, University of California, San Francisco; 17 Division of Rheumatology, Inflammation, and Immunity, Department of Medicine, Brigham and Women’s Hospital, Boston, Massachusetts; 18 Mass General Brigham, Boston; 19 Division of Neurology, Department of Neurology, Harvard T.H. Chan School of Public Health, Boston, Massachusetts; 20 Department of Child and Adolescent Psychiatry, Division of Child Study Center, NYU Grossman School of Medicine, New York, New York; 21 Department of Medicine, NYU Grossman School of Medicine, New York, New York; 22 Department of Pediatrics, Division of Pediatric Critical Care Medicine, NYU Grossman School of Medicine, New York, New York; 23 Division of Cardiology, Department of Medicine, NYU Grossman School of Medicine, New York, New York; 24 Department of Population Health, NYU Grossman School of Medicine, New York, New York; 25 Department of Pediatrics, Division of Developmental and Behavioral Pediatrics, NYU Grossman School of Medicine, New York, New York; 26 RECOVER Patient, Caregiver, or Community Advocate Representative, New York, New York; 27 Long Covid Families, Charlotte, North Carolina; 28 Division of Long COVID, Department of Pandemic Equity, Vermont Center for Independent Living, Montpelier; 29 Patient Led Research Collaborative, Washington, DC; 30 Department of Biostatistics, Harvard T.H. Chan School of Public Health, Boston, Massachusetts; 31 Department of Pediatrics, Division of Child and Adolescent Health, Columbia University Vagelos College of Physicians and Surgeons, New York, New York; 32 Department of Population and Family Health, Mailman School of Public Health; New York-Presbyterian Hospital, New York; 33 Center for Discovery and Innovation, Department of Pediatrics, Hackensack Meridian School of Medicine, Nutley, New Jersey; 34 Medical University of South Carolina, Charleston; 35 Children’s Mercy Hospital and Clinics, Kansas City, Missouri; 36 Department of Pediatrics, Oklahoma University Health Science Center, Oklahoma City; 37 MetroHealth System, Cleveland, Ohio; 38 Department of Pediatrics, Division of Pediatric Hospital Medicine, Hackensack Meridian School of Medicine, Nutley, New Jersey; 39 Department of Pediatrics, West Virginia University, Morgantown; 40 Department of Pediatrics, Division of Pediatric Pulmonology, University of Vermont, Burlington; 41 Department of Pediatrics, Rhode Island Hospital, Providence; 42 Department of Pediatrics, Division of Pediatric Pulmonology, Allergy and Sleep Medicine, Boston Children’s Health Physicians, New York Medical College, Valhalla; 43 Avera Research Institute, Sioux Falls, South Dakota; 44 Department of Pediatrics, Division of Critical Care, Yale University School of Medicine, New Haven, Connecticut; 45 Department of Pediatrics, Division of General Pediatrics, Children’s Hospital of Philadelphia, Perelman School of Medicine at the University of Pennsylvania, Philadelphia; 46 Department of Pediatrics, Division of Allergy, Immunology, and Infectious Diseases, Rutgers Robert Wood Johnson Medical School, New Brunswick, New Jersey; 47 Immunology, Microbiology, Infectious Diseases, Department of Public Health, Medicine, Rutgers New Jersey Medical School, Newark; 48 Pennington Biomedical Research Center, Baton Rouge, Louisiana; 49 Department of Pediatrics, Division of Infectious Diseases, Cooperman Barnabas Medical Center, Livingston, New Jersey; 50 DARTNet Institute, Aurora, Colorado; 51 Department of Pediatrics, Connecticut Children’s, University of Connecticut School of Medicine, Hartford; 52 Department of Clinical Trials Unit, Pennington Biomedical Research Center, Baton Rouge, Louisiana; 53 Departments of Pediatrics, Internal Medicine, and Population and Quantitative Health Sciences, MetroHealth System, Cleveland, Ohio; 54 Department of Pediatrics, Division of Pediatric Hospitalist, University of Hawaii, John A Burns School of Medicine, Honolulu; 55 Department of Pediatrics, Division of Pediatric Hospital Medicine, University of Nebraska Medical Center, Omaha; 56 Department of Pediatrics, Division of Infectious Diseases, Connecticut Children’s, University of Connecticut School of Medicine, Hartford; 57 Department of Pediatrics, Division of Infectious Diseases, Boston Children’s Health Physicians, New York Medical College, Valhalla; 58 Department of Pediatrics, Division of Infectious Diseases, Yale University School of Medicine, New Haven, Connecticut; 59 American Academy of Pediatrics, Itasca, Illinois; 60 Department of Pediatrics and Medicine, Division of Infectious Disease and International Health, Dartmouth-Hitchcock Medical Center, Lebanon, New Hampshire; 61 Department of Pediatrics, Division of Pulmonary, University of New Mexico School of Medicine, Albuquerque; 62 Department of Pediatrics, Nemours Children’s Health, Wilmington, Delaware; 63 Department of Pediatrics, Children’s Mercy Hospital and Clinics, Kansas City, Missouri; 64 Department of Child and Adolescent Psychiatry, NYU Grossman School of Medicine, New York, New York; 65 Department of Pediatrics, Division of Norton Children’s Emergency Medicine, University of Louisville School of Medicine, Louisville, Kentucky; 66 Department of Pediatrics, Medical University of South Carolina, Charleston; 67 Department of Pediatrics, Division of Child and Adolescent Health, Columbia University Vagelos College of Physicians and Surgeons, Best Healthcare, Monroe, New York; 68 University of Arkansas for Medical Sciences, Little Rock; 69 Department of Communication Sciences & Disorders, Northeastern University, Boston, Massachusetts; 70 Massachusetts General Hospital Biostatistics, Boston; 71 Department of Medicine, Harvard Medical School, Boston, Massachusetts; 72 Department of Department of Obstetrics and Gynecology, Division of Maternal-Fetal Medicine, University of Utah Health, Salt Lake City; 73 Harvard Medical School, Boston, Massachusetts; 74 Children’s Healthcare of Atlanta Cardiology, Emory University School of Medicine, Atlanta, Georgia

## Abstract

**Importance:**

Recent studies have identified characteristic symptom patterns of long COVID (LC) in adults and children older than 5 years. However, LC remains poorly characterized in early childhood. This knowledge gap limits efforts to identify, care for, and prevent LC in this vulnerable population.

**Objectives:**

To identify symptoms that had the greatest difference in frequency comparing children with a history of SARS-CoV-2 infection to those without, to identify differences in the types of symptoms by age group (infants/toddlers [0-2 years] vs preschool-aged children [3-5 years]), and to derive an index that can be used in research studies to identify young children with LC.

**Design, Setting, and Participants:**

This was a multisite longitudinal cohort study with enrollment from over 30 US health care and community settings, including infants, toddlers, and preschool-aged children with and without SARS-CoV-2 infection history. Study data were analyzed from May to December 2024.

**Exposure:**

SARS-CoV-2 infection.

**Main Outcomes and Measures:**

LC and 41 symptoms among infants/toddlers and 75 symptoms among preschool-aged children.

**Results:**

The study included 472 infants/toddlers (mean [SD] age, 12 [9] months; 278 infected with SARS-CoV-2; 194 uninfected; 234 male [50%]; 73 Black or African American [16%]; 198 Hispanic, Latino, or Spanish [43%]; 242 White [52%]) and 539 preschool-aged children (mean [SD] age, 48 [10] months; 399 infected with SARS-CoV-2; 140 uninfected; 277 female [51%]; 70 Black or African American [13%]; 210 Hispanic, Latino, or Spanish [39%]; 287 White [54%]). The median (IQR) time between first infections and completion of symptom surveys was 318 (198-494) days for infants/toddlers and 520 (330-844) days for preschool-aged children. A research index was derived for each age group based on symptoms most associated with infection history. The index is calculated by summing scores assigned to each prolonged symptom that was present, where higher scores indicate greater magnitude of association with history of SARS-CoV-2 infection: poor appetite (5 points), trouble sleeping (3.5 points), wet cough (3.5 points), dry cough (3 points), and stuffy nose (0.5 points) for infants/toddlers, and daytime tiredness/sleepiness/low energy (6.5 points) and dry cough (3 points) for preschool-aged children. Among infants/toddlers with infection, 40 of 278 (14%) were classified as having probable LC by having an index of at least 4 points. Among preschool-aged children, 61 of 399 (15%) were classified as having probable LC by having an index of at least 3 points. Participants with higher indices often had poorer overall health, lower quality of life, and perceived delays in developmental milestones.

**Conclusions and Relevance:**

This cohort study identified symptom patterns and derived research indices that were distinct between the 2 age groups and differed from those previously identified in older ages, demonstrating the need to characterize LC separately across age ranges.

## Key Points

**Question:**

Which prolonged symptoms in early childhood are associated with SARS-CoV-2 infection?

**Findings:**

In the Researching COVID to Enhance Recovery (RECOVER)–Pediatrics cohort study including 472 infants/toddlers and 539 preschool-aged children, prolonged symptoms were identified that were more common in young children with infection history than those without. Infants/toddlers (0-2 years) with infection history were more likely to experience trouble sleeping, fussiness, poor appetite, stuffy nose, and cough, and preschool-aged children (3-5 years) were more likely to experience dry cough and daytime tiredness/sleepiness or low energy; empirically derived indices for long COVID research were developed from these symptoms.

**Meaning:**

Results of this cohort study suggest that symptom patterns were distinguishable across infants/toddlers and preschool-aged children, and from previously studied older children and adults.

## Teaser

This cohort study evaluates which prolonged symptoms in early childhood are associated with SARS-CoV-2 infection.

## Introduction

Long COVID (LC), or postacute sequelae of SARS-CoV-2 infection, is a chronic condition with diverse signs and symptoms that emerge, persist, or recur following SARS-CoV-2 infection.^1,2,3^ These sequelae may last months to years after acute infection and may lead to significant impairment. Pediatric LC research has identified a diverse array of symptoms and associated conditions.^4,5,6,7,8,9,10,11,12,13,14,15,16,17,18,19,20^ A recent study^21^ characterizing LC symptoms in school-aged children and adolescents found that patterns were similar but distinct between the 2 age ranges, as well as from adults. However, few studies have focused on early childhood (birth through 5 years), leaving major gaps in pediatric LC research given previously documented age-specific differences.^22^

Studying LC in early childhood presents unique challenges due to rapid developmental changes and limited verbal communication, requiring caregivers to observe, identify, and interpret symptoms.^23^ These challenges have limited the understanding of symptom profiles in young children. Most LC studies that involve young children, including controlled studies, either do not report age-specific subgroup analyses, or young children are not well represented.^24,25^ One household cohort study^10^ reported that children younger than 2 years had lower odds of persistent symptoms than older children.

Other COVID-19 studies have focused on SARS-CoV-2 exposure during pregnancy and offspring outcomes, with mixed findings related to birth defects, prematurity, and delayed developmental milestones.^26,27^ However, incomplete knowledge of prolonged symptoms experienced after a young child’s own SARS-CoV-2 infection hinders prevention and treatment of LC in infants, toddlers, and preschool-aged children. This is a substantial gap, given that early childhood is a critical period in setting lifelong health trajectories.^28^

Since the COVID-19 pandemic onset, there has been variability in how to define LC, with the National Academies of Sciences, Engineering, and Medicine (NASEM)^3^ and the World Health Organization (WHO)^1^ defining LC as a condition lasting at least 3 months after infection with symptoms across 1 or more body systems. Although broadly inclusive, these definitions are not sufficiently specific for research and were not designed to address the risk of false positives. A robust method for determining LC status among children with a COVID-19 history that can be applied in research settings is needed to distinguish young children with LC from those without LC, to follow them up longitudinally, and to determine underlying mechanisms and long-term outcomes.

The National Institutes of Health–funded Researching COVID to Enhance Recovery (RECOVER) initiative aimed to address these gaps with a comprehensive exploration of LC across the lifespan.^29^ The objectives of this study from RECOVER-Pediatrics^30^ were to (1) identify symptoms with the greatest difference in frequency comparing children with a history of SARS-CoV-2 with children without a history of SARS-CoV-2, (2) identify differences in the types of symptoms by age group (infants/toddlers [0-2 years] vs preschool age [3-5 years]), and (3) derive indices that can be used in research studies to identify LC in early childhood. These indices have been defined previously by the RECOVER initiative for school-aged children, adolescents, and adults.^21,31,32^

## Methods

### Study Design

RECOVER-Pediatrics is a large multisite retrospective and prospective cohort study, comparing children with and without a history of a prior SARS-CoV-2 infection.^29,30^ Data in this cross-sectional analysis are from the baseline assessment of young children with and without SARS-CoV-2 infection history, enrolled from community or clinical locations (approximately 30 sites across 20 states in the US, including Puerto Rico) (eTable 1 in Supplement 1). The study was approved by the NYU Grossman School of Medicine single institutional review board, and informed consents were obtained from primary caregivers. This study followed the Strengthening the Reporting of Observational Studies in Epidemiology (STROBE) reporting guidelines.^33^

### RECOVER-Pediatrics Sample

#### Inclusion Criteria for This Analysis

Young children (0-5 years) with or without a reported history of SARS-CoV-2 infection were included. Caregivers completed a symptom survey at enrollment. Infected participants were those with a reported history of SARS-CoV-2 and whose symptom survey was completed at least 90 days after the child’s first infection (eMethods in Supplement 1). Uninfected participants were those whose history did not include a report of SARS-CoV-2 infection at the time of symptom survey completion. Participants were enrolled between March 16, 2022, and July 22, 2024, and age was defined at the time of symptom survey. Nucleocapsid SARS-CoV-2 antibodies were not assessed to confirm infection status because blood was not collected in children younger than 2 years, and many caregivers of 2- to 5-year-old children opted not to provide blood specimens.

#### Exclusion Criteria for This Analysis

Participants were excluded from the infected cohort if the first infection date was unknown or if they reported a history of multisystem inflammatory syndrome in children. Participants whose symptom survey was completed less than 90 days after the child’s SARS-CoV-2 infection were excluded to improve alignment with both WHO and NASEM definitions.^3,34,35^ Participants with less than 50% of their symptom survey complete were excluded.

### Outcomes

The symptom survey was completed at enrollment remotely or with interviewer assistance and assessed 41 symptoms among infants/toddlers and 75 among preschool-aged children across 8 domains (general, eyes/ears/nose/throat, heart/lungs, gastrointestinal, dermatologic, musculoskeletal, neurologic, behavioral/psychological) using plain language descriptions (eTable 2 in Supplement 1).^30,36^ The 34 symptoms not asked for infants/toddlers were due to the inherent difficulty assessing them during this variable and typically preverbal developmental period. Similar symptoms were combined into composite symptoms (eMethods and eTable 3 in Supplement 1).

Prolonged symptoms, the primary outcomes of the analysis, were defined as those that lasted longer than 4 weeks, started or became worse since the beginning of the COVID-19 pandemic, and were occurring when the survey was administered.

The surveys assessed Patient-Reported Outcomes Measurement Information System (PROMIS) Early Childhood Parent Report Global Health Scales, which measured caregiver description of the young child’s overall health, quality of life, physical health, and developmental milestones.^37,38^

### Covariates

History of SARS-CoV-2 infection was the exposure. Additional covariates measured included race and ethnicity (caregiver-identified groups included American Indian or Alaska Native; Asian; Black or African American; Hispanic, Latino, or Spanish; Native Hawaiian or Other Pacific Islander; White; or none of these fully describe me; respondents were able to choose all categories that applied to them), sex, geographic origin, time since SARS-CoV-2 infection, calendar time of enrollment, SARS-CoV-2 vaccination status, caregiver relationship to the child, and caregiver education (eMethods in Supplement 1). Race and ethnicity were recorded to better characterize the sample.

### Statistical Analysis

Statistical analyses were based on those used to previously develop research indices in RECOVER-Adult (18 years and older) and RECOVER-Pediatrics (school-aged children [6-11 years]; adolescents [12-17 years]) cohorts.^21,31,32^ Infants/toddlers (0-2 years) and preschool-aged children (3-5 years) were analyzed separately. First, we described the proportion of children with each individual prolonged symptom and with at least 1 prolonged symptom, stratified by infection status. We used logistic regression to evaluate the association between having each prolonged symptom and being infected, adjusting for sex and race and ethnicity (eMethods in Supplement 1). The analysis was repeated using linear regression and robust SEs to estimate risk differences. Prolonged symptoms that were present in at least 5% of infected young children were included (termed *candidates*). Second, we developed research indices based on these candidates.^39^ We used a penalized logistic regression using least absolute shrinkage and selection operator (LASSO) to select a reduced set of candidates that were most associated with infection.^40^ Because participants with a history of infection are a mixture of those who did and did not develop LC, symptoms associated with LC are more common among those with infection compared with those without infection, whereas symptoms not associated with LC should be indistinguishable between those with and without infection. The estimated regression coefficients were used to assign a score to each selected symptom, with symptoms most strongly associated with infection assigned higher scores. Each child’s overall index is calculated by adding up individual scores for each of the included symptoms noted. Among infected children, a higher index is associated with higher likelihood of having LC. This index has been shown to have high discriminatory power for identifying LC.^39^ Then, we identified an optimally defined threshold balancing sensitivity and specificity, and participant status was classified as* LC probable* if their index met the threshold or *LC unspecified* if it was below (eMethods in Supplement 1). We reported classification rates stratified by infection status and timing (on or after the Omicron variant became the dominant US strain, specified as December 1, 2021).

Third, we calculated the distribution of caregiver-reported PROMIS measures in categories based on the presence of at least 1 prolonged symptom and the research index. We also described the proportion of young children experiencing each symptom, stratified by infection and LC status.

We performed a sensitivity analysis that restricted the uninfected preschool-aged cohort to those with blood samples that confirmed the absence of nucleocapsid SARS-CoV-2 antibodies. We applied the index to this group and calculated the proportion who had LC-probable status.

To evaluate any association of time with prolonged symptoms within each age group, we fit a logistic regression model for each symptom that contributed to the index with the following predictors: child age and the amount of time between the child’s first infection and survey completion, where the latter was adjusted for using restricted cubic splines. We generated curves for the estimated probability of a given symptom over time, standardized to the overall age distribution in each age group. Meaningful deviations from a flat line suggest time-associated trends. Bootstrapping was used to estimate 95% CIs (eMethods in Supplement 1). Study data were analyzed from May to December 2024 using R software, version 4.4.2 (R Development Core Team).^41^

## Results

The study included 472 infants/toddlers (mean [SD] age, 12 [9] months; 278 infected with SARS-CoV-2; 194 uninfected; 237 female [50%]; 234 male [50%]; 1 intersex [0%]; 73 Black or African American [16%]; 198 Hispanic, Latino, or Spanish [43%]; 242 White [52%]) and 539 preschool-aged children (mean [SD] age, 48 [10] months; 399 infected with SARS-CoV-2; 140 uninfected; 277 female [51%]; 262 male [49%]; 70 Black or African American [13%]; 210 Hispanic, Latino, or Spanish [39%]; 287 White [54%]) (eFigure 1 in Supplement 1). Demographic and infection history characteristics are reported in Table 1 and eTable 4 in Supplement 1, respectively. The median (IQR) time between first infection and survey completion was 318 (198-494) days for infants/toddlers and 520 (330-844) days for preschool-aged children.

**Table 1.** Demographic Characteristics of the Cohort, Stratified by Age Group and SARS-CoV-2 Infection Status

Participant characteristic	Level	No. (%)			
		Infants/toddlers aged 0-2 y [n = 472]		Preschool-aged children 3-5 y [n = 539]	
		Infected (n = 278)	Uninfected (n = 194)	Infected (n = 399)	Uninfected (n = 140)
Age, mean (SD), mo	NA	14 (9)	10 (10)	48 (10)	49 (10)
Sex assigned at birth	Female	135 (49)	102 (53)	198 (50)	79 (56)
	Male	142 (51)	92 (47)	201 (50)	61 (44)
	Intersex	1 (0)	0	0	0
Race and ethnicity^a^	American Indian or Alaska Native	7 (3)	11 (6)	9 (2)	3 (2)
	Asian	18 (7)	16 (8)	36 (9)	9 (7)
	Black or African American	40 (15)	33 (17)	54 (14)	16 (12)
	Hispanic, Latino, or Spanish	121 (44)	77 (40)	157 (39)	53 (39)
	Native Hawaiian or Other Pacific Islander	0	0	0	1 (1)
	White	139 (51)	103 (54)	214 (54)	73 (53)
	None of these fully describe me	4 (1)	1 (1)	1 (0)	2 (1)
	Missing	4	3	1	3
English is primary language	Yes	211 (80)	145 (81)	325 (83)	110 (83)
	No	54 (20)	34 (19)	67 (17)	23 (17)
	Missing	13	15	7	7
Birth in the US	Yes	273 (99)	187 (97)	382 (98)	135 (97)
	No	3 (1)	5 (3)	7 (2)	4 (3)
	Missing	2	2	10	1
Population referral source	Existing non-COVID research or clinical cohort	18 (6)	20 (10)	38 (10)	12 (9)
	Community outreach	86 (31)	61 (31)	110 (28)	48 (34)
	Participant tested and/or treated in the health system	79 (28)	27 (14)	106 (27)	15 (11)
	Self-referral from RECOVER website or other unsolicited self-referral	33 (12)	41 (21)	68 (17)	37 (26)
	Community health center	30 (11)	29 (15)	50 (13)	15 (11)
	Public health department list	1 (0)	0	4 (1)	1 (1)
	Long COVID clinic	2 (1)	1 (1)	2 (1)	0
	Existing, prospectively followed COVID cohort	18 (6)	5 (3)	9 (2)	4 (3)
	Other	11 (4)	10 (5)	12 (3)	8 (6)
From a medically underserved area	Yes	134 (48)	93 (48)	178 (45)	71 (51)
From a rural area	Yes	8 (3)	6 (3)	16 (4)	9 (6)
Prevalent SARS-CoV-2 strain at date of first infection^b^	Pre-Omicron	55 (20)	NA	196 (49)	NA
	Omicron	223 (80)	NA	203 (51)	NA
Vaccination status at first infection (infected) or enrollment (uninfected)^c^	Not eligible for vaccination	103 (37)	0	311 (78)	0
	Not vaccinated	138 (50)	146 (75)	48 (12)	73 (53)
	Vaccinated but missing information	6 (2)	5 (3)	2 (1)	10 (7)
	Partially vaccinated	27 (10)	16 (8)	14 (4)	18 (13)
	Fully vaccinated	4 (1)	27 (14)	24 (6)	38 (27)
Time between first infection (infected) or March 1, 2020 (uninfected) and symptom survey, median (IQR), d	NA	318 (198-494)	824 (692-930)	520 (330-844)	872 (642-985)
Primary caregiver characteristics					
Relationship to child	Mother	252 (93)	173 (91)	370 (95)	121 (92)
Educational attainment	College or higher	139 (59)	92 (55)	199 (60)	69 (61)

Abbreviation: NA, not applicable.

^a^ Race and ethnicity were caregiver reported, and participants were asked to select all that apply. Additional details can be found in eMethods in the Supplement 1.

^b^ The prevalent SARS-CoV-2 strain at date of first infection was classified as Pre-Omicron if the date of the first infection was before December 1, 2021, and Omicron if it was December 1, 2021, or later.

^c^ Because first infection date precedes enrollment for infected participants, some infected participants were not eligible for vaccination at enrollment. Additional description of how these categories are defined can be found in eMethods in the Supplement 1.

Overall, 114 of 278 infants/toddlers (41%) with infection and 49 of 194 infants/toddlers (25%) without infection had at least 1 prolonged symptom, and 179 of 399 preschool-aged children (45%) with infection and 52 of 140 preschool-aged children (37%) without infection had at least 1 prolonged symptom. Nine symptoms in infants/toddlers and 16 symptoms in preschool-age children were prolonged in at least 5% of infected young children (eFigure 2 in Supplement 1). The lower 95% confidence bound of the adjusted risk difference between infected and uninfected status exceeded zero for 5 of these symptoms in only infants/toddlers (trouble sleeping, fussiness, poor appetite, stuffy nose, wet cough), for daytime tiredness/sleepiness or low energy in only preschool-aged children, and for dry cough in both age groups (eFigure 2 in Supplement 1). The proportion of children experiencing each symptom is listed in eTable 7 in Supplement 1.

**Figure 1.**
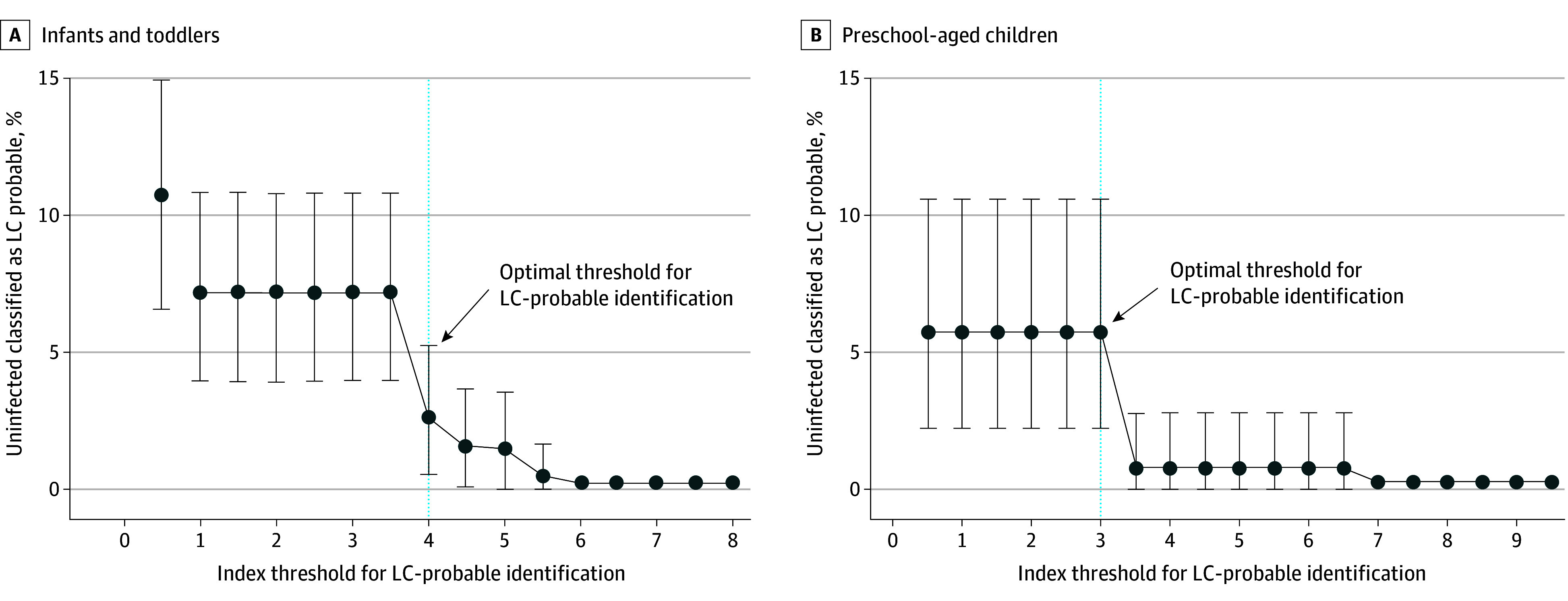
Identification of the Optimal Threshold for Long COVID (LC) Using the LC Research Index A, Infants and toddlers. B, Preschool-aged children. The optimal research index threshold for LC was selected to distinguish between participants with probable LC and unspecified LC using bootstrapping to estimate error bars (95% CIs shown). The dashed horizontal line and shading indicates the threshold for optimal identification of LC-probable status. An approximation of the elbow method was used to identify the cutoff where the number of uninfected participants misclassified as having probable LC stabilized (eMethods in the Supplement 1). The threshold, an index of 4 or greater in infants/toddlers and 3 or greater in preschool-aged children, can be used to identify young children with LC for research purposes. Using this threshold, the percentage of infected infants and toddlers with probable LC with each symptom was as follows: poor appetite, 52%; trouble sleeping, 28%; wet cough, 45%; dry cough, 25%; and stuffy nose, 57%. The percentage of infected preschool-aged children with probable LC with each symptom was as follows: daytime tiredness/sleepiness or low energy, 33%; and dry cough, 74%.

**Figure 2.**
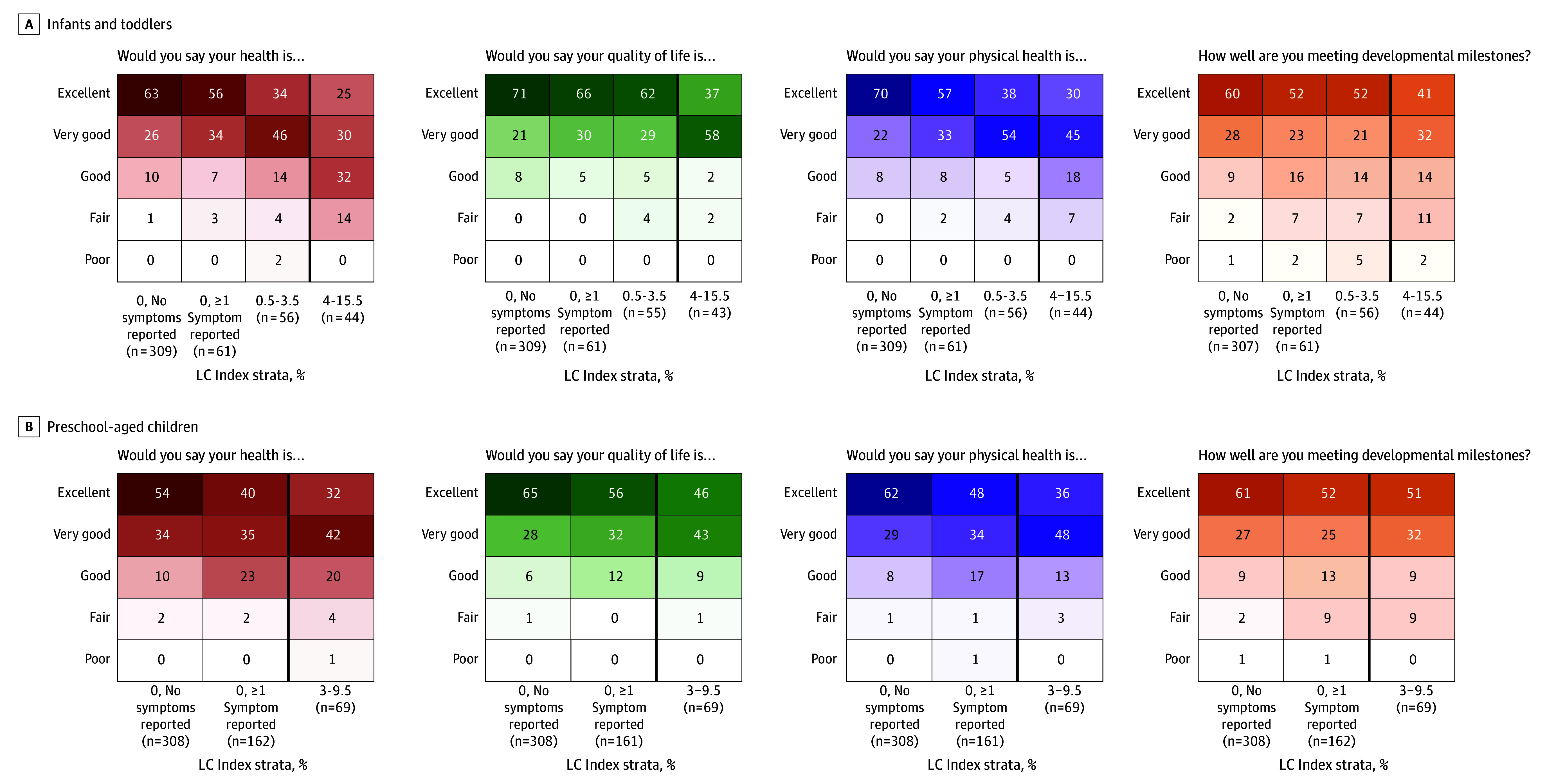
Long COVID (LC) Research Index and Patient-Reported Outcomes Measurement Information System (PROMIS) Early Childhood Parent Report Global Health Scales A, Infants and toddlers. B, Preschool-aged children. Caregiver responses to 4 questions from the PROMIS early childhood parent report global health scales survey were reported, stratified into groups. Two groups are the same for both age groups: participants with a zero LC research index and no prolonged symptoms and participants with a zero LC research index but at least 1 prolonged symptom that does not contribute to the index. In infants and toddlers, there are 2 additional groups: those with nonzero research indices but not meeting the threshold for LC (0.5-3.5), and those with research indices that meet the threshold (4-15.5, the maximum). In preschool-aged children, there is 1 additional group: participants with either of the 2 symptoms that contribute to the LC research index, thus reaching the threshold for LC (3-9.5). The thick vertical black line indicates the threshold for LC. The shading in each cell is proportional to the reported frequency of each response.

LASSO identified 5 symptoms in infants/toddlers and 2 in preschool-aged children that were most related to infection history (Table 2). The index is calculated by summing scores assigned to each prolonged symptom that was present, where higher scores indicate greater magnitude of association with history of SARS-CoV-2 infection: poor appetite (5 points), trouble sleeping (3.5 points), wet cough (3.5 points), dry cough (3 points), and stuffy nose (0.5 points) for infants/toddlers, and daytime tiredness/sleepiness/low energy (6.5 points) and dry cough (3 points) for preschool-aged children. We identified an optimal index threshold (ie, total score) of 4 in infants/toddlers and 3 in preschool-aged children (Figure 1). In preschool-aged children, because the symptom with the lowest score (dry cough) had a score of 3, having either symptom (dry cough or daytime tiredness/sleepiness or low energy) met the threshold. Overall, 40 of 278 infants/toddlers (14%) with infection and 5 of 194 infants/toddlers (3%) without infection and 61 of 399 preschool-aged children (15%) with infection and 8 of 140 preschool-aged children (6%) without infection met or exceeded these index thresholds (eTable 5 in Supplement 1). This proportion was higher for infants/toddlers whose infection was before Omicron vs after Omicron (11 of 55 infants/toddlers [20%] vs 29 of 223 infants/toddlers [13%]) and similar for preschool-aged children (30 of 196 children [15%] vs 31 of 203 children [15%]). eFigure 3 in Supplement 1 presents correlations between symptoms in the index. Correlations between symptoms included and not included in the indices are described in eTable 6 in Supplement 1. In both age groups, those with at least 1 symptom and those whose indices were nonzero tended to have worse PROMIS scores for overall health, quality of life, physical health, and developmental milestones (Figure 2).

**Table 2.** The Long COVID (LC) Research Index^a^

Age group	Symptom	Log odds ratio	Score
Infants/toddlers (0-2 y)	Poor appetite	0.47	5.0
	Trouble sleeping	0.34	3.5
	Wet cough	0.32	3.5
	Dry cough	0.27	3.0
	Stuffy nose	0.05	0.5
Preschool-aged children (3-5 y)	Daytime tiredness/sleepiness or low energy	0.64	6.5
	Dry cough	0.28	3.0

^a^ To fit a logistic regression model to identify which symptoms could be used to identify which participants have LC-probable vs LC-unspecified status, least absolute shrinkage and selection operator (LASSO) was used, resulting in these log odds ratio estimates. In this model, the symptoms were the exposures, and the outcome was infection status. The log odds ratio estimates were divided by 0.10 and rounded up to the nearest 0.5 to calculate individual symptom scores. An individual’s LC research index is calculated by summing the scores for each prolonged symptom a participant reported. A prolonged symptom is defined as a symptom that the participant experienced for at least 4 weeks since the beginning of the COVID-19 pandemic and is still experiencing at the time of enrollment. The index is derived separately for infants/toddlers and preschool-aged children.

Table 3 presents the proportion of children in each age range stratified into 3 groups based on their infection history and LC status (LC-probable status, LC-unspecified status, and no infection) who are experiencing each symptom. The most common prolonged symptoms in infants/toddlers with LC-probable status that contributed to the index were stuffy nose (23 of 40 [57%]), poor appetite (21 of 40 [52%]), and wet cough (18 of 40 [45%]). Of the symptoms that did not contribute to the index, fussiness (11 of 40 [28%]) and trouble sleeping (11 of 40 [28%]) were the most common. Daytime tiredness/sleepiness or low energy was only assessed in the preschool-aged children and could not contribute to the infant/toddler index. A heatmap for these results is provided in eFigure 4 in Supplement 1.

**Table 3.** Frequency of Prolonged Symptoms Among Infants and Toddlers and Preschool-Aged Children Stratified by Infection and Long COVID (LC) Status^a^

Symptom	Infants and toddlers aged 0-2 y, No./total No. (%)			Preschool-aged children 3-5 y, No./total No. (%)		
	Uninfected (n = 194)	Infected, LC-unspecified (n = 238)	Infected, LC-probable (n = 40)	Uninfected (n = 140)	Infected, LC-unspecified (n = 338)	Infected, LC-probable (n = 61)
Hyperactivity, refusing to follow rules, or frequent tantrums	13/194 (7)	13/238 (5)	8/40 (20)	27/140 (19)	46/338 (14)	18/61 (30)
Stuffy nose^b^	13/194 (7)	17/238 (7)	23/40 (57)	10/140 (7)	30/338 (9)	21/61 (34)
Phobias, separation anxiety, or fear of others	15/194 (8)	11/238 (5)	9/40 (22)	15/140 (11)	31/338 (9)	18/61 (30)
Dry cough^b^^,^^c^	3/194 (2)	9/238 (4)	10/40 (25)	7/140 (5)	0/338	45/61 (74)
Fussy	5/194 (3)	10/238 (4)	11/40 (28)	7/140 (5)	26/338 (8)	11/61 (18)
Wet cough^b^	4/194 (2)	5/238 (2)	18/40 (45)	8/140 (6)	16/338 (5)	16/61 (26)
Poor appetite^b^	3/194 (2)	0/238	21/40 (52)	6/140 (4)	17/338 (5)	14/61 (23)
Trouble sleeping^b^	4/194 (2)	11/238 (5)	11/40 (28)	7/140 (5)	17/338 (5)	11/61 (18)
Itchy skin or skin rash	7/194 (4)	13/238 (5)	5/40 (12)	7/140 (5)	19/338 (6)	9/61 (15)
Serious rule breaking or aggressive behaviors	4/194 (2)	8/238 (3)	3/40 (8)	13/140 (9)	22/338 (7)	6/61 (10)
Nightmares or night terrors	3/194 (2)	3/238 (1)	8/40 (20)	7/140 (5)	19/338 (6)	11/61 (18)
Tired after walking	NA	NA	NA	4/140 (3)	8/338 (2)	13/61 (21)
Dark circles	2/194 (1)	6/238 (3)	5/40 (12)	6/140 (4)	9/338 (3)	13/61 (21)
Daytime tiredness/sleepiness or low energy^c^	NA	NA	NA	1/140 (1)	0/338	20/61 (33)
Trouble with memory or focusing	NA	NA	NA	4/140 (3)	8/338 (2)	9/61 (15)
Increased thirst	0/194	8/238 (3)	3/40 (8)	7/140 (5)	13/338 (4)	7/61 (11)
Sore throat	NA	NA	NA	1/140 (1)	6/338 (2)	12/61 (20)
Body, muscle, or joint pain	NA	NA	NA	3/140 (2)	8/338 (2)	8/61 (13)
Constipation	2/194 (1)	4/238 (2)	3/40 (8)	4/140 (3)	15/338 (4)	5/61 (8)
Chapped lips	2/194 (1)	3/238 (1)	4/40 (10)	5/140 (4)	9/338 (3)	8/61 (13)
Lost weight	2/194 (1)	6/238 (3)	4/40 (10)	3/140 (2)	8/338 (2)	7/61 (11)
Stomach pain	NA	NA	NA	4/140 (3)	6/338 (2)	5/61 (8)
Barking cough	1/194 (1)	2/238 (1)	5/40 (12)	2/140 (1)	1/338 (0)	16/61 (26)
Fever	1/194 (1)	3/238 (1)	6/40 (15)	1/140 (1)	6/338 (2)	10/61 (16)
Trouble breathing	1/194 (1)	1/238 (0)	7/40 (18)	1/140 (1)	3/338 (1)	12/61 (20)
Diarrhea	4/194 (2)	4/238 (2)	2/40 (5)	3/140 (2)	6/338 (2)	6/61 (10)
Excess sweat	1/194 (1)	3/238 (1)	7/40 (18)	3/140 (2)	4/338 (1)	6/61 (10)
Rocking back and forth	4/194 (2)	7/238 (3)	3/40 (8)	2/140 (1)	6/338 (2)	2/61 (3)
Nausea or vomiting	2/194 (1)	3/238 (1)	5/40 (12)	0/140	3/338 (1)	10/61 (16)
Watery or red eyes	1/194 (1)	2/238 (1)	3/40 (8)	3/140 (2)	6/338 (2)	7/61 (11)

Abbreviation: NA, not applicable.

^a^ Symptoms are sorted from most to least common in the study population overall, and symptoms with frequency of at least 2% overall are shown. Proportions and percentages are NA for symptoms that are not asked in a given age group, and missing symptom responses are treated in this table as absent. A heatmap of these results for all 56 symptoms is provided in eFigure 4 in Supplement 1.

^b^ This symptom contributes to the LC research index in infants and toddlers.

^c^ This symptom contributes to the LC research index in preschool-aged children.

Among preschool-aged children with LC-probable status, 45 of 61 (74%) experienced dry cough, and 20 of 61 (33%) reported daytime tiredness/sleepiness or low energy (Table 3). Of the symptoms that did not contribute to the index, stuffy nose (21 of 61 children [34%]), hyperactivity, refusing to follow rules, or frequent tantrums (18 of 61 children [30%]), and phobias, separation anxiety, or fear of others (18 of 61 children [30%]) were most common. In both age groups, symptom reports were similar between the children in the LC-unspecified and uninfected groups.

In the sensitivity analysis, we identified 60 of 140 preschool-aged children (43%) without infection who were confirmed nucleocapsid SARS-CoV-2–antibody negative, with the remainder having missing antibody data. Of those children, 4 of 60 (7%) met criteria for LC-probable status according to the index, compared with 8 of 140 (6%) of the full cohort without infection (nucleocapsid SARS-CoV-2–antibody negative plus antibody missing).

The distribution of the total systems affected in young children with LC-probable status is in eFigure 5 in Supplement 1. Age-standardized probability curves for each symptom contributing to the index are in eFigure 6 in Supplement 1, which demonstrate that there was generally no association between the prolonged symptoms and time elapsed between first infection and symptom survey for most symptoms.

## Discussion

This was the first, to our knowledge, large multisite study to characterize prolonged symptoms after SARS-CoV-2 infection in early childhood age groups (infants/toddlers and preschool age). Symptom patterns in infants/toddlers differed from those of preschool-aged children, demonstrating the need to characterize LC separately in these young age groups. Both symptom patterns differed from those previously identified in older children and adults. Given that most LC research has focused on adults and older children, these findings make a substantial contribution by focusing on the earliest periods of the life course.^9,42,43^

This analysis developed 2 empirically derived indices that aid researchers in identifying young children with LC. Although these indices included only a few symptoms (5 for infants/toddlers; 2 for preschool-aged children), children identified by the index as likely to have LC have many other symptoms that do not contribute to the index. Additionally, it was uncommon for children who did not meet the threshold to have other prolonged symptoms. Although higher indices appear to be associated with lower overall health, quality of life, physical health, and developmental milestones, the research index is not designed for clinical purposes. These indices will be useful in future studies to identify clinical and social risk factors in early childhood for developing LC, which may be different than in older groups, and patterns of symptom relapse and remission, persistence, and/or progression.

This analysis identified separate indices for infants/toddlers vs preschool-aged children, based on symptoms likely to differentiate between young children with and without SARS-CoV-2 infection history. In previous RECOVER-Pediatrics analyses,^21,30^ the symptoms included in the school-aged index were very different from those found in early childhood, with more predominance of neurological, dermatological, and gastrointestinal symptoms, as well as pain and fears. The adolescent index included symptoms that were more like symptoms included in the adult index, with less overlap between the adult and school-aged indices. These differences highlight why it is imperative to conduct separate assessments in different age groups. The pathophysiology and mechanisms leading to these age-related differences warrant further investigation. The findings that infants/toddlers and preschool-aged children have varied symptoms may be explained by the fact that symptoms in younger children are reported based on what caregivers can observe rather than what the children themselves are feeling and describing, because most children in this age group do not yet have the language, social skills, or understanding of symptoms to share what they are experiencing. For example, fears and feelings of pain, brain fog, headache, tiredness, or changes in taste and smell may be hard to identify if the child cannot verbalize their internal feelings or sensations, whereas a symptom such as cough is easily observed. A further complication is that the identified symptoms may occur commonly in young children because of their naive immune systems. Daytime sleepiness, trouble sleeping, cough, stuffy nose, and poor appetite can occur in many acute and chronic early childhood illnesses.^20^ A previous study^4^ comparing children between 0 and 18 years who had a previous COVID-19 infection with those diagnosed with a different communicable illness (no COVID-19 history) found that persistent symptoms were significantly more likely with COVID-19 infection, with fatigue, irritability and mood changes, headaches, runny nose, cough, and loss of smell and taste being the most common. Of note, the most reported persistent symptoms in other studies that included children of all ages were fatigue or malaise, consistent with our findings in the preschool-aged group.^4,13,44^

In early childhood, both indices were related to caregiver perceptions of health and development. Few prior studies have examined associations between SARS-CoV-2 and developmental delays, especially in infants/toddlers. One small study^45^ that used a standardized test (Bayley Scales of Infant Development-III) reported developmental delays in toddlers who were infected with SARS-CoV-2 as neonates. Ongoing RECOVER-Pediatrics research will test these associations longitudinally using objective measures of neurodevelopmental domains, including memory, attention, sensory function, receptive and expressive language skills, and reading, as well as measures of daily functioning beyond the PROMIS global health measures.^46^ Given RECOVER-Pediatrics’ inclusion of children with and without a history of infection, analyses will be able to differentiate changes related to the COVID-19 pandemic broadly and those related specifically to SARS-CoV-2 infection.^47^

### Limitations

This study has some limitations. The indices are intended for research to identify infants, toddlers, and preschool-aged children with probable LC status, and not as a clinical screening tool. This index is not meant to determine incidence, as an individual’s status may fluctuate over time. The index can, however, be used to follow recovery and relapse over time, which would be impossible if resolved symptoms were included. Given that families with young children with LC symptoms might be more likely to enroll in a study about LC, population prevalence cannot be determined. In addition, it is unknown whether caregiver concern about certain symptoms more than others could elevate enrollment of children with those more worrisome symptoms. Of note, RECOVER-Pediatrics did find comparable rates of LC for infants/toddlers (14%), preschool-aged children (15%), school-aged children (20%), and adolescents (14%) with infection.^21^ In addition, there were many prolonged symptoms not included in the indices, and we acknowledge that young children with these other symptoms may ultimately have or progress to develop LC. These indices may change with time, particularly with new SARS-CoV-2 variants, population immunity, reinfections, and with the transition from infancy/toddlerhood to the preschool age. If a symptom persisted more than 4 weeks but was resolved or remitted at the time of the survey, it was not included in these analyses. Given the subjective nature of caregiver-reported symptoms, future studies will benefit from studying how these prolonged symptoms are associated with objective assessments and biomarkers.

It should be noted that groups with and without infection are subject to misclassification, because it was not possible to confirm the presence or absence of SARS-CoV-2 antibodies in all participants in this younger age group. Children with prolonged symptoms who were assumed to have LC may have had other symptomatic respiratory viruses, given that tests for other viruses were not performed. When we restricted the group without infection to those who were confirmed to be antibody negative, the rate of LC was similar to that of participants without infection overall, suggesting that participants without infection and without confirmed antibody status were similar in terms of rates of LC. Furthermore, recall bias is possible given that caregivers may have missed or misinterpreted their young child’s symptoms, especially with the long symptom duration experienced by some children. Despite potential for misclassification, groups with and without infection demonstrated clear differences. Finally, due to sample size and limited number of persistent symptoms associated with LC in these age groups, we were not able to explore symptom clusters as previously identified for older groups. Future studies should aim to enroll larger samples beginning in early childhood, enhancing power needed to detect subtle symptom differences and evaluate generalizability of the index in other populations.

## Conclusions

In this cohort study, these findings extend evidence documenting different LC symptoms between school-aged children, adolescents, and adults to earlier periods in the life course, including infants, toddlers, and preschool-aged children. The research indices may aid researchers in identifying young children with LC. These findings support the concept that a one-size-fits-all approach to screening for LC across the lifespan is not possible and will likely need to be tailored for specific age groups.

## Notes

Supplement 1.
eMethods.

eReferences.
**eTable 1.** List of Sites in RECOVER-Pediatrics **eTable 2.** Tier 1 Symptom Survey Questions **eTable 3.** Symptom Groupings and Correlations, Calculated Among All Participants **eTable 4.** First COVID Infection History Characteristics of Infected Participants, by Age Group **eTable 5.** Long COVID Frequencies, Stratified by Age Group, Infection Status, and Era of Infection **eTable 6.** Correlation Between Symptoms Contributing to the Long COVID Research Index and Those That Do Not **eTable 7.** Proportion of Participants Experiencing Each of All 75 Original Prolonged Symptoms in Each Age Group **eFigure 1.** Application of RECOVER-Pediatrics Analysis Cohort Inclusion and Exclusion Criteria **eFigure 2.** Participants With Each Prolonged Symptom, Adjusted Odds Ratios, and Adjusted Risk Differences Comparing Infected vs Uninfected Participants **eFigure 3.** Correlation Matrix of Long COVID Symptoms Among Infected Long COVID–Probable Infants and Toddlers and Preschool-Aged Children **eFigure 4.** Heatmap Showing Frequency of Prolonged Symptoms Stratified by Infection and Long COVID Status **eFigure 5.** Number of Systems Affected Among Infected Long COVID–Probable Participants **eFigure 6.** Estimated Probability of the Presence of Each Prolonged Symptom Contributing to the Long COVID Research Indices, After Age Standardization

Supplement 2. **Nonauthor Collaborators.** RECOVER-Pediatrics Consortium.

Supplement 3.
Data Sharing Statement.

## References

[1] World Health Organization. Post COVID-19 condition (long COVID). Accessed February 27, 2024. https://www.who.int/europe/news-room/fact-sheets/item/post-covid-19-condition

[2] Centers for Disease Control and Prevention (CDC). Long COVID basics. Accessed September 28, 2023. https://www.cdc.gov/coronavirus/2019-ncov/long-term-effects/index.html

[3] National Academies of Sciences, Engineering, and Medicine. A Long COVID Definition: A Chronic, Systemic Disease State With Profound Consequences. The National Academies Press; 2024. 39110819

[4] Roge I, Smane L, Kivite-Urtane A, . Comparison of persistent symptoms after COVID-19 and other non–SARS-CoV-2 infections in children. Front Pediatr. 2021;9:752385. doi:10.3389/fped.2021.752385 34778143 PMC8586002

[5] Behnood SA, Shafran R, Bennett SD, . Persistent symptoms following SARS-CoV-2 infection amongst children and young people: a meta-analysis of controlled and uncontrolled studies. J Infect. 2022;84(2):158-170. doi:10.1016/j.jinf.2021.11.011 34813820 PMC8604800

[6] Buonsenso D, Pazukhina E, Gentili C, . The prevalence, characteristics and risk factors of persistent symptoms in nonhospitalized and hospitalized children with SARS-CoV-2 infection followed-up for up to 12 months: a prospective, cohort study in Rome, Italy. J Clin Med. 2022;11(22):6772. doi:10.3390/jcm11226772 36431250 PMC9692851

[7] Funk AL, Kuppermann N, Florin TA, ; Pediatric Emergency Research Network–COVID-19 Study Team. Post–COVID-19 conditions among children 90 days after SARS-CoV-2 infection. JAMA Netw Open. 2022;5(7):e2223253. doi:10.1001/jamanetworkopen.2022.23253 35867061 PMC9308058

[8] Hernandez-Romieu AC, Carton TW, Saydah S, . Prevalence of select new symptoms and conditions among persons aged younger than 20 years and 20 years or older at 31 to 150 days after testing positive or negative for SARS-CoV-2. JAMA Netw Open. 2022;5(2):e2147053. doi:10.1001/jamanetworkopen.2021.47053 35119459 PMC8817203

[9] Lopez-Leon S, Wegman-Ostrosky T, Ayuzo Del Valle NC, . Long-COVID in children and adolescents: a systematic review and meta-analyses. Sci Rep. 2022;12(1):9950. doi:10.1038/s41598-022-13495-5 35739136 PMC9226045

[10] Miller F, Nguyen DV, Navaratnam AM, ; VirusWatch Collaborative. Prevalence and characteristics of persistent symptoms in children during the COVID-19 pandemic: evidence from a household cohort study in England and Wales. Pediatr Infect Dis J. 2022;41(12):979-984. doi:10.1097/INF.0000000000003715 36375098 PMC9645448

[11] Pellegrino R, Chiappini E, Licari A, Galli L, Marseglia GL. Prevalence and clinical presentation of long COVID in children: a systematic review. Eur J Pediatr. 2022;181(12):3995-4009. doi:10.1007/s00431-022-04600-x 36107254 PMC9476461

[12] Rao S, Lee GM, Razzaghi H, . Clinical features and burden of postacute sequelae of SARS-CoV-2 infection in children and adolescents. JAMA Pediatr. 2022;176(10):1000-1009. doi:10.1001/jamapediatrics.2022.2800 35994282 PMC9396470

[13] Roessler M, Tesch F, Batram M, . Post–COVID-19-associated morbidity in children, adolescents, and adults: a matched cohort study including more than 157 000 individuals with COVID-19 in Germany. PLoS Med. 2022;19(11):e1004122. doi:10.1371/journal.pmed.1004122 36355754 PMC9648706

[14] Stephenson T, Shafran R, Ladhani SN. Long COVID in children and adolescents. Curr Opin Infect Dis. 2022;35(5):461-467. doi:10.1097/QCO.0000000000000854 36098262 PMC9553244

[15] Lorman V, Rao S, Jhaveri R, . Understanding pediatric long COVID using a tree-based scan statistic approach: an EHR-based cohort study from the RECOVER program. JAMIA Open. 2023;6(1):ooad016. doi:10.1093/jamiaopen/ooad016 36926600 PMC10013630

[16] Lorman V, Razzaghi H, Song X, . A machine learning-based phenotype for long COVID in children: an EHR-based study from the RECOVER program. PLoS One. 2023;18(8):e0289774. doi:10.1371/journal.pone.0289774 37561683 PMC10414557

[17] Morello R, Mariani F, Mastrantoni L, . Risk factors for post–COVID-19 condition (long COVID) in children: a prospective cohort study. EClinicalMedicine. 2023;59:101961. doi:10.1016/j.eclinm.2023.101961 37073325 PMC10101848

[18] Vahratian A, Adjaye-Gbewonyo D, Lin JS, Saydah S. Long COVID in children: US, 2022. NCHS Data Brief. 2023;(479):1-6. doi:10.15620/cdc:132416 37756128

[19] Sansone F, Pellegrino GM, Caronni A, . Long COVID in children: a multidisciplinary review. Diagnostics (Basel). 2023;13(12):1990. doi:10.3390/diagnostics13121990 37370884 PMC10297324

[20] Rao S, Gross RS, Mohandas S, . Postacute sequelae of SARS-CoV-2 in children. Pediatrics. 2024;153(3):e2023062570. doi:10.1542/peds.2023-062570 38321938 PMC10904902

[21] Gross RS, Thaweethai T, Kleinman LC, ; RECOVER-Pediatrics Consortium; RECOVER-Pediatrics Group Authors. Characterizing long COVID in children and adolescents. JAMA. 2024;332(14):1174-1188. doi:10.1001/jama.2024.12747 39196964 PMC11339705

[22] Kikkenborg Berg S, Dam Nielsen S, Nygaard U, . Long COVID symptoms in SARS-CoV-2-positive adolescents and matched controls (LongCOVIDKidsDK): a national, cross-sectional study. Lancet Child Adolesc Health. 2022;6(4):240-248. doi:10.1016/S2352-4642(22)00004-9 35143771 PMC8820960

[23] Scionti N, Zampini L, Marzocchi GM. The relationship between narrative skills and executive functions across childhood: a systematic review and meta-analysis. Children (Basel). 2023;10(8):1391. doi:10.3390/children10081391 37628390 PMC10453360

[24] Bhimani P, Scheinfeld A, Rajan M. Emergency department utilization among children with long COVID symptoms: a COVID-19 research consortium study. BMC Pediatr. 2024;24(1):635. doi:10.1186/s12887-024-04817-9 39369205 PMC11452963

[25] Borch L, Holm M, Knudsen M, Ellermann-Eriksen S, Hagstroem S. Long COVID symptoms and duration in SARS-CoV-2 positive children—a nationwide cohort study. Eur J Pediatr. 2022;181(4):1597-1607. doi:10.1007/s00431-021-04345-z 35000003 PMC8742700

[26] Jaswa EG, Huddleston HG, Lindquist KJ, . In utero exposure to maternal COVID-19 and offspring neurodevelopment through age 24 months. JAMA Netw Open. 2024;7(10):e2439792. doi:10.1001/jamanetworkopen.2024.39792 39412802 PMC11581627

[27] Vrantsidis DM, van de Wouw M, Hall ERM, . Neurodevelopment in the first 2 years of life following prenatal exposure to maternal SARS-CoV-2 infection. JAMA Netw Open. 2024;7(11):e2443697. doi:10.1001/jamanetworkopen.2024.43697 39509130 PMC11544495

[28] Thomas Boyce W, Hertzman C. Early childhood health and the life course: the state of the science and proposed research priorities: a background paper for the MCH Life Course Research Network. In: Halfon N, Forrest CB, Lerner RM, Faustman EM, eds. Handbook of Life Course Health Development. Springer; 2018:61-93. 31314305

[29] RECOVER. RECOVER: researching COVID to enhance recovery. Accessed January 29, 2025. https://recovercovid.org/

[30] Gross RS, Thaweethai T, Rosenzweig EB, ; RECOVER-Pediatric Consortium. Researching COVID to Enhance Recovery (RECOVER) pediatric study protocol: rationale, objectives and design. PLoS One. 2024;19(5):e0285635. doi:10.1371/journal.pone.0285635 38713673 PMC11075869

[31] Thaweethai T, Jolley SE, Karlson EW, ; RECOVER Consortium. Development of a definition of postacute sequelae of SARS-CoV-2 infection. JAMA. 2023;329(22):1934-1946. doi:10.1001/jama.2023.8823 37278994 PMC10214179

[32] Geng LN, Erlandson KM, Hornig M, ; RECOVER Consortium. 2024 Update of the RECOVER-adult long COVID research index. JAMA. 2025;333(8):694-700. doi:10.1001/jama.2024.24184 39693079 PMC11862971

[33] Osmanov IM, Spiridonova E, Bobkova P, ; and the Sechenov StopCOVID Research Team. Risk factors for post–COVID-19 condition in previously hospitalized children using the ISARIC Global follow-up protocol: a prospective cohort study. Eur Respir J. 2022;59(2):2101341. doi:10.1183/13993003.01341-2021 34210789 PMC8576804

[34] World Health Organization. A Clinical case definition for post–COVID-19 condition in children and adolescents by expert consensus. February 16, 2023. Accessed December 26, 2024. https://www.who.int/publications/i/item/WHO-2019-nCoV-Post-COVID-19-condition-CA-Clinical-case-definition-2023-1

[35] Al-Aly Z, Davis H, McCorkell L, . Long COVID science, research, and policy. Nat Med. 2024;30(8):2148-2164. doi:10.1038/s41591-024-03173-6 39122965

[36] Davis TC, Gazmararian J, Kennen EM. Approaches to improving health literacy: lessons from the field. J Health Commun. 2006;11(6):551-554. doi:10.1080/10810730600835517 16950727

[37] Forrest CB, Bevans KB, Pratiwadi R, . Development of the PROMIS pediatric global health (PGH-7) measure. Qual Life Res. 2014;23(4):1221-1231. doi:10.1007/s11136-013-0581-8 24264804 PMC3966936

[38] Kallen MA, Lai JS, Blackwell CK, . Measuring PROMIS global health in early childhood. J Pediatr Psychol. 2022;47(5):523-533. doi:10.1093/jpepsy/jsac026 35552435 PMC9113277

[39] Reeder HT, Thaweethai T, Foulkes AS. Penalized regression with negative-unlabeled data: An approach to developing a long COVID research index. arXiv. Preprint posed online October 9, 2024. doi:10.48550/arXiv.2410.07357 PMC12802229 41361800

[40] Hastie T, Tibshirani R, Friedman J. The Elements of Statistical Learning: Data Mining, Inference, and Prediction. 2nd ed. Springer; 2009.

[41] R Development Core Team. R: A Language and Environment for Statistical Computing. R Foundation for Statistical Computing; 2024.

[42] Davis HE, McCorkell L, Vogel JM, Topol EJ. Long COVID: major findings, mechanisms and recommendations. Nat Rev Microbiol. 2023;21(3):133-146. doi:10.1038/s41579-022-00846-2 36639608 PMC9839201

[43] Groff D, Sun A, Ssentongo AE, . Short-term and long-term rates of postacute sequelae of SARS-CoV-2 infection: a systematic review. JAMA Netw Open. 2021;4(10):e2128568. doi:10.1001/jamanetworkopen.2021.28568 34643720 PMC8515212

[44] Pazukhina E, Rumyantsev M, Baimukhambetova D, ; Sechenov StopCOVID Research Team. Event rates and incidence of post–COVID-19 condition in hospitalized SARS-CoV-2–positive children and young people and controls across different pandemic waves: exposure-stratified prospective cohort study in Moscow (StopCOVID). BMC Med. 2024;22(1):48. doi:10.1186/s12916-023-03221-x 38302974 PMC10835884

[45] Goyal M, Mascarenhas D, Rr P, Nanavati R. Long-term growth and neurodevelopmental outcomes of neonates infected with SARS-CoV-2 during the COVID-19 pandemic at 18-24 months corrected age: a prospective observational study. Neonatology. 2024;121(4):450-459. doi:10.1159/000537803 38583433 PMC11318580

[46] Seylanova N, Chernyavskaya A, Degtyareva N, ; PC-COS Children Study Group. Core outcome measurement set for research and clinical practice in post–COVID-19 condition (long COVID) in children and young people: an international Delphi consensus study “PC-COS Children.” Eur Respir J. 2024;63(3):2301761. doi:10.1183/13993003.01761-2023 38359962 PMC10938351

[47] Lee KS, Choi YY, Kim YS, Kim Y, Kim MH, Lee N. Association between the COVID-19 pandemic and childhood development aged 30 to 36 months in South Korea, based on the national health screening program for infants and children database. BMC Public Health. 2024;24(1):989. doi:10.1186/s12889-024-18361-9 38594741 PMC11003091

