# Supplementary material for: Characterizing Long COVID Symptoms During Early Childhood
Source: JAMA Pediatr. 2025 May 27;179(7):781–92. doi: 10.1001/jamapediatrics.2025.1066 (PMC12117493; doi:10.1001/jamapediatrics.2025.1066)
Supplement: Supplement 1. — eMethods. eReferences. eTable 1. List of Sites in RECOVER-Pediatrics eTable 2. Tier 1 Symptom Survey Questions eTable 3. Symptom Groupings and Correlations, Calculated Among All Participants eTable 4. First COVID Infection History Characteristics of Infected Participants, by Age Group eTable 5. Long COVID Frequencies, Stratified by Age Group, Infection Status, and Era of Infection eTable 6. Correlation Between Symptoms Contributing to the Long COVID Research Index and Those That Do Not eTable 7. Proportion of Participants Experiencing Each of All 75 Original Prolonged Symptoms in Each Age Group eFigure 1. Application of RECOVER-Pediatrics Analysis Cohort Inclusion and Exclusion Criteria eFigure 2. Participants With Each Prolonged Symptom, Adjusted Odds Ratios, and Adjusted Risk Differences Comparing Infected vs Uninfected Participants eFigure 3. Correlation Matrix of Long COVID Symptoms Among Infected Long COVID–Probable Infants and Toddlers and Preschool-Aged Children eFigure 4. Heatmap Showing Frequency of Prolonged Symptoms Stratified by Infection and Long COVID Status eFigure 5. Number of Systems Affected Among Infected Long COVID–Probable Participants eFigure 6. Estimated Probability of the Presence of Each Prolonged Symptom Contributing to the Long COVID Research Indices, After Age Standardization [file jamapediatr-e251066-s001.pdf]

Supplementary Online Content

Gross RS, Thaweethai T, Salisbury AL, et al; RECOVER-Pediatrics Group Authors; RECOVER-Pediatrics Consortium. Characterizing long COVID symptoms during early childhood. *JAMA Pediatr*. Published online May 27, 2025.
doi:10.1001/jamapediatrics.2025.1066

eMethods.

eReferences.

eTable 1. List of Sites in RECOVER-Pediatrics

eTable 2. Tier 1 Symptom Survey Questions

eTable 3. Symptom Groupings and Correlations, Calculated Among All Participants

eTable 4. First COVID Infection History Characteristics of Infected Participants, by Age Group

eTable 5. Long COVID Frequencies, Stratified by Age Group, Infection Status, and Era of Infection

eTable 6. Correlation Between Symptoms Contributing to the Long COVID Research Index and Those That Do Not

eTable 7. Proportion of Participants Experiencing Each of All 75 Original Prolonged Symptoms in Each Age Group

eFigure 1. Application of RECOVER-Pediatrics Analysis Cohort Inclusion and Exclusion Criteria

eFigure 2. Participants With Each Prolonged Symptom, Adjusted Odds Ratios, and Adjusted Risk Differences Comparing Infected vs Uninfected Participants

eFigure 3. Correlation Matrix of Long COVID Symptoms Among Infected Long COVID–Probable Infants and Toddlers and Preschool-Aged Children

eFigure 4. Heatmap Showing Frequency of Prolonged Symptoms Stratified by Infection and Long COVID Status

eFigure 5. Number of Systems Affected Among Infected Long COVID–Probable Participants

eFigure 6. Estimated Probability of the Presence of Each Prolonged Symptom Contributing to the Long COVID Research Indices, After Age Standardization

This supplementary material has been provided by the authors to give readers additional information about their work.

© 2025 Gross RS et al. *JAMA Pediatrics*.

eMethods

Composite symptoms for analysis

The original symptom survey included 75 symptoms across 8 systems (**eTable 2**). Of these, 41 were asked of caregivers of infants and toddlers. Many of these 75 symptoms were identified by study clinicians as describing a similar phenotype. 14 composite symptoms were defined, with the correlations between symptoms that were grouped together reported in **eTable 3**. This resulted in the 37 symptoms considered in this analysis for infants and toddlers and 56 for preschool-age children.

Assessment of prolonged symptoms by enrollment cohort

Full details of the study design of RECOVER-Pediatrics have been published.[1] Here, we provide a summary of how prolonged symptoms were assessed in each of the relevant enrollment cohorts. For the full text of how the symptom surveys were worded, see **eTable 2**.

Infected participants enroll into the acute or post-acute arm depending on how much time has passed since their most recent infection. If no more than 30 days have passed, they are enrolled into the acute cohort; otherwise, they are enrolled into the post-acute cohort.

- Infected acute cohort participants are asked to complete symptom surveys 2, 4, and 8 weeks after enrollment, and then again at 6 months (with additional later follow-up). At 8 weeks, these participants are asked about symptoms that lasted 4 weeks or more since becoming infected. Then, at 6 months, these participants are again asked about symptoms that lasted 4 weeks or more since their last visit, and if so, whether the symptom is present now. Since follow-up is split between these two surveys, symptoms that were prolonged during only the first 8 weeks after infection but not between 8 weeks and 6 months would not be captured, <u>and so these participants were not included in the analysis</u>.

- Infected post-acute cohort participants are asked to complete symptom surveys at enrollment. They are asked about symptoms that lasted 4 weeks or more since their infection and whether the symptom is present now. If more than 90 days have passed since infection, they are included in this study.

Uninfected participants are randomized to the acute or post-acute cohort with a 1:5 ratio.

- Uninfected acute cohort participants are asked about symptoms at 2, 4, and 8 weeks after enrollment but are not assessed for prolonged symptoms since March 2020. They are not included in the analysis.

- Uninfected post-acute cohort participants are asked about symptoms that lasted for 4 weeks or more since their infection and whether the symptom is present now. They are all included in this study.

Race/ethnicity reporting and analysis

Respondents were asked: Which group(s) best describe [your child]? Please check all groups that describe [your child].

- o American Indian or Alaska Native
  (For example: Aztec, Blackfeet Tribe, Mayan, Navajo Nation, Native Village of Barrow (Utqiagvik) Inupiat Traditional Government, Nome Eskimo Community, etc.)
- o Asian
  (For example: Asian Indian, Chinese, Filipino, Japanese, Korean, Vietnamese, etc.)
- o Black or African American
  (For example: African American, Ethiopian, Haitian, Jamaican, Nigerian, Somali, etc.)
- o Hispanic, Latino, or Spanish
  (For example: Colombian, Cuban, Dominican, Mexican or Mexican American, Puerto Rican, Salvadoran, etc.)

© 2025 Gross RS et al. *JAMA Pediatrics*.

- Middle Eastern or North African
  (For example: Algerian, Egyptian, Iranian, Lebanese, Moroccan, Syrian, etc.)
- Native Hawaiian or other Pacific Islander
  (For example: Chamorro, Fijian, Marshallese, Native Hawaiian, Tongan, etc.)
- White
  (For example: English, European, French, German, Irish, Italian, Polish, etc.)
- None of these fully describe [sname]
- I don't know
- I do not want to answer

Note that [your child] is automatically replaced in the survey with the child's name as entered in the enrollment form when the caregiver is completing the survey. When the respondent is the subject, [your child] is replaced by "you".

If any of the last 3 options were selected, none of the other options were allowed to have been selected. Because participants were permitted to indicate that they belonged to multiple racial/ethnic groups, the counts in Table 1 do not add up to the overall sample size in each age group.

We adjusted for race/ethnicity when estimating risk differences, risk ratios, and odds ratios. Race/ethnicity strata were also used in generating balancing weights. For these analyses, a categorical variable was created for race/ethnicity and mutually exclusive groups were formed using the following logic:

1. If only "Black or African American" was selected, the participant is categorized as *non-Hispanic Black*
2. If only "Middle Eastern or North African" or "White" was selected (but not both), the participant is categorized as *non-Hispanic White*
3. If "Hispanic, Latino, or Spanish" was selected with or without any other selections, the participant is categorized as His*panic*
4. Otherwise, the participant is categorized as *Mixed race/Other/Missing*.

Estimation of risk differences and odds ratios

To estimate risk differences, linear regression with robust standard errors was used.[2] To estimate odds ratios, logistic regression was used. The outcome in each model was the prolonged presence of a given symptom. The predictors included exposure status (infected vs. uninfected), sex assigned at birth (female/intersex vs. male), and race/ethnicity (4-category variable described in previous section).

Balancing weights for LASSO

Balancing weights were used to account for differences between the infected and uninfected populations with respect to select demographic variables.[3] In each age group, participants were divided into 8 strata based on sex assigned at birth (2 groups: female/intersex and male) and race/ethnicity (4 groups: non-Hispanic Black, non-Hispanic White, Hispanic, and Mixed race/Other/Missing). We then calculated the balancing weights as follows: For uninfected participants, the weight was the number of infected participants divided by the number of uninfected participants, and for infected participants, the weight was always 1. This approach ensures that any demographic differences between infected and uninfected participants did not influence the selection of symptoms for the Long COVID research index.

Fitting LASSO for defining Long COVID symptom scores

Least Absolute Shrinkage and Selection Operator (LASSO) was used to identify which of the candidate symptoms (see **Methods**) were most predictive of having been infected.[4] Within each age group, LASSO was performed with balancing weights applied (see above section) to ensure balance with respect to infection status, sex assigned at birth, and race/ethnicity. Predictor variables included candidate symptoms and sex assigned at birth (female/intersex vs. male) and race/ethnicity (see above section). History of infection (infected vs. uninfected) was the outcome. 10-

© 2025 Gross RS et al. *JAMA Pediatrics*.

fold cross validation was performed using the cv.glmnet() function from the glmnet package in R to select the appropriate regularization parameter.[5] The tuning parameter was selected using the 1 standard error rule for infants and toddlers and the mean of the minimum lambda plus 1 standard error in preschool-age children. This procedure was repeated 100 times and the average was taken.

Once the model was fit, we obtained estimated regression coefficients (log odds ratios) for each selected symptom. Scores were calculated by dividing each estimated coefficient by 0.10 and rounding up to the nearest 0.5.[6] This approach was taken to be more inclusive of symptoms selected by LASSO and not excluding those with smaller effects from contributing to the overall research index. Total Long COVID research indices were calculated for each participant by summing the symptom scores for each prolonged symptom reported. An optimal threshold was selected following the procedure described in the following section.

Selection of an optimal Long COVID index threshold

Bootstrapped standard errors from 100 bootstrapped datasets were used to generate the error bars shown in **Figure 2** for infants and toddlers and **Figure 3** for preschool-age children. Different methods were used to define error bars due to the substantially different sample sizes between the two cohorts. The optimal threshold $x_{optimal}$ was defined as the minimum index such that the % of uninfected classified as Long COVID-probable was always within 1 error bar of the next highest index for all $x \geq x_{optimal}$. For preschool-age children, this was equivalent to having either of the 2 symptoms that contributed to the index.

Vaccination status

Vaccination status was defined as the participant's status at the time of first infection for infected participants and at the time of enrollment for uninfected participants. We will refer to this time in this section as the reference date. The following logic was used to categorize vaccination status.

First, we considered whether the participant was eligible for vaccination at their reference date. Participants aged 4 and under were eligible as of June 17, 2022. Participants aged 5-11 were eligible as of October 29, 2021. A participant was considered *Not eligible for vaccination* if the reference date was not more than 14 days after these dates (i.e., adequate time to receive the full vaccination series). Because the reference date for all uninfected participants was after enrollment began in March 2022, every uninfected participant was eligible for vaccination. Second, participants were grouped based on the response to the question "Has [name] received at least one dose of the COVID vaccine".

1. If the answer was No, then the participant is categorized as *Not vaccinated*.
2. If the answer was Yes, then the participant was asked about the number of vaccines received, the type of vaccines, and their vaccination dates.
    a. Participants who completed their full series 14 days before reference date (1 dose if Johnson & Johnson, 2 doses otherwise) were categorized as *Fully vaccinated*.
    b. If the vaccination series was initiated but not completed 14 days before reference date, they were categorized as *Partially vaccinated*.
    c. If the vaccination series was initiated after the reference date, they were categorized as *Not vaccinated*.
    d. If it was not possible to determine vaccination status 14 days before the reference date due to incomplete or inconsistent data, the participant was categorized as *Vaccinated but missing information*.

Time effects on the presence of prolonged symptoms

This analysis was performed within each age group. For each symptom contributing to the Long COVID research index, we fit a logistic regression model where the exposure was time between first infection and enrollment (i.e., time of symptom survey) and the outcome was the binary presence of the prolonged symptom at enrollment, adjusted for child age. Restricted cubic splines were used for time between first infection and enrollment. Then, with

© 2025 Gross RS et al. *JAMA Pediatrics*.

this model fit, we predicted the probability of having that prolonged symptom every day between 0 and 1600 days in the entire population, with one prediction per participant per tie point. Then, at each time point, we took the average probability across the population. This is the age-standardized probability at a given time point. We then plotted this across all time points using a smoothed line. Bootstrapping of the initial population with 500 bootstrapped datasets was used to generate 95% confidence intervals.

eReferences

1. Gross RS, Thaweethai T, Rosenzweig EB, et al. Researching COVID to enhance recovery (RECOVER) pediatric study protocol: Rationale, objectives and design. PLoS One 2024;19(5):e0285635. DOI: 10.1371/journal.pone.0285635.
2. Cheung YB. A Modified Least-Squares Regression Approach to the Estimation of Risk Difference. American journal of epidemiology 2007;166(11):1337-1344. DOI: 10.1093/aje/kwm223.
3. Chattopadhyay A, Hase CH, Zubizarreta JR. Balancing vs modeling approaches to weighting in practice. Statistics in medicine 2020;39(24):3227-3254. DOI: 10.1002/sim.8659.
4. Hastie TJ, Tibshirani RJ, Friedman JH. The elements of statistical learning : data mining, inference, and prediction : [hard]. Second Edition ed. New York: Springer, 2009.
5. Friedman J, Hastie T, Tibshirani R. Regularization Paths for Generalized Linear Models via Coordinate Descent. Journal of Statistical Software 2010;33(1):1--22. DOI: 10.18637/jss.v033.i01.
6. Gagne JJ, Glynn RJ, Avorn J, Levin R, Schneeweiss S. A combined comorbidity score predicted mortality in elderly patients better than existing scores. Journal of Clinical Epidemiology 2011;64(7):749-759. DOI: 10.1016/j.jclinepi.2010.10.004.

© 2025 Gross RS et al. *JAMA Pediatrics*.

eTables

eTable 1. List of sites in RECOVER-Pediatrics

Cohort Type	Hub Name	Enrolling Sites	Location
de novo pediatric RECOVER cohort	Arkansas Children's Research Institute	Arkansas Children's Research Institute	Arkansas
	Arkansas Children's Research Institute	Avera Research Institute	South Dakota
	Arkansas Children's Research Institute	Dartmouth Hitchcock Medical Center	New Hampshire
	Arkansas Children's Research Institute	Kapiolani Medical Center for Women and Children	Hawaii
	Arkansas Children's Research Institute	Medical University of South Carolina	South Carolina
	Arkansas Children's Research Institute	Nemours Children's Health System	Delaware
	Arkansas Children's Research Institute	Northeastern University, Puerto Rico Testsite	Puerto Rico
	Arkansas Children's Research Institute	Pennington Biomedical Research Center	Louisiana
	Arkansas Children's Research Institute	University of Louisville Research Foundation	Kentucky
	Arkansas Children's Research Institute	University of Nebraska Medical Center	Nebraska
	Arkansas Children's Research Institute	University of New Mexico Health Sciences Center	New Mexico
	Arkansas Children's Research Institute	University of Oklahoma Health Sciences Center	Oklahoma
	Arkansas Children's Research Institute	University of Vermont Medical Center	Vermont
	Arkansas Children's Research Institute	West Virginia University	West Virginia
	Children's Hospital of Los Angeles	Children's Hospital of Los Angeles	California
	Columbia University College of Physicians & Surgeons	Columbia University College of Physicians & Surgeons	New York
	Columbia University College of Physicians & Surgeons	Best Healthcare Inc.	New York
	Rutgers Robert Wood Johnson Medical School	Rutgers Robert Wood Johnson Medical School/Robert Wood Johnson Barnabas Health Bristol Myers Squibb Children's Hospital	New Jersey
	Rutgers Robert Wood Johnson Medical School	American Academy of Pediatrics Pediatric Research in Office	National reach

		Settings (PROS) Network	
	Rutgers Robert Wood Johnson Medical School	Children's Mercy Kansas City	Missouri
	Rutgers Robert Wood Johnson Medical School	Connecticut Children's	Connecticut
	Rutgers Robert Wood Johnson Medical School	American Academy of Family Physicians' National Research Network and DARTNet Institute	National reach
	Rutgers Robert Wood Johnson Medical School	Hackensack Meridian Health Joseph M.Sanzari Children's Hospital	New Jersey
	Rutgers Robert Wood Johnson Medical School	The MetroHealth System	Ohio
	Rutgers Robert Wood Johnson Medical School	New York Medical Center, Westchester Medical Center, Maria Fareri Children's Hospital	New York
	Rutgers Robert Wood Johnson Medical School	RWJBH Saint Barnabas Medical Center	New Jersey
	Rutgers Robert Wood Johnson Medical School	Yale School of Medicine	Connecticut
	University of California San Diego/Rady Children's Hospital	University of California San Diego/Rady Children's Hospital	California
	Virginia Commonwealth University	Virginia Commonwealth University	Virginia
	Virginia Commonwealth University	New York University Grossman School of Medicine	New York
	Virginia Commonwealth University	Rhode Island Hospital	Rhode Island

© 2025 Gross RS et al. *JAMA Pediatrics*.

eTable 2. Tier 1 symptom survey questions

(a) Full wording of symptom survey questions and responses

Question	Answer choices for post-acute infected	Answer choices for post-acute uninfected
Now, we are going to ask about any problems or symptoms that [your child]* had. First, we want to know about the problems or symptoms that kept happening for more than four weeks since the pandemic began.<br><br>Did [your child] have any of these problems or symptoms lasting for more than 4 weeks that started or got worse since the COVID pandemic began in March 2020? These are problems or symptoms that kept happening without stopping or kept happening again and again for longer than 4 weeks.	• No<br>• Yes and it started before [my child]'s COVID infection<br>• Yes and it started during or after [my child]'s COVID infection<br>• I don't know	• Yes<br>• No<br>• I don’t know
Now, we are going to ask about any problems or symptoms [your child] has now. Does [your child] have any of these problems or symptoms now?	• Yes<br>• No<br>• I don’t know	

*[your/my child] is automatically replaced in the survey with the child’s name as entered in the enrollment form when the caregiver is completing the survey. A participant is counted as having a prolonged symptom if they answered “Yes” to both questions. Both “Yes” responses were counted for the first question for infected participants. This was done to ensure that the amount of follow-up time was aligned between infected and uninfected participants (i.e., time between beginning of the pandemic and symptom survey) to minimize bias due to differences in follow-up.

(b) Full wording of symptoms as they appear in the symptom survey

**Symptom category**	**Symptom short name**	**Symptom as it appears in the survey**	Asked about infants and toddlers (ages 0-2 years)	Asked about preschool-age children (ages 3-5 years)
General symptoms or problems	Excess sweat	Sweating more than normal	X	X
	Fever	Fever	X	X
	Fussy	Fussy or cranky (crying a lot)	X	X
	Gained weight	Gained weight more than expected	X	X
	Hot cold spells	Hot and cold spells (feeling hot or cold for no reason)		X
	Increased appetite	Wanting to eat more than normal (increased appetite)	X	X
	Increased thirst	Wanting to drink liquids more than normal (increased thirst)	X	X
	Lost height	Lost height or grew less than expected	X	X
	Lost weight	Lost weight or gained less than expected	X	X

© 2025 Gross RS et al. *JAMA Pediatrics*.

	Low Energy	Low energy or not feeling strong enough to do things		X
	Poor Appetite	Not wanting to eat (poor appetite)	X	X
	Sleepy	Feeling sleepy during the day time		X
	Tired after walking	Feeling very tired after walking		X
	Tired all day long	Feeling very tired all day long		X
	Trouble sleeping	Trouble sleeping	X	X
Symptoms or problems in the eyes, ears, nose, and throat	Blurry vision	Trouble seeing or blurry vision		X
	Chapped lips	Chapped lips	X	X
	Dark circles	Dark circles or color under the eyes	X	X
	Dry eyes	Eyes are dry	X	X
	Dry mouth	Very dry mouth		X
	Hearing changed	Change in hearing		X
	Light hurts eyes	Light hurts your eyes		X
	Loss of smell	Loss of smell		X
	Loss of voice	Loss of voice (sounding hoarse)	X	X
	Problem with teeth/gums	Problems with teeth or gums	X	X
	Problems with swallowing	Problems swallowing	X	X
	Red eyes	Eyes look red	X	X
	Smell changed	Change in smell		
	Sore throat	Throat hurts (sore throat)		X
	Stuffy nose	Stuffy nose or runny nose	X	X
	Watery eyes	Eyes are watery	X	X
Symptoms or problems involving the heart and lungs	Barking Cough	Barking cough	X	X
	Breathing pain	Pain when breathing		X
	Dry cough	Dry cough	X	X
	Wet cough	Wet cough	X	X
	Trouble breathing	Trouble breathing (breathing too fast)	X	
	Trouble running	Trouble running		X
	Trouble walking	Trouble walking		X
	Trouble with stairs	Trouble climbing stairs		X
Symptoms or problems involving the belly	Constipation	Trouble pooping/stooling (constipation)	X	X
	Diarrhea	Loose stool (diarrhea)	X	X
	Frequent urination	Peeing more than normal (urination more than normal)		X
	Nausea	Nausea (feeling like you are going to throw up)	X	X
	Stomach pain	Stomach pains/cramps		X
	Urination pain	Pain with peeing (urination)		X
	Vomiting	Throwing up (vomiting)	X	X
Symptoms or problems	Color change in finger/toes	Color changes on the fingers or toes	X	X

© 2025 Gross RS et al. *JAMA Pediatrics*.

involving the skin, hair, and nails	Hair problems	Changes or problems with hair	X	X
	Itchy skin	Itchiness of the skin		X
	Nail problems	Changes or problems with nails	X	X
	Skin color change	Color changes in your skin, such as red, white or purple	X	X
	Skin rash	Skin rash	X	X
Symptoms or problems involving the bones and muscles	Back pain	Pain in the back		X
	Body pain	Body aches or pains		X
	Joint pain	Pains in the joints (like the elbows, knees, ankles)		X
	Muscle pain	Sore muscles or pain in the muscles		X
	Muscle weakness	Muscle weakness	X	X
	Neck pain	Pain in the neck		X
Symptoms or problems involving the brain and nerves	Brain fog	Problems with focusing on things (concentration), sometimes called "brain fog"		X
	Memory problems	Problems with remembering things (memory)		X
	Problems with talking	Problems with talking		X
	Unable to move body	Unable to move part of the body	X	X
Symptoms or problems involving feelings or behavior	Aggressive behavior	Aggressive behavior like hitting, biting or kicking	X	X
	Fear about specific things	Feeling a lot of fear of specific things like spiders or being up high		X
	Fear of other children/adults	Feeling a lot of fear about being with other children or adults		X
	Fear when away from parent	Feeling a lot of fear when being away from parent or caregiver	X	X
	Frequent tantrums	Having a lot of tantrums	X	X
	Holding breath when scared/angry	Holding their breath for a long time when they are afraid or angry	X	X
	Hyperactive	Being hyperactive or much more active than other children		X
	Night terrors	Screaming in fear while asleep, sometimes called night terrors	X	X
	Nightmares	Having nightmares	X	X
	Refusing to follow rules	Refusing to follow rules or doing what they are asked to do		X
	Repeating thoughts after traumatic event	Having repeating memories, dreams, thoughts, or worries after a traumatic event		X
	Rocking back and forth	Rocking the body back and forth or head banging	x	X
	Serious behavior problems	Serious breaking of rules like lying, stealing, starting fights, or bullying		X

© 2025 Gross RS et al. *JAMA Pediatrics*.

eTable 3. Symptom groupings and correlations, calculated among all participants

Symptom group name	Symptom A	Symptom B	0-2y	3-5y
Daytime tiredness/ sleepiness or low energy	*Feeling sleepy during the daytime*	*Low energy or not feeling strong enough to do things*	-	0.579
	Feeling very tired all day long	*Feeling sleepy during the daytime*	-	0.430
	Low energy or not feeling strong enough to do things	*Feeling very tired all day long*	-	0.638
Red or watery eyes	Eyes are watery	Eyes look red	0.629	0.365
Change or loss of smell	*Change in smell*	*Loss of smell*	-	0.706
Trouble with walking, running, or stairs	*Trouble running*	*Trouble climbing stairs*	-	0.608
	Trouble running	*Trouble walking*	-	0.466
	Trouble walking	*Trouble climbing stairs*	-	0.865
Nausea or Vomiting	Nausea (feeling like you are going to throw up)	Throwing up (vomiting)	0.702	0.549
Skin color change (including fingers or toes)	Skin color change	Color change in fingers/toes	0.706	-[2]
Itchy skin or skin rash	*Itchiness of the skin*	Skin rash	-	0.560
Body, muscle, or joint pain	*Sore muscles or pain in the muscles*	*Pains in the joints (like the elbows, knees, ankles)*	-	0.466
	Sore muscles or pain in the muscles	*Body aches or pain*	-	0.135
	Body aches or pains	*Pains in the joints (like the elbows, knees, ankles)*	-	0.340
Back or neck pain	*Pain in the neck*	*Pain in the back*	-	-0.004
Trouble with memory or focusing	*Problems with focusing on things (concentration), sometimes called "brain fog"*	*Problems with remembering things (memory)*	-	0.586
Nightmares or night terrors	Nightmares	Night terrors	0.636	0.582
Hyperactivity, refusing to follow rules, or frequent tantrums	*Refusing to follow rules*	*Hyperactive*	-	0.530
	Frequent tantrums	*Hyperactive*	-	0.376
	Refusing to follow rules	Frequent tantrums	-	0.562
Serious rule breaking or aggressive behaviors	*Rule breaking*	Aggressive behavior	-	0.301
Separation fears, phobias, or fear of others	Fear when away from parent	*Fear of other children/adults*	-	0.447
	Fear when away from parent	*Fear about specific things*	-	0.389
	Fear of other children/adults	*Fear about specific things*	-	0.178

1. Symptoms that are *italicized* are not asked of all infants and toddlers (0-2y). Some symptom groupings are not present in 0-2y because of this; i.e., frequent tantrums are reported on their own in 0-2y but are grouped with hyperactive and refusing to follow rules in 3-5y.
2. Color change in fingers/toes did not occur in any preschool-age children (3-5y).

© 2025 Gross RS et al. *JAMA Pediatrics*.

eTable 4. First COVID Infection history characteristics of infected participants, by age group

Characteristic	Level	Ages 0-2y (Infant/toddler) (n=278)	Ages 3-5y (Preschooler) (n=399)	Overall (n=677)
Reason for COVID Diagnosis	A doctor told them due to symptoms	79/277 (29%)	62/399 (16%)	141/676 (21%)
	Self-diagnosis without doctor	28/277 (10%)	48/399 (12%)	76/676 (11%)
	Test done by doctor or lab	131/277 (47%)	179/399 (45%)	310/676 (46%)
	Home test kit	74/277 (27%)	137/399 (34%)	211/676 (31%)
	Test done at school	1/277 (0%)	3/399 (1%)	4/676 (1%)
	I don't know or I don’t want to answer	1/277 (0%)	6/399 (2%)	7/676 (1%)
	Missing	1	0	1
Type of COVID Test	Antigen test done elsewhere	61/277 (22%)	84/399 (21%)	145/676 (21%)
	Antigen test done at home	70/277 (25%)	137/399 (34%)	207/676 (31%)
	PCR/molecular test	94/277 (34%)	132/399 (33%)	226/676 (33%)
	Blood test	4/277 (1%)	7/399 (2%)	11/676 (2%)
	I don't know or I don’t want to answer	6/277 (2%)	5/399 (1%)	11/676 (2%)
	Missing	1	0	1

© 2025 Gross RS et al. *JAMA Pediatrics*.

eTable 5. Long COVID frequencies, stratified by age group, infection status, and era of infection

	**Infants and toddlers (Ages 0 to 2 years)**			**Preschool-age children (Ages 3 to 5 years)**		
	**Total, N (%)**	**Long COVID-Probable, N (%)**	**At least 1 symptom, N (%)**	**Total, N (%)**	**Long COVID-Probable, N (%)**	**At least 1 symptom, N (%)**
Infected	278	40 (14%)	114 (41%)	399	61 (15%)	179 (45%)
Pre-Omicron[a]	55	11 (20%)	30 (55%)	196	30 (15%)	89 (45%)
Omicron[b]	223	29 (13%)	84 (38%)	203	31 (15%)	90 (44%)
Uninfected	194	5 (3%)	49 (25%)	140	8 (6%)	52 (37%)

[a]Pre-Omicron refers to participants whose first infection was before December 1, 2021.

[b]Omicron refers to participants whose first infection was December 1, 2021 or later.

© 2025 Gross RS et al. *JAMA Pediatrics*.

eTable 6. Correlation between symptoms contributing to the Long COVID research index and those that do not

Infants and toddlers (Ages 0 to 2 years)					Preschool-age children (Ages 3 to 5 years)				
**Symptom contributing to LC index**	**Highest correlated symptom**		**Second highest correlated symptom**		**Symptom contributing to LC index**	**Highest correlated symptom**		**Second highest correlated symptom**	
	**Symptom**	**Correlation coefficient**	**Symptom**	**Correlation coefficient**		**Symptom**	**Correlation coefficient**	**Symptom**	**Correlation coefficient**
Poor appetite	Fever	0.30	Lost height	0.25	Daytime tiredness/sleepiness or low energy	Excess sweat	0.34	Sore throat	0.33
Trouble sleeping	Excess sweat	0.33	Fussy	0.31	Dry cough	Barking cough	0.45	Nausea or vomiting	0.32
Wet cough	Barking cough	0.32	Trouble breathing	0.30					
Dry cough	Trouble breathing	0.34	Barking cough	0.28					
Stuffy nose	Fever	0.32	Fussy	0.30					

© 2025 Gross RS et al. *JAMA Pediatrics*.

eTable 7. Proportion of participants experiencing each of all 75 original prolonged symptoms in each age group

Symptom Category	Grouped Symptom	Symptom	Ages 0 - 2 (Infants/Toddlers) Infected, n/N (%)	Ages 0 - 2 (Infants/Toddlers) Uninfected, n/N (%)	Ages 3 - 5 (Preschool-age children) Infected, n/N (%)	Ages 3 - 5 (Preschool-age children) Uninfected, n/N (%)
General Symptoms	Fever	Fever	9/277 (3.2%)	1/194 (0.5%)	16/399 (4.0%)	1/139 (0.7%)
	Daytime tiredness/sleepiness or low energy	Sleepy			14/398 (3.5%)	1/139 (0.7%)
		Low Energy			13/399 (3.3%)	0/139 (0.0%)
		Tired all day long			9/398 (2.3%)	0/139 (0.0%)
	Trouble sleeping	Trouble sleeping	22/277 (7.9%)	4/194 (2.1%)	28/399 (7.0%)	7/139 (5.0%)
	Fussy	Fussy	21/276 (7.6%)	5/191 (2.6%)	37/399 (9.3%)	7/139 (5.0%)
	Tired after walking	Tired after walking			21/399 (5.3%)	4/139 (2.9%)
	Excess sweat	Excess sweat	10/277 (3.6%)	1/194 (0.5%)	10/398 (2.5%)	3/139 (2.2%)
	Hot cold spells	Hot cold spells			8/396 (2.0%)	1/139 (0.7%)
	Poor Appetite	Poor Appetite	21/277 (7.6%)	3/193 (1.6%)	31/399 (7.8%)	6/139 (4.3%)
	Increased appetite	Increased appetite	1/277 (0.4%)	2/193 (1.0%)	4/399 (1.0%)	5/140 (3.6%)
	Increased thirst	Increased thirst	11/277 (4.0%)	0/193 (0.0%)	20/399 (5.0%)	7/140 (5.0%)
	Lost weight	Lost weight	10/276 (3.6%)	2/194 (1.0%)	15/398 (3.8%)	3/140 (2.1%)
	Gained weight	Gained weight			4/398 (1.0%)	2/140 (1.4%)
	Lost height	Lost height	2/276 (0.7%)	2/194 (1.0%)	1/397 (0.3%)	1/140 (0.7%)
EENT	Watery or red eyes	Red eyes	2/277 (0.7%)	0/194 (0.0%)	9/399 (2.3%)	0/140 (0.0%)
		Watery eyes	5/277 (1.8%)	1/194 (0.5%)	7/399 (1.8%)	3/140 (2.1%)
	Dry eyes	Dry eyes	1/277 (0.4%)	0/194 (0.0%)	2/399 (0.5%)	1/140 (0.7%)
	Dark circles	Dark circles	11/277 (4.0%)	2/194 (1.0%)	22/399 (5.5%)	6/140 (4.3%)
	Blurry vision	Blurry vision			2/394 (0.5%)	0/140 (0.0%)
	Light hurts eyes	Light hurts eyes			9/393 (2.3%)	2/140 (1.4%)
	Hearing changed	Hearing changed			2/393 (0.5%)	0/140 (0.0%)
	Stuffy nose	Stuffy nose	40/277 (14.4%)	13/194 (6.7%)	51/399 (12.8%)	10/140 (7.1%)
	Change or loss in smell or taste	Smell changed			2/386 (0.5%)	1/140 (0.7%)
		Loss of smell			1/387 (0.3%)	0/140 (0.0%)
	Dry mouth	Dry mouth			4/393 (1.0%)	1/140 (0.7%)
	Sore throat	Sore throat			18/396 (4.5%)	1/139 (0.7%)
	Loss of voice	Loss of voice	2/277 (0.7%)	1/194 (0.5%)	2/397 (0.5%)	0/140 (0.0%)
	Problems with swallowing	Problems with swallowing	2/277 (0.7%)	0/194 (0.0%)	2/397 (0.5%)	1/140 (0.7%)
	Problem with teeth/gums	Problem with teeth/gums	2/277 (0.7%)	1/194 (0.5%)	3/397 (0.8%)	1/140 (0.7%)
	Chapped lips	Chapped lips	7/277 (2.5%)	2/194 (1.0%)	17/398 (4.3%)	5/140 (3.6%)
Heart and Lungs	Dry cough	Dry cough	19/276 (6.9%)	3/193 (1.6%)	45/399 (11.3%)	7/140 (5.0%)
	Wet cough	Wet cough	23/276 (8.3%)	4/194 (2.1%)	32/399 (8.0%)	8/140 (5.7%)
	Barking Cough	Barking Cough	7/276 (2.5%)	1/194 (0.5%)	17/398 (4.3%)	2/140 (1.4%)
	Trouble breathing	Trouble breathing	8/276 (2.9%)	1/194 (0.5%)	15/399 (3.8%)	1/140 (0.7%)
	Breathing pain	Breathing pain			3/395 (0.8%)	0/140 (0.0%)
	Trouble with walking, running or stairs	Trouble walking			3/398 (0.8%)	0/140 (0.0%)
		Trouble with stairs	2/104 (1.9%)	0/46 (0.0%)	4/398 (1.0%)	0/140 (0.0%)
		Trouble running	3/104 (2.9%)	0/46 (0.0%)	6/398 (1.5%)	1/140 (0.7%)
Stomach	Stomach pain	Stomach pain			11/396 (2.8%)	4/140 (2.9%)
	Nausea or Vomiting	Nausea	4/274 (1.5%)	1/191 (0.5%)	8/397 (2.0%)	0/140 (0.0%)
		Vomiting	8/277 (2.9%)	2/194 (1.0%)	10/399 (2.5%)	0/140 (0.0%)

© 2025 Gross RS et al. *JAMA Pediatrics*.

Symptom Category	Grouped Symptom	Symptom	Ages 0 - 2 (Infants/Toddlers) Infected, n/N (%)	Ages 0 - 2 (Infants/Toddlers) Uninfected, n/N (%)	Ages 3 - 5 (Preschool-age children) Infected, n/N (%)	Ages 3 - 5 (Preschool-age children) Uninfected, n/N (%)
	Diarrhea	Diarrhea	6/276 (2.2%)	4/194 (2.1%)	12/399 (3.0%)	3/140 (2.1%)
	Constipation	Constipation	7/276 (2.5%)	2/194 (1.0%)	20/399 (5.0%)	4/140 (2.9%)
	Urination pain	Urination pain			2/396 (0.5%)	0/140 (0.0%)
	Frequent urination	Frequent urination			6/398 (1.5%)	2/140 (1.4%)
Skin, Hair, Nails	Itchy skin or skin rash	Skin rash	18/277 (6.5%)	7/193 (3.6%)	15/399 (3.8%)	5/140 (3.6%)
		Itchy skin			24/399 (6.0%)	5/139 (3.6%)
	Nail problems	Nail problems	3/277 (1.1%)	0/194 (0.0%)	2/398 (0.5%)	0/140 (0.0%)
	Hair problems	Hair problems	2/277 (0.7%)	0/194 (0.0%)	1/398 (0.3%)	0/140 (0.0%)
	Change in color of skin, fingers or toes	Skin color change	2/277 (0.7%)	0/194 (0.0%)	2/399 (0.5%)	0/140 (0.0%)
		Color change in finger/toes	1/277 (0.4%)	0/194 (0.0%)		
Muscles and Bones	Muscle weakness	Muscle weakness	4/273 (1.5%)	2/193 (1.0%)	9/396 (2.3%)	0/140 (0.0%)
	Body, muscle, or joint pain	Muscle pain			5/395 (1.3%)	0/140 (0.0%)
		Body pain			9/397 (2.3%)	2/140 (1.4%)
		Joint pain			8/397 (2.0%)	2/140 (1.4%)
	Back or neck pain	Back pain			1/395 (0.3%)	0/140 (0.0%)
		Neck pain			2/394 (0.5%)	0/140 (0.0%)
Nerves and Brain	Trouble with memory or focusing	Memory problems			6/394 (1.5%)	2/139 (1.4%)
		Brain fog			17/391 (4.3%)	3/139 (2.2%)
	Problems with talking	Problems with talking			5/398 (1.3%)	5/140 (3.6%)
Feelings and Behavior	Phobias, Separation Anxiety, or Fear of Others	Fear when away from parent	19/275 (6.9%)	11/191 (5.8%)	41/395 (10.4%)	12/139 (8.6%)
		Fear about specific things	2/104 (1.9%)	4/46 (8.7%)	19/398 (4.8%)	9/139 (6.5%)
		Fear of other children/adults	3/104 (2.9%)	3/46 (6.5%)	11/395 (2.8%)	3/140 (2.1%)
	Hyperactivity, refusing to follow rules, or frequent tantrums	Frequent tantrums	21/276 (7.6%)	13/192 (6.8%)	47/399 (11.8%)	19/140 (13.6%)
		Hyperactive			26/396 (6.6%)	11/140 (7.9%)
		Refusing to follow rules			31/399 (7.8%)	13/140 (9.3%)
	Holding breath when scared/angry	Holding breath when scared/angry	4/276 (1.4%)	2/192 (1.0%)	2/396 (0.5%)	0/139 (0.0%)
	Nightmares or night terrors	Nightmares	6/276 (2.2%)	2/192 (1.0%)	22/399 (5.5%)	7/140 (5.0%)
		Night terrors	10/275 (3.6%)	3/192 (1.6%)	21/399 (5.3%)	4/140 (2.9%)
	Serious rule breaking or aggressive behaviors	Aggressive behavior	11/276 (4.0%)	4/192 (2.1%)	26/399 (6.5%)	12/140 (8.6%)
		Serious behavior problems			6/399 (1.5%)	2/140 (1.4%)
	Rocking back and forth	Rocking back and forth	10/277 (3.6%)	4/194 (2.1%)	8/399 (2.0%)	2/140 (1.4%)
	Repeating thoughts after traumatic event	Repeating thoughts after traumatic event			6/397 (1.5%)	4/139 (2.9%)

© 2025 Gross RS et al. *JAMA Pediatrics*.

eFigures

eFigure 1. Application of RECOVER-Pediatrics analysis cohort inclusion and exclusion criteria

(a)

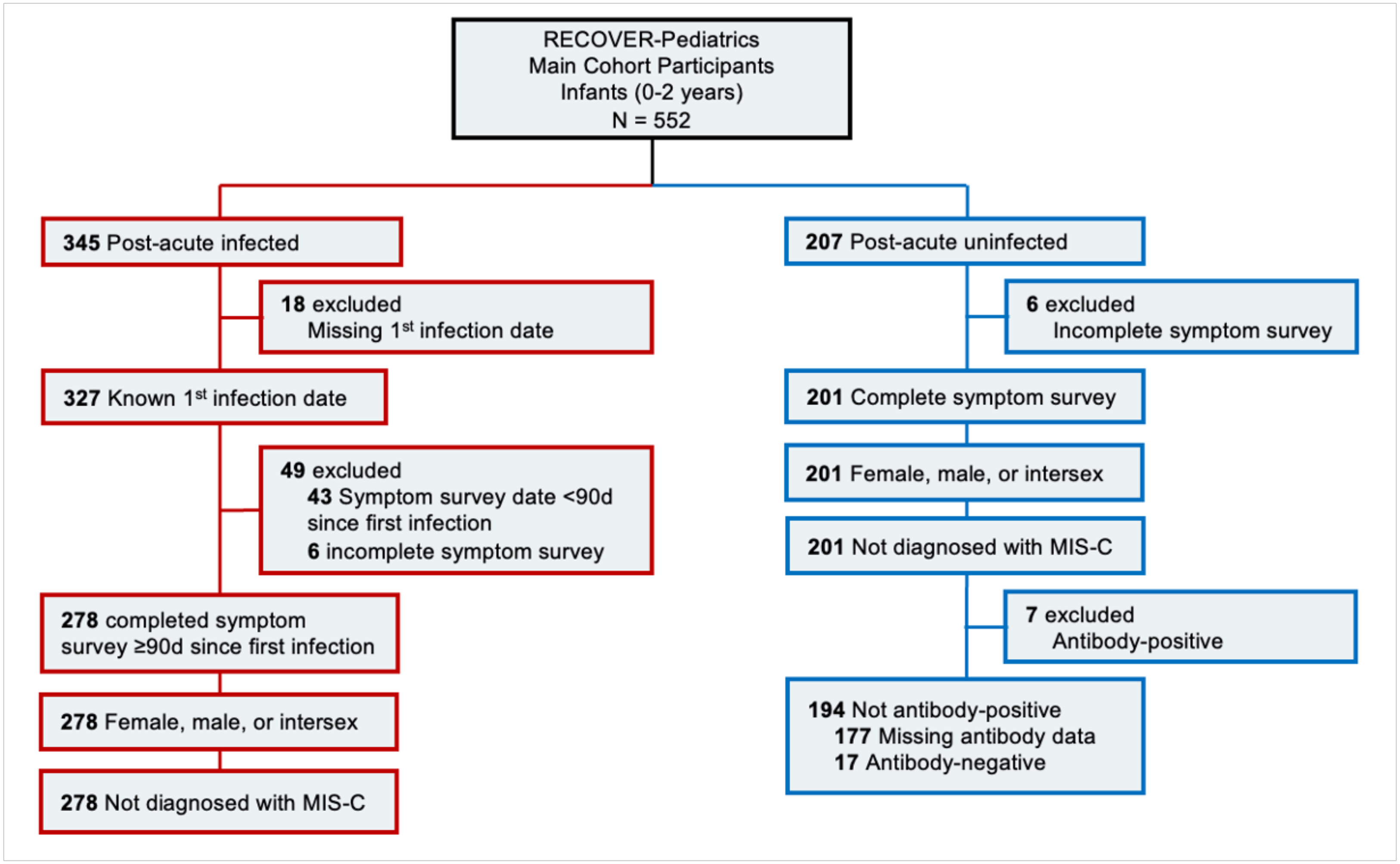

© 2025 Gross RS et al. *JAMA Pediatrics*.

(b)

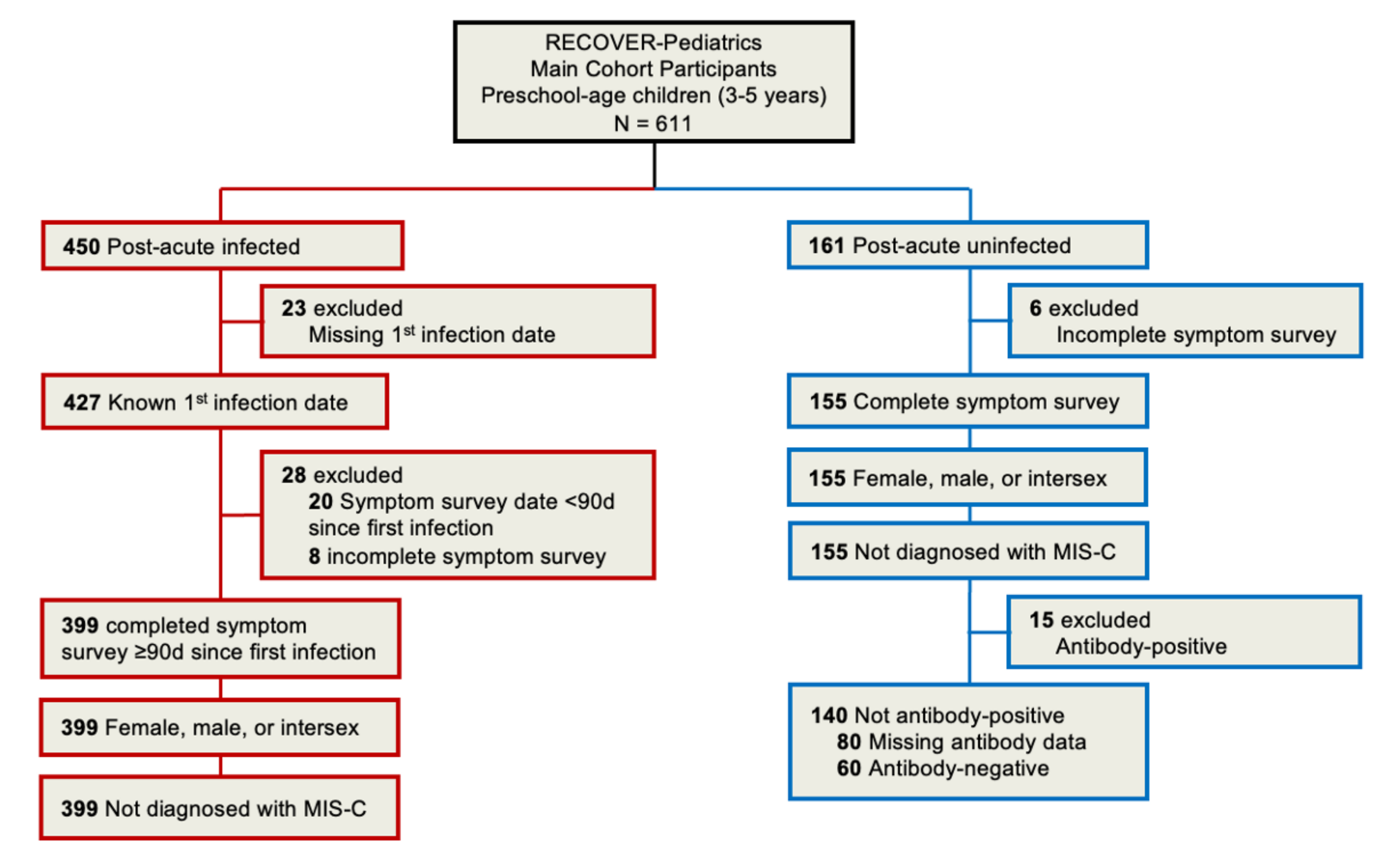

© 2025 Gross RS et al. *JAMA Pediatrics*.

eFigure 2. Participants with each prolonged symptom, adjusted odds ratios, and adjusted risk differences comparing infected vs. uninfected participants

(a) Infants and toddlers (0-2 years)

Symptoms		Infected (n=278)		Uninfected (n=194)		aOR [95% CI]	aRD [95% CI]
General Symptoms	Fever	9	3.2%	1	0.5%		
	Trouble sleeping	22	7.9%	4	2.1%	4.08 (1.52, 14.16)	5.8% (2%, 9.5%)
	Fussy	21	7.6%	5	2.6%	3.09 (1.23, 9.39)	5% (1.1%, 8.9%)
	Excess sweat	10	3.6%	1	0.5%		
	Poor Appetite	21	7.6%	3	1.6%	5.32 (1.79, 22.85)	6.1% (2.5%, 9.7%)
	Increased appetite	1	0.4%	2	1%		
	Increased thirst	11	4%	0	0%		
	Lost weight	10	3.6%	2	1%		
	Gained weight	0	0%	0	0%		
	Lost height	2	0.7%	2	1%		
EENT	Watery or red eyes	5	1.8%	1	0.5%		
	Dry eyes	1	0.4%	0	0%		
	Dark circles	11	4%	2	1%		
	Stuffy nose	40	14.4%	13	6.7%	2.39 (1.27, 4.78)	7.8% (2.3%, 13.4%)
	Loss of voice	2	0.7%	1	0.5%		
	Problems with swallowing	2	0.7%	0	0%		
	Problem with teeth/gums	2	0.7%	1	0.5%		
	Chapped lips	7	2.5%	2	1%		
Heart and Lungs	Dry cough	19	6.9%	3	1.6%	4.71 (1.57, 20.3)	5.3% (1.8%, 8.8%)
	Wet cough	23	8.3%	4	2.1%	4.28 (1.61, 14.79)	6.2% (2.3%, 10%)
	Barking Cough	7	2.5%	1	0.5%		
	Trouble breathing	8	2.9%	1	0.5%		
Stomach	Nausea or Vomiting	8	2.9%	2	1%		
	Diarrhea	6	2.2%	4	2.1%		
	Constipation	7	2.5%	2	1%		
Skin, Hair, Nails	Itchy skin or skin rash	18	6.5%	7	3.6%	1.86 (0.79, 4.87)	2.9% (-1.1%, 6.9%)
	Nail problems	3	1.1%	0	0%		
	Hair problems	2	0.7%	0	0%		
	Change in color of skin, fingers or toes	2	0.7%	0	0%		
Muscles and Bones	Muscle weakness	4	1.5%	2	1%		
Nerves and Brain	Unable to move body	0	0%	0	0%		
Feelings and Behavior	Phobias, Separation Anxiety, or Fear of Others	20	7.2%	15	7.9%	0.91 (0.45, 1.85)	-0.5% (-5.4%, 4.3%)
	Hyperactivity, refusing to follow rules, or frequent tantrums	21	7.6%	13	6.8%	1.16 (0.57, 2.45)	1% (-3.8%, 5.7%)
	Holding breath when scared/angry	4	1.4%	2	1%		
	Nightmares or night terrors	11	4%	3	1.5%		
	Serious rule breaking or aggressive behaviors	11	4%	4	2.1%		
	Rocking back and forth	10	3.6%	4	2.1%		

aOR [95% CI]

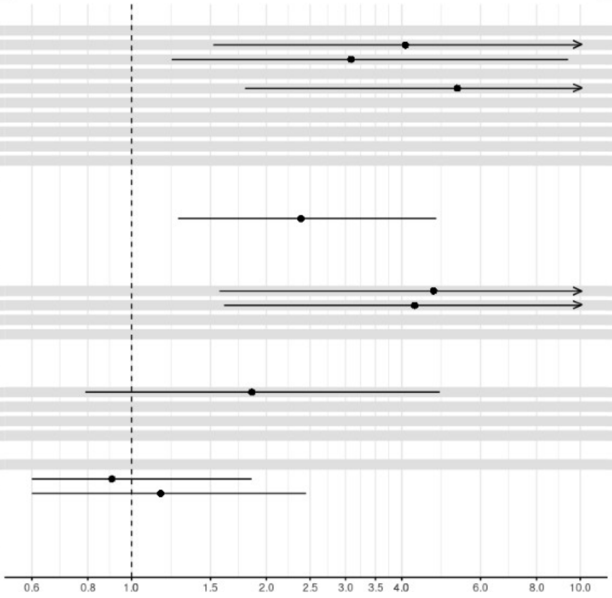

© 2025 Gross RS et al. *JAMA Pediatrics*.

(b) Preschool-age children

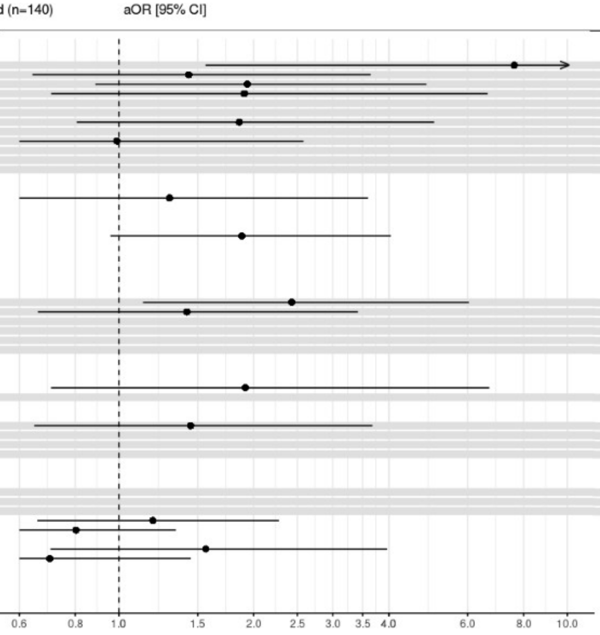

Symptoms		Infected (n=399)		Uninfected (n=140)		aOR [95% CI]	aRD [95% CI]
	Daytime tiredness/sleepiness or low energy	20	5%	1	0.7%	7.62 (1.56, 137.58)	4.4% (1.8%, 7.1%)
	Trouble sleeping	28	7%	7	5%	1.43 (0.64, 3.64)	2% (-2.5%, 6.5%)
	Fussy	37	9.3%	7	5%	1.93 (0.89, 4.86)	4.2% (-0.5%, 8.9%)
	Tired after walking	21	5.3%	4	2.9%	1.91 (0.71, 6.64)	2.5% (-1.1%, 6%)
	Excess sweat	10	2.5%	3	2.2%		
	Hot cold spells	8	2%	1	0.7%		
	Poor Appetite	31	7.8%	6	4.3%	1.86 (0.81, 5.05)	3.4% (-0.9%, 7.7%)
	Increased appetite	4	1%	5	3.6%		
	Increased thirst	20	5%	7	5%	0.99 (0.43, 2.58)	0% (-4.3%, 4.2%)
	Lost weight	15	3.8%	3	2.1%		
	Gained weight	4	1%	2	1.4%		
	Lost height	1	0.3%	1	0.7%		
EENT	Watery or red eyes	13	3.3%	3	2.1%		
	Dry eyes	2	0.5%	1	0.7%		
	Dark circles	22	5.5%	6	4.3%	1.3 (0.54, 3.6)	1.2% (-2.9%, 5.3%)
	Blurry vision	2	0.5%	0	0%		
	Light hurts eyes	9	2.3%	2	1.4%		
	Hearing changed	2	0.5%	0	0%		
	Stuffy nose	51	12.8%	10	7.1%	1.88 (0.96, 4.04)	5.4% (0%, 10.8%)
	Dry mouth	4	1%	1	0.7%		
	Sore throat	18	4.5%	1	0.7%		
	Loss of voice	2	0.5%	0	0%		
	Problems with swallowing	2	0.5%	1	0.7%		
	Problem with teeth/gums	3	0.8%	1	0.7%		
	Chapped lips	17	4.3%	5	3.6%		
Heart and Lungs	Dry cough	45	11.3%	7	5%	2.43 (1.13, 6.03)	6.3% (1.5%, 11%)
	Wet cough	32	8%	8	5.7%	1.42 (0.66, 3.41)	2.1% (-2.6%, 6.8%)
	Barking Cough	17	4.3%	2	1.4%		
	Trouble breathing	15	3.8%	1	0.7%		
	Breathing pain	3	0.8%	0	0%		
	Trouble with walking, running or stairs	7	1.8%	1	0.7%		
Stomach	Stomach pain	11	2.8%	4	2.9%		
	Nausea or Vomiting	13	3.3%	0	0%		
	Diarrhea	12	3%	3	2.1%		
	Constipation	20	5%	4	2.9%	1.92 (0.71, 6.71)	2.4% (-1.2%, 6.1%)
General Symptoms	Fever	16	4%	1	0.7%		
	Urination pain	2	0.5%	0	0%		
	Frequent urination	6	1.5%	2	1.4%		
Skin, Hair, Nails	Itchy skin or skin rash	28	7%	7	5%	1.45 (0.65, 3.68)	2.1% (-2.4%, 6.6%)
	Nail problems	2	0.5%	0	0%		
	Hair problems	1	0.3%	0	0%		
	Change in color of skin, fingers or toes	2	0.5%	0	0%		
Muscles and Bones	Muscle weakness	9	2.3%	0	0%		
	Body, muscle, or joint pain	16	4%	3	2.1%		
	Back or neck pain	3	0.8%	0	0%		
Nerves and Brain	Unable to move body	0	0%	0	0%		
	Trouble with memory or focusing	17	4.3%	4	2.9%		
	Problems with talking	5	1.3%	5	3.6%		
Feelings and Behavior	Phobias, Separation Anxiety, or Fear of Others	49	12.3%	15	10.7%	1.19 (0.66, 2.27)	1.8% (-4.3%, 7.9%)
	Hyperactivity, refusing to follow rules, or frequent tantrums	64	16%	27	19.3%	0.8 (0.49, 1.34)	-3.2% (-10.8%, 4.4%)
	Holding breath when scared/angry	2	0.5%	0	0%		
	Nightmares or night terrors	30	7.5%	7	5%	1.56 (0.7, 3.97)	2.6% (-1.8%, 7%)
	Serious rule breaking or aggressive behaviors	28	7%	13	9.3%	0.7 (0.36, 1.45)	-2.6% (-8.1%, 2.9%)
	Rocking back and forth	8	2%	2	1.4%		
	Repeating thoughts after traumatic event	6	1.5%	4	2.9%		
	Change or loss in smell or taste	2	0.5%	1	0.7%		

Abbreviations: aOR: adjusted odds ratio, aRD: adjusted risk difference.

A symptom was included if at least 5% of infected or uninfected participants in that age group reported experiencing that symptom in a prolonged fashion. aOR and aRD are estimated from models that included infection status as exposure and the presence of each symptom as outcome, with adjustment for sex assigned at birth and race/ethnicity (see eMethods for how race/ethnicity categories were defined.)

© 2025 Gross RS et al. *JAMA Pediatrics*.

eFigure 3. Correlation matrix of Long COVID symptoms among infected Long COVID-probable infants and toddlers and preschool-age children.

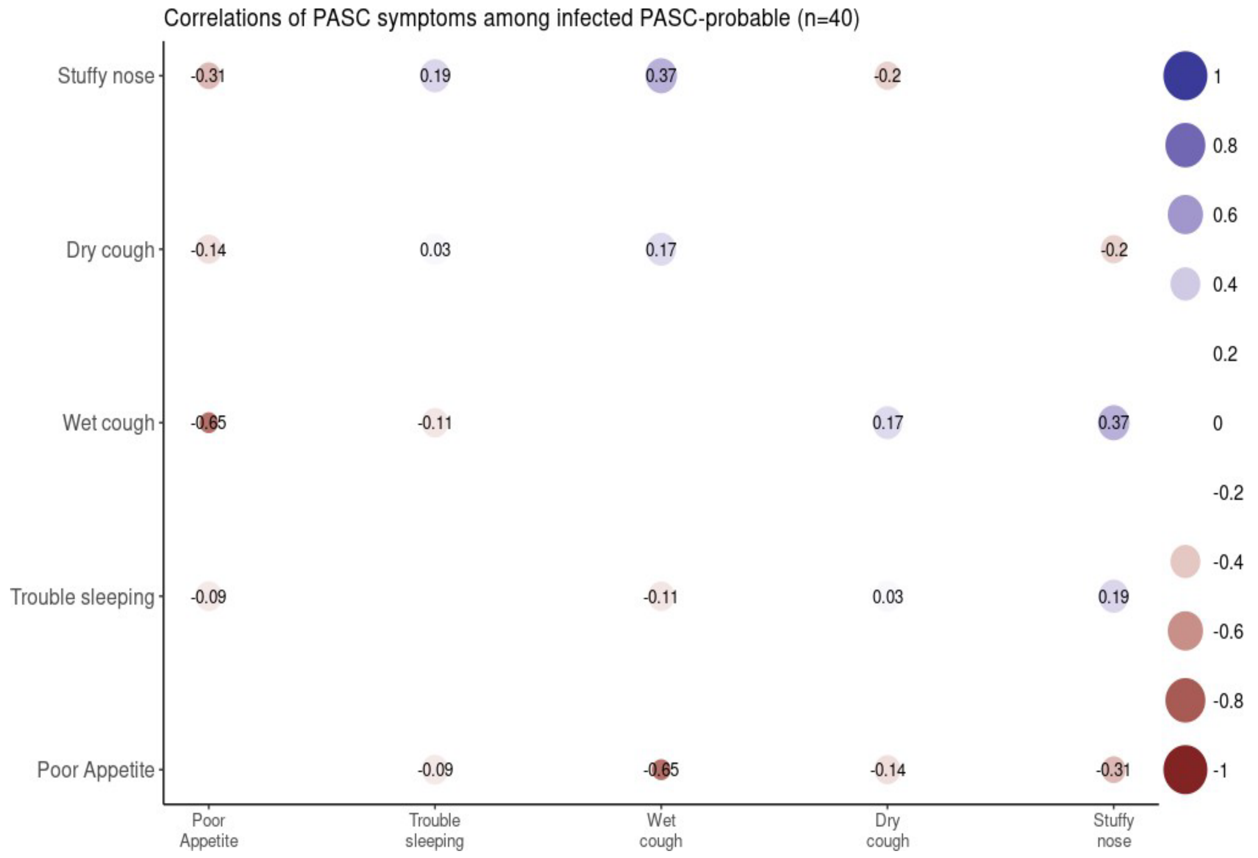

Note: The figure above is for Long COVID-probable infants and toddlers. The correlation between dry cough and daytime tiredness/sleepiness for Long COVID-probable preschool-age children was –0.85.

© 2025 Gross RS et al. *JAMA Pediatrics*.

eFigure 4. Heatmap showing frequency of prolonged symptoms stratified by infection and Long COVID status

Ages 0 – 2 (Infants and Toddlers)			Symptom	Ages 3-5 (Preschool-aged children)		
Uninfected (n=194)	Infected, LC-Unspecified (n=238)	Infected, LC-Probable (n=40)		Infected, LC-Probable (n=61)	Infected, LC-Unspecified (n=338)	Uninfected (n=140)
7%	5%	20%	Hyperactivity, refusing to follow rules, or frequent tantrums	30%	14%	19%
7%	7%	57%	● Stuffy nose	34%	9%	7%
8%	5%	22%	Phobias, Separation Anxiety, or Fear of Others	30%	9%	11%
2%	4%	25%	● Dry cough ●	74%	0%	5%
3%	4%	28%	Fussy	18%	8%	5%
2%	2%	45%	● Wet cough	26%	5%	6%
2%	0%	52%	● Poor Appetite	23%	5%	4%
2%	5%	28%	● Trouble sleeping	18%	5%	5%
4%	5%	12%	Itchy skin or skin rash	15%	6%	5%
2%	3%	8%	Serious rule breaking or aggressive behaviors	10%	7%	9%
2%	1%	20%	Nightmares or night terrors	18%	6%	5%
			Tired after walking	21%	2%	3%
1%	3%	12%	Dark circles	21%	3%	4%
			Daytime tiredness/sleepiness or low energy ●	33%	0%	1%
			Trouble with memory or focusing	15%	2%	3%
0%	3%	8%	Increased thirst	11%	4%	5%
			Sore throat	20%	2%	1%
			Body, muscle, or joint pain	13%	2%	2%
1%	2%	8%	Constipation	8%	4%	3%
1%	1%	10%	Chapped lips	13%	3%	4%
1%	3%	10%	Lost weight	11%	2%	2%
			Stomach pain	8%	2%	3%
1%	1%	12%	Barking Cough	26%	0%	1%
1%	1%	15%	Fever	16%	2%	1%
1%	0%	18%	Trouble breathing	20%	1%	1%
2%	2%	5%	Diarrhea	10%	2%	2%
1%	1%	18%	Excess sweat	10%	1%	2%
2%	3%	8%	Rocking back and forth	3%	2%	1%
1%	1%	12%	Nausea or Vomiting	16%	1%	0%
1%	1%	8%	Watery or red eyes	11%	2%	2%
			Light hurts eyes	7%	1%	1%
			Problems with talking	3%	1%	4%
			Repeating thoughts after traumatic event	3%	1%	3%
			Hot cold spells	7%	1%	1%
			Trouble with walking, running or stairs	10%	0%	1%
			Frequent urination	7%	1%	1%
1%	1%	2%	Muscle weakness	8%	1%	0%
1%	0%	0%	Increased appetite	3%	1%	4%
			Dry mouth	5%	0%	1%
1%	2%	0%	Holding breath when scared/angry	0%	1%	0%
1%	0%	2%	Problem with teeth/gums	3%	0%	1%
1%	0%	5%	Lost height	0%	0%	1%
0%	0%	0%	Gained weight	3%	1%	1%
			Breathing pain	5%	0%	0%
			Change or loss in smell or taste	2%	0%	1%
			Back or neck pain	0%	1%	0%
0%	0%	5%	Problems with swallowing	3%	0%	1%
0%	0%	5%	Nail problems	0%	1%	0%
1%	0%	2%	Loss of voice	2%	0%	0%
0%	0%	2%	Change in color of skin, fingers or toes	3%	0%	0%
0%	0%	2%	Dry eyes	2%	0%	1%
			Urination pain	2%	0%	0%
			Blurry vision	2%	0%	0%
			Hearing changed	0%	1%	0%
0%	0%	5%	Hair problems	2%	0%	0%
0%	0%	0%	Unable to move body	0%	0%	0%

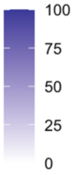

Symptoms, sorted from most to least common in the study population overall, are in the center column. The left columns correspond to infants and toddlers in 3 groups: uninfected, infected and not meeting the Long COVID research index threshold (infected, Long COVID-unspecified), and infected and meeting the Long COVID research index threshold (infected, Long COVID-probable). The columns on the right correspond to preschool-age children with the columns in reverse order. Percentages are not shown for symptoms that are not asked in a given age group. Frequency of each prolonged symptom is indicated by shading, from 0% to 100%. A circle next to a symptom indicates that it contributes to the research index in the given age group.

© 2025 Gross RS et al. *JAMA Pediatrics*.

eFigure 5. Number of systems affected among infected Long COVID-probable participants

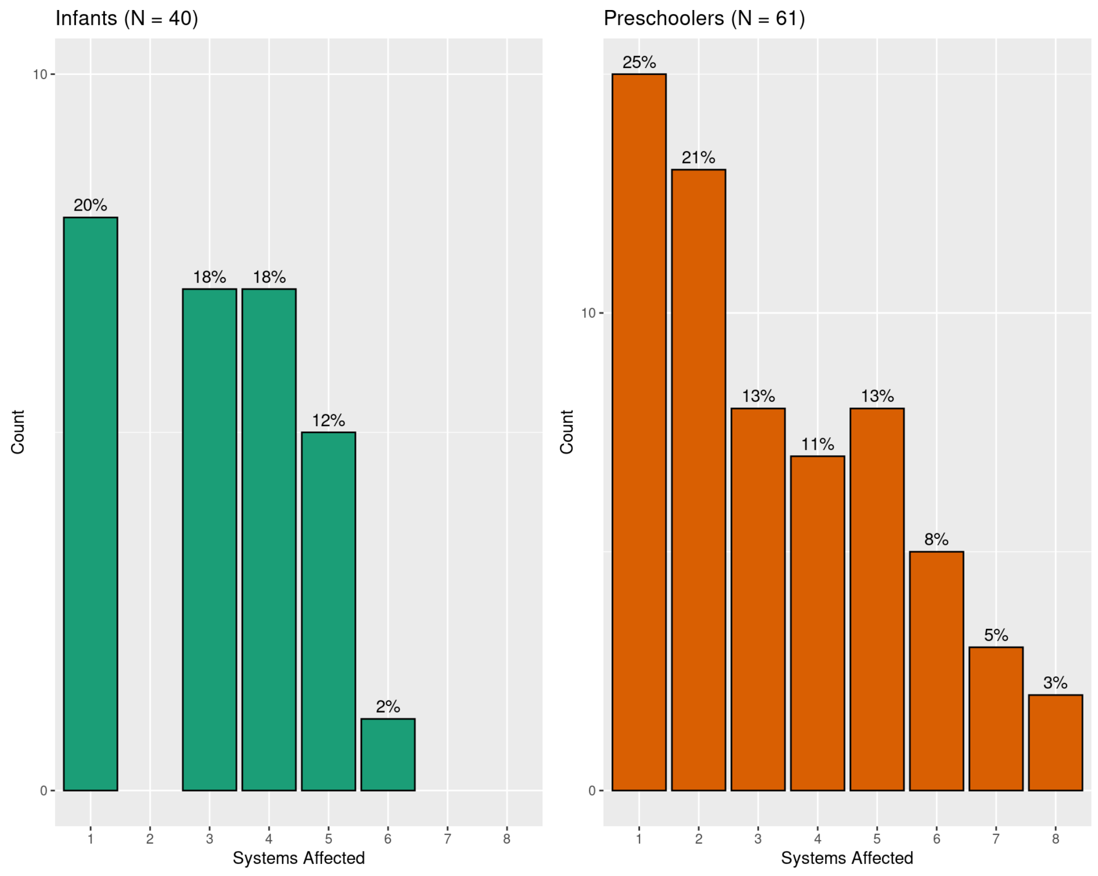

© 2025 Gross RS et al. *JAMA Pediatrics*.

eFigure 6. Estimated probability of the presence of each prolonged symptom contributing to the Long COVID research indices, after age standardization.

a) Infants and toddlers

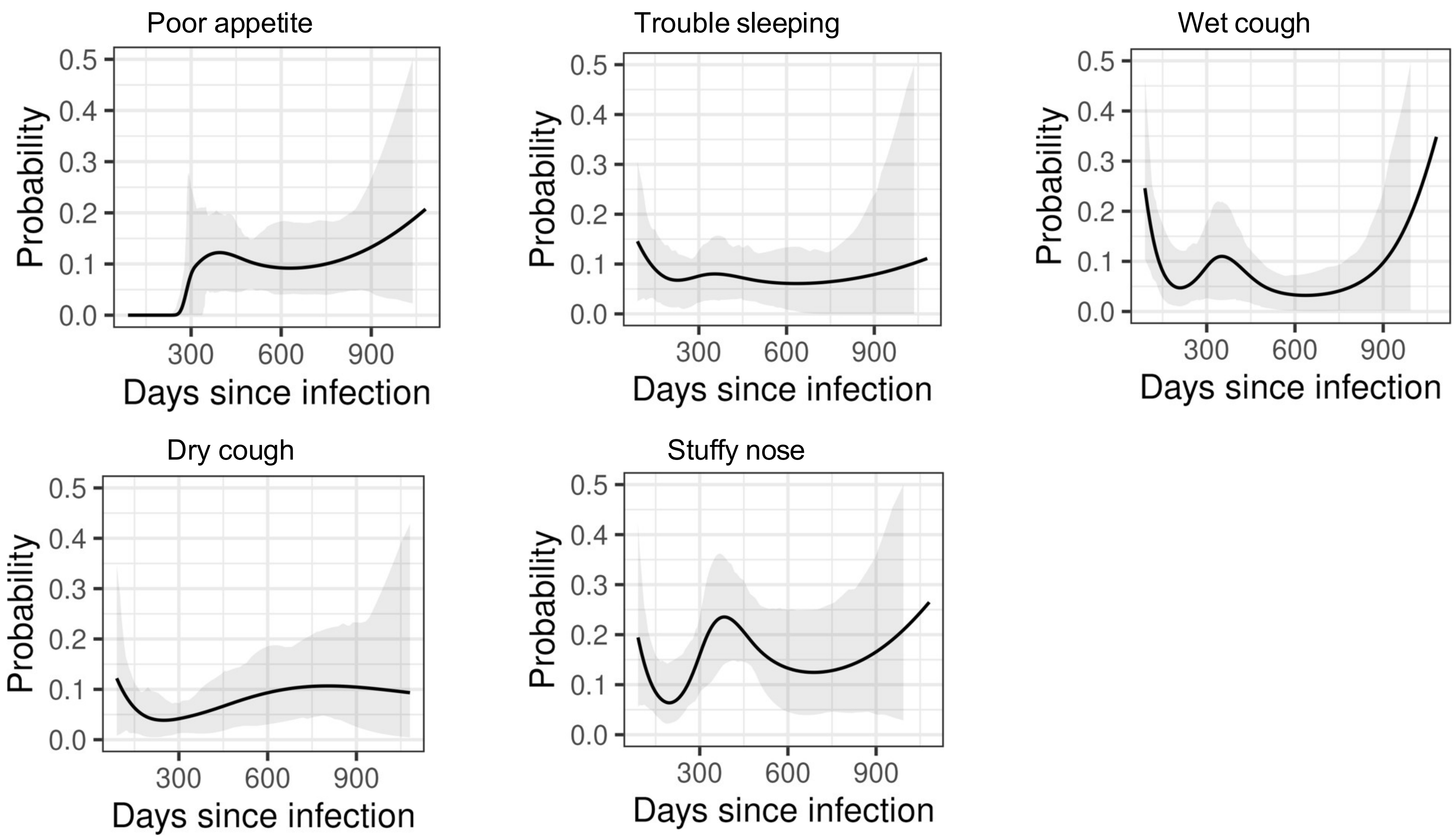

© 2025 Gross RS et al. *JAMA Pediatrics*.

b) Preschool-age children

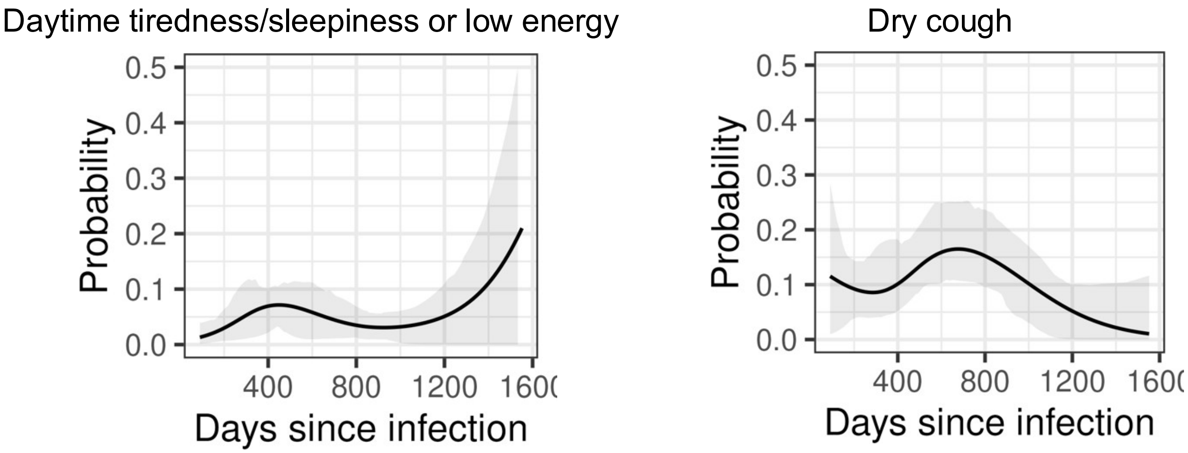

Note that these figures do not represent individual participant trajectories; rather, they represent the association between days since infection and the probability of each symptom. Each participant has contributed one data point which has been smoothed over. See Methods for additional details.

© 2025 Gross RS et al. *JAMA Pediatrics*.
