# Supplementary material for: Characterizing Long COVID Symptoms During Early Childhood
Source: JAMA Pediatr. 2025 May 27;179(7):781–92. doi: 10.1001/jamapediatrics.2025.1066 (PMC12117493; doi:10.1001/jamapediatrics.2025.1066)
Supplement: Supplement 2. — Nonauthor Collaborators. RECOVER-Pediatrics Consortium. [file jamapediatr-e251066-s002.pdf]

Supplemental Online Content: Nonauthor Collaborators
*First name, last name, and suffix (if applicable) are required and will appear in PubMed.

*Group Name(s): RECOVER-Pediatrics Consortium							
***First Name and Middle Initial(s)**	***Last Name**	***Suffix (eg, Jr, III)**	Academic Degrees	Institution	Location (city, state/province, country)	Role or Contribution, eg, chair, principal investigator	Group (if more than 1 Group listed in the byline) and/or Subgroup (eg, Steering Committee)
Paul M.	Darden			Arkansas Children's Research Institute	Little Rock, AR, USA	Sub-Investigator	RECOVER-Pediatric
Lexie	Dixon			Arkansas Children's Research Institute	Little Rock, AR, USA	Site Coordinator	RECOVER-Pediatric
Danielle N.	Evans			Arkansas Children's Research Institute	Little Rock, AR, USA	RECOVER Program Director	RECOVER-Pediatric
Connor	Garbe			Arkansas Children's Research Institute	Little Rock, AR, USA	Data manager	RECOVER-Pediatric
Laura	Hobart-Porter			Arkansas Children's Research Institute	Little Rock, AR, USA	Sub-Investigator	RECOVER-Pediatric
Lee	Howard			Arkansas Children's Research Institute	Little Rock, AR, USA	Research Coordinator	RECOVER-Pediatric
Kathy	Hummel			Arkansas Children's Research Institute	Little Rock, AR, USA	Hub Coordinator	RECOVER-Pediatric
Hannah	Krehbiel			Arkansas Children's Research Institute	Little Rock, AR, USA	Research Assistant	RECOVER-Pediatric
Haley	Spradlin			Arkansas Children's Research Institute	Little Rock, AR, USA	Research Coordinator	RECOVER-Pediatric
Phaedra	Yount			Arkansas Children's Research Institute	Little Rock, AR, USA	Administrative Contact	RECOVER-Pediatric
Grace	Adam		BA	Avera Research Institute	Sioux Falls, SD, USA	Study Coordinator	RECOVER-Pediatric
Jyoti	Angal		PhD	Avera Research Institute	Sioux Falls, SD, USA	Co-Investigator	RECOVER-Pediatric
Maria	Barber		DO	Avera Research Institute	Sioux Falls, SD, USA	Co-Investigator	RECOVER-Pediatric
Katelynne	Clark		BS	Avera Research Institute	Sioux Falls, SD, USA	Study Coordinator	RECOVER-Pediatric
Clayton	Dos Reis		MS	Avera Research Institute	Sioux Falls, SD, USA	Study Coordinator	RECOVER-Pediatric
Mandy	Freesemann		MS	Avera Research Institute	Sioux Falls, SD, USA	Study Coordinator	RECOVER-Pediatric
Christa	Friedrich		MS	Avera Research Institute	Sioux Falls, SD, USA	Study Coordinator	RECOVER-Pediatric
Christine	Hockett		PhD	Avera Research Institute	Sioux Falls, SD, USA	Co-Investigator	RECOVER-Pediatric

Supplemental Online Content: Nonauthor Collaborators
*First name, last name, and suffix (if applicable) are required and will appear in PubMed.

*First Name and Middle Initial(s)	*Last Name	*Suffix (eg, Jr, III)	Academic Degrees	Institution	Location (city, state/province, country)	Role or Contribution, eg, chair, principal investigator	Group (if more than 1 Group listed in the byline) and/or Subgroup (eg, Steering Committee)
Rachel	Johannsen		BS	Avera Research Institute	Sioux Falls, SD, USA	Regulatory Coordinator	RECOVER-Pediatric
Emily	Johnson-Vonk		BS	Avera Research Institute	Sioux Falls, SD, USA	Data Analyst	RECOVER-Pediatric
Cassidy	Kaiser		BSN	Avera Research Institute	Sioux Falls, SD, USA	Study Coordinator, RN	RECOVER-Pediatric
Alexa	Kruse		BS	Avera Research Institute	Sioux Falls, SD, USA	Study Coordinator	RECOVER-Pediatric
Jennifer	Lang		BS	Avera Research Institute	Sioux Falls, SD, USA	Study Coordinator	RECOVER-Pediatric
Peter	Lim		MD	Avera Research Institute	Sioux Falls, SD, USA	Co-Investigator	RECOVER-Pediatric
Meggie	McCoy		BS	Avera Research Institute	Sioux Falls, SD, USA	Lead Coordinator	RECOVER-Pediatric
Lorie	Miller		RN	Avera Research Institute	Sioux Falls, SD, USA	Study Coordinator, RN	RECOVER-Pediatric
Shelby	Petereit (Cerkovnik)		MS	Avera Research Institute	Sioux Falls, SD, USA	Study Coordinator	RECOVER-Pediatric
Jaime	RiChard (Werpy)		AS	Avera Research Institute	Sioux Falls, SD, USA	Research Assistant	RECOVER-Pediatric
Jessica	Seiler		BS	Avera Research Institute	Sioux Falls, SD, USA	Study Coordinator	RECOVER-Pediatric
Bret	Sundleaf		BS	Avera Research Institute	Sioux Falls, SD, USA	Research Assistant	RECOVER-Pediatric
Joshua	Svendsen		PhD	Avera Research Institute	Sioux Falls, SD, USA	Regulatory Coordinator	RECOVER-Pediatric
Billy	Trosper		MS	Avera Research Institute	Sioux Falls, SD, USA	Study Coordinator	RECOVER-Pediatric
Olivia	Vermeulen		MS	Avera Research Institute	Sioux Falls, SD, USA	Study Coordinator	RECOVER-Pediatric
Scott	Young		MS	Avera Research Institute	Sioux Falls, SD, USA	Study Coordinator	RECOVER-Pediatric
Sean	Dabney			Dartmouth Hitchcock Medical Center	Lebanon, NH, USA	Coordinator	RECOVER-Pediatric
Marie-Christine	Fahrner			Dartmouth Hitchcock Medical Center	Lebanon, NH, USA	RN	RECOVER-Pediatric
Torrey	Gallagher			Dartmouth Hitchcock Medical Center	Lebanon, NH, USA	Lab Manager	RECOVER-Pediatric

Supplemental Online Content: Nonauthor Collaborators
*First name, last name, and suffix (if applicable) are required and will appear in PubMed.

***First Name and Middle Initial(s)**	***Last Name**	***Suffix (eg, Jr, III)**	Academic Degrees	Institution	Location (city, state/province, country)	Role or Contribution, eg, chair, principal investigator	Group (if more than 1 Group listed in the byline) and/or Subgroup (eg, Steering Committee)
Karilyn	Martini			Dartmouth Hitchcock Medical Center	Lebanon, NH, USA	RN	RECOVER-Pediatric
Mary	McNally			Dartmouth Hitchcock Medical Center	Lebanon, NH, USA	Coordinator	RECOVER-Pediatric
Sarah	Vivensi Stiverson			Dartmouth Hitchcock Medical Center	Lebanon, NH, USA	CRC	RECOVER-Pediatric
Venkataraman	Balaraman			Kapiolani Medical Center for Women and Children	Honolulu, HI, USA	Sub-Investigator	RECOVER-Pediatric
JoAnn	Cheung			Kapiolani Medical Center for Women and Children	Honolulu, HI, USA	Coordinator	RECOVER-Pediatric
Travis K.F.	Hong			Kapiolani Medical Center for Women and Children	Honolulu, HI, USA	Sub-Investigator	RECOVER-Pediatric
Shanelle	Kalua			Kapiolani Medical Center for Women and Children	Honolulu, HI, USA	Lab Tech	RECOVER-Pediatric
Evan	Minami			Kapiolani Medical Center for Women and Children	Honolulu, HI, USA	RA	RECOVER-Pediatric
Andrea	Siu			Kapiolani Medical Center for Women and Children	Honolulu, HI, USA	Regulatory Coordinator	RECOVER-Pediatric
Micah	Tong			Kapiolani Medical Center for Women and Children	Honolulu, HI, USA	Coordinator	RECOVER-Pediatric
Marina	Dantas			Medical University of South Carolina	Charleston, SC, USA	Coordinator	RECOVER-Pediatric
Tyler	Kasmarcak			Medical University of South Carolina	Charleston, SC, USA	Coordinator	RECOVER-Pediatric
Kreighton	Milks			Medical University of South Carolina	Charleston, SC, USA	Coordinator	RECOVER-Pediatric
Chijoke	Ikomi			Nemours Children's Health System	Wilmington, DE, USA	Sub-Investigator	RECOVER-Pediatric
Marisa	Meyer			Nemours Children's Health System	Wilmington, DE, USA	Sub-Investigator	RECOVER-Pediatric

Supplemental Online Content: Nonauthor Collaborators
*First name, last name, and suffix (if applicable) are required and will appear in PubMed.

***First Name and Middle Initial(s)**	***Last Name**	***Suffix (eg, Jr, III)**	Academic Degrees	Institution	Location (city, state/province, country)	Role or Contribution, eg, chair, principal investigator	Group (if more than 1 Group listed in the byline) and/or Subgroup (eg, Steering Committee)
Connie	Nguyen			Nemours Children's Health System	Wilmington, DE, USA	Coordinator	RECOVER-Pediatric
Gwen	Pellicciotti			Nemours Children's Health System	Wilmington, DE, USA	Coordinator	RECOVER-Pediatric
Thao-Ly	Phan			Nemours Children's Health System	Wilmington, DE, USA	Sub-Investigator	RECOVER-Pediatric
Karen	Ravin			Nemours Children's Health System	Wilmington, DE, USA	Sub-Investigator	RECOVER-Pediatric
Victoria	Reynolds			Nemours Children's Health System	Wilmington, DE, USA	Coordinator	RECOVER-Pediatric
Abigail	Strang			Nemours Children's Health System	Wilmington, DE, USA	Sub-Investigator	RECOVER-Pediatric
Deepika	Thacker			Nemours Children's Health System	Wilmington, DE, USA	Sub-Investigator	RECOVER-Pediatric
Dan	Eckrich			Nemours Children's Hospital Delaware	Wilmington, DE, USA	Informatio. Systems Architect	RECOVER-Pediatric
Annabelle	Goetter			Nemours Children's Hospital Delaware	Wilmington, DE, USA	Research Coordinator	RECOVER-Pediatric
Cheyenne	Katz			Nemours Children's Hospital Delaware	Wilmington, DE, USA	Coordinator	RECOVER-Pediatric
Karen	Kowal			Nemours Children's Hospital Delaware	Wilmington, DE, USA	Lead Coordinator	RECOVER-Pediatric
Carol	McDevitt			Nemours Children's Hospital Delaware	Wilmington, DE, USA	Research Assistant	RECOVER-Pediatric
Genesis	Agosto Roman			Northeastern University, Puerto Rico Testsite for Exploring Contamination		Research Nurse	RECOVER-Pediatric
Akram	Alshawabkeh			Northeastern University, Puerto Rico Testsite for Exploring Contamination		Co-Investigator	RECOVER-Pediatric

Supplemental Online Content: Nonauthor Collaborators
*First name, last name, and suffix (if applicable) are required and will appear in PubMed.

***First Name and Middle Initial(s)**	***Last Name**	***Suffix (eg, Jr, III)**	Academic Degrees	Institution	Location (city, state/province, country)	Role or Contribution, eg, chair, principal investigator	Group (if more than 1 Group listed in the byline) and/or Subgroup (eg, Steering Committee)
Ishwara	Ayala Ortiz			Northeastern University, Puerto Rico Testsite for Exploring Contamination		Data Manager	RECOVER-Pediatric
Virginia	Casey			Northeastern University, Puerto Rico Testsite for Exploring Contamination		Regulatory Coordinator	RECOVER-Pediatric
Jose	Cordero			Northeastern University, Puerto Rico Testsite for Exploring Contamination		Consultant	RECOVER-Pediatric
Jocelyn	De Jesus			Northeastern University, Puerto Rico Testsite for Exploring Contamination		RN	RECOVER-Pediatric
Chrystal M.	Galan Rivera			Northeastern University, Puerto Rico Testsite for Exploring Contamination		Recruitment Coordinator	RECOVER-Pediatric
Gredia	Huerta-Montanez			Northeastern University, Puerto Rico Testsite for Exploring Contamination		Pediatrician	RECOVER-Pediatric
Nilda	Otero			Northeastern University, Puerto Rico Testsite for Exploring Contamination		RN	RECOVER-Pediatric
Mayra	Rivera Robles			Northeastern University, Puerto Rico Testsite for Exploring Contamination		RN	RECOVER-Pediatric
Priscilla	Roman			Northeastern University, Puerto Rico Testsite for Exploring Contamination		Lab Tech	RECOVER-Pediatric
Genesis	Roman			Northeastern University, Puerto Rico Testsite for Exploring Contamination		RN	RECOVER-Pediatric

Supplemental Online Content: Nonauthor Collaborators
*First name, last name, and suffix (if applicable) are required and will appear in PubMed.

***First Name and Middle Initial(s)**	***Last Name**	***Suffix (eg, Jr, III)**	Academic Degrees	Institution	Location (city, state/province, country)	Role or Contribution, eg, chair, principal investigator	Group (if more than 1 Group listed in the byline) and/or Subgroup (eg, Steering Committee)
Zaira	Rosario-Pabon			Northeastern University, Puerto Rico Testsite for Exploring Contamination		Research Director	RECOVER-Pediatric
Xiodenis	Santiago			Northeastern University, Puerto Rico Testsite for Exploring Contamination		Lab Tech	RECOVER-Pediatric
Carmen	Velez-Vega			Northeastern University, Puerto Rico Testsite for Exploring Contamination		Consultant	RECOVER-Pediatric
Carlos	Vergara			Northeastern University, Puerto Rico Testsite for Exploring Contamination		MS	RECOVER-Pediatric
Baylea	Albarado			Pennington Biomedical Research Center	Baton Rouge, LA, USA	Coordinator	RECOVER-Pediatric
Tracey	Allen			Pennington Biomedical Research Center	Baton Rouge, LA, USA	Research Nurse	RECOVER-Pediatric
Allison	Attuso			Pennington Biomedical Research Center	Baton Rouge, LA, USA	Coordinator	RECOVER-Pediatric
Taylor	Ayers			Pennington Biomedical Research Center	Baton Rouge, LA, USA	Coordinator	RECOVER-Pediatric
Emily	Bebler			Pennington Biomedical Research Center	Baton Rouge, LA, USA	Lab Personnel	RECOVER-Pediatric
Grace	Bella			Pennington Biomedical Research Center	Baton Rouge, LA, USA	Recruitment	RECOVER-Pediatric
Alexa	Bennett			Pennington Biomedical Research Center	Baton Rouge, LA, USA	Research Dietitian	RECOVER-Pediatric
John	Brown			Pennington Biomedical Research Center	Baton Rouge, LA, USA	Coordinator	RECOVER-Pediatric
Alison	Carville			Pennington Biomedical Research Center	Baton Rouge, LA, USA	Recruitment	RECOVER-Pediatric

*First name, last name, and suffix (if applicable) are required and will appear in PubMed.

***First Name and Middle Initial(s)**	***Last Name**	***Suffix (eg, Jr, III)**	Academic Degrees	Institution	Location (city, state/province, country)	Role or Contribution, eg, chair, principal investigator	Group (if more than 1 Group listed in the byline) and/or Subgroup (eg, Steering Committee)
Sydnie	Darby			Pennington Biomedical Research Center	Baton Rouge, LA, USA	Coordinator	RECOVER-Pediatric
Kara	Devall			Pennington Biomedical Research Center	Baton Rouge, LA, USA	Coordinator	RECOVER-Pediatric
Amber	Dragg			Pennington Biomedical Research Center	Baton Rouge, LA, USA	Research Dietitian	RECOVER-Pediatric
Angela	Elderedge			Pennington Biomedical Research Center	Baton Rouge, LA, USA	Coordinator	RECOVER-Pediatric
Elisabeth	Fontenot			Pennington Biomedical Research Center	Baton Rouge, LA, USA	Coordinator	RECOVER-Pediatric
Greta	Fry			Pennington Biomedical Research Center	Baton Rouge, LA, USA	Research LPN	RECOVER-Pediatric
Bethany	Gildersleeve			Pennington Biomedical Research Center	Baton Rouge, LA, USA	Program Manager	RECOVER-Pediatric
Sara	Goff			Pennington Biomedical Research Center	Baton Rouge, LA, USA	Research RN	RECOVER-Pediatric
Lauren	Harrington			Pennington Biomedical Research Center	Baton Rouge, LA, USA	Research RN	RECOVER-Pediatric
Lisa	Jones			Pennington Biomedical Research Center	Baton Rouge, LA, USA	Lab Personnel	RECOVER-Pediatric
Victoria	Kaiser			Pennington Biomedical Research Center	Baton Rouge, LA, USA	Coordinator	RECOVER-Pediatric
Yejee	Lee			Pennington Biomedical Research Center	Baton Rouge, LA, USA	Coordinator	RECOVER-Pediatric
Stephen	Lee			Pennington Biomedical Research Center	Baton Rouge, LA, USA	Lab Manager	RECOVER-Pediatric
Erin	LeJeune			Pennington Biomedical Research Center	Baton Rouge, LA, USA	Coordinator	RECOVER-Pediatric
Robert	Leonhard			Pennington Biomedical Research Center	Baton Rouge, LA, USA	Coordinator	RECOVER-Pediatric

*First name, last name, and suffix (if applicable) are required and will appear in PubMed.

***First Name and Middle Initial(s)**	***Last Name**	***Suffix (eg, Jr, III)**	Academic Degrees	Institution	Location (city, state/province, country)	Role or Contribution, eg, chair, principal investigator	Group (if more than 1 Group listed in the byline) and/or Subgroup (eg, Steering Committee)
Jennifer	Levatino			Pennington Biomedical Research Center	Baton Rouge, LA, USA	Research RN	RECOVER-Pediatric
Donald	Lewis			Pennington Biomedical Research Center	Baton Rouge, LA, USA	Lab Personnel	RECOVER-Pediatric
Angrielle	Lloyd			Pennington Biomedical Research Center	Baton Rouge, LA, USA	Nurse Practitioner	RECOVER-Pediatric
Ron	Monce			Pennington Biomedical Research Center	Baton Rouge, LA, USA	Physician Assistant	RECOVER-Pediatric
Susannah	Munro			Pennington Biomedical Research Center	Baton Rouge, LA, USA	Coordinator	RECOVER-Pediatric
Meghan	Phillips			Pennington Biomedical Research Center	Baton Rouge, LA, USA	Coordinator	RECOVER-Pediatric
Blair	Pucheu			Pennington Biomedical Research Center	Baton Rouge, LA, USA	Coordinator	RECOVER-Pediatric
Emily	Rachal			Pennington Biomedical Research Center	Baton Rouge, LA, USA	Coordinator	RECOVER-Pediatric
Jennifer	Rood			Pennington Biomedical Research Center	Baton Rouge, LA, USA	Lab Director	RECOVER-Pediatric
Stacey	Roussel			Pennington Biomedical Research Center	Baton Rouge, LA, USA	Lab Manager	RECOVER-Pediatric
Renee	Rumsey			Pennington Biomedical Research Center	Baton Rouge, LA, USA	Coordinator	RECOVER-Pediatric
Connor	Sanford			Pennington Biomedical Research Center	Baton Rouge, LA, USA	Lab Personnel	RECOVER-Pediatric
Monica	Santos			Pennington Biomedical Research Center	Baton Rouge, LA, USA	Clinical Chem/Lab Tech	RECOVER-Pediatric
Aryelle	Stafford			Pennington Biomedical Research Center	Baton Rouge, LA, USA	Marketing	RECOVER-Pediatric
Amy	Thomassie			Pennington Biomedical Research Center	Baton Rouge, LA, USA	Program Manager	RECOVER-Pediatric

Supplemental Online Content: Nonauthor Collaborators
*First name, last name, and suffix (if applicable) are required and will appear in PubMed.

***First Name and Middle Initial(s)**	***Last Name**	***Suffix (eg, Jr, III)**	Academic Degrees	Institution	Location (city, state/province, country)	Role or Contribution, eg, chair, principal investigator	Group (if more than 1 Group listed in the byline) and/or Subgroup (eg, Steering Committee)
Celeste	Waguespack			Pennington Biomedical Research Center	Baton Rouge, LA, USA	Nurse Practitioner	RECOVER-Pediatric
Katherine	Walgamotte			Pennington Biomedical Research Center	Baton Rouge, LA, USA	Clinical Chem/Lab Tech	RECOVER-Pediatric
Meredith	Welch			Pennington Biomedical Research Center	Baton Rouge, LA, USA	Research RN	RECOVER-Pediatric
Aubrey	Windham			Pennington Biomedical Research Center	Baton Rouge, LA, USA	Lead Coordinator	RECOVER-Pediatric
Tiffany	Bell			University of Louisville Research Foundation	Louisville, KY, USA	Research Coordinator	RECOVER-Pediatric
Jackie	Boyd			University of Louisville Research Foundation	Louisville, KY, USA	Research Coordinator	RECOVER-Pediatric
Soham	Dasgupta			University of Louisville Research Foundation	Louisville, KY, USA	Sub-Investigator	RECOVER-Pediatric
Sarah	Deans			University of Louisville Research Foundation	Louisville, KY, USA	Senior Nurse Coordinator	RECOVER-Pediatric
Katie	Harris			University of Louisville Research Foundation	Louisville, KY, USA	Research Coordinator	RECOVER-Pediatric
Molly	Hemmerle			University of Louisville Research Foundation	Louisville, KY, USA	Research Coordinator	RECOVER-Pediatric
Sarah	King			University of Louisville Research Foundation	Louisville, KY, USA	Research Manager	RECOVER-Pediatric
Cameo	McGuire			University of Louisville Research Foundation	Louisville, KY, USA	Research Coordinator	RECOVER-Pediatric
Brie	Merten			University of Louisville Research Foundation	Louisville, KY, USA	Research Coordinator	RECOVER-Pediatric
Sarah	Morris			University of Louisville Research Foundation	Louisville, KY, USA	Research Coordinator	RECOVER-Pediatric
Madison	Ray			University of Louisville Research Foundation	Louisville, KY, USA	Research Coordinator	RECOVER-Pediatric

*First name, last name, and suffix (if applicable) are required and will appear in PubMed.

***First Name and Middle Initial(s)**	***Last Name**	***Suffix (eg, Jr, III)**	Academic Degrees	Institution	Location (city, state/province, country)	Role or Contribution, eg, chair, principal investigator	Group (if more than 1 Group listed in the byline) and/or Subgroup (eg, Steering Committee)
Brooklyn	Reinhardt			University of Louisville Research Foundation	Louisville, KY, USA	Lead Coordinator	RECOVER-Pediatric
Shellese	Shemwell			University of Louisville Research Foundation	Louisville, KY, USA	Reg. Coordinator	RECOVER-Pediatric
Theresa	Simeon			University of Louisville Research Foundation	Louisville, KY, USA	Senior Nurse Coordinator	RECOVER-Pediatric
Katherine	Walker			University of Louisville Research Foundation	Louisville, KY, USA	RT/Coordinator	RECOVER-Pediatric
Sara	Watson			University of Louisville Research Foundation	Louisville, KY, USA	Medical Director, NCRI	RECOVER-Pediatric
Kathryn	Weakley			University of Louisville Research Foundation	Louisville, KY, USA	Co-investigator	RECOVER-Pediatric
Johnathon	Figliomeni			University of Nebraska Medical Center and Children's Nebraska	Omaha, NE, USA	Coordinator	RECOVER-Pediatric
Laura	Fischer			University of Nebraska Medical Center and Children's Nebraska	Omaha, NE, USA	Coordinator	RECOVER-Pediatric
Denise	Hoover			University of Nebraska Medical Center and Children's Nebraska	Omaha, NE, USA	Coordinator	RECOVER-Pediatric
Megan	Morse			University of Nebraska Medical Center and Children's Nebraska	Omaha, NE, USA	Co-Investigator	RECOVER-Pediatric
Aleisha	Nabower			University of Nebraska Medical Center and Children's Nebraska	Omaha, NE, USA	Co-Investigator	RECOVER-Pediatric
Evan	Roberts			University of Nebraska Medical Center and Children's Nebraska	Omaha, NE, USA	Coordinator	RECOVER-Pediatric
Alice	Sato			University of Nebraska Medical Center and Children's Nebraska	Omaha, NE, USA	Co-Investigator	RECOVER-Pediatric
Joann	Von Bon			University of Nebraska Medical Center and Children's Nebraska	Omaha, NE, USA	Coordinator	RECOVER-Pediatric
David	Archuleta			University of New Mexico Health Sciences Center	Albuquerque, NM, USA	Coordinator	RECOVER-Pediatric

Supplemental Online Content: Nonauthor Collaborators
*First name, last name, and suffix (if applicable) are required and will appear in PubMed.

***First Name and Middle Initial(s)**	***Last Name**	***Suffix (eg, Jr, III)**	Academic Degrees	Institution	Location (city, state/province, country)	Role or Contribution, eg, chair, principal investigator	Group (if more than 1 Group listed in the byline) and/or Subgroup (eg, Steering Committee)
Rebecca	Brito			University of New Mexico Health Sciences Center	Albuquerque, NM, USA	Coordinator	RECOVER-Pediatric
Richard	Campbell			University of New Mexico Health Sciences Center	Albuquerque, NM, USA	C-level supervisor	RECOVER-Pediatric
Jude	Chavez			University of New Mexico Health Sciences Center	Albuquerque, NM, USA	Neuro Administrator	RECOVER-Pediatric
Walter	Dehority			University of New Mexico Health Sciences Center	Albuquerque, NM, USA	Sub-Investigator	RECOVER-Pediatric
Noella	Garcia-Soberanez			University of New Mexico Health Sciences Center	Albuquerque, NM, USA	Coordinator	RECOVER-Pediatric
Eve	Gronert			University of New Mexico Health Sciences Center	Albuquerque, NM, USA	Coordinator	RECOVER-Pediatric
Matthew	Kadish			University of New Mexico Health Sciences Center	Albuquerque, NM, USA	Sub-Investigator	RECOVER-Pediatric
Jerry	Larrabee			University of New Mexico Health Sciences Center	Albuquerque, NM, USA	Sub-Investigator	RECOVER-Pediatric
Debbie	Lovato			University of New Mexico Health Sciences Center	Albuquerque, NM, USA	Coordinator	RECOVER-Pediatric
Karen	Luo			University of New Mexico Health Sciences Center	Albuquerque, NM, USA	Neuro Administrator	RECOVER-Pediatric
Noah	Martinez			University of New Mexico Health Sciences Center	Albuquerque, NM, USA	Coordinator	RECOVER-Pediatric
Analyse	Merlino			University of New Mexico Health Sciences Center	Albuquerque, NM, USA	Coordinator	RECOVER-Pediatric
Emily	Reese			University of New Mexico Health Sciences Center	Albuquerque, NM, USA	Coordinator	RECOVER-Pediatric
Sarah	Ward			University of New Mexico Health Sciences Center	Albuquerque, NM, USA	Neuro Administrator	RECOVER-Pediatric
Kevin	Wilson			University of New Mexico Health Sciences Center	Albuquerque, NM, USA	Neuro Administrator	RECOVER-Pediatric

*First name, last name, and suffix (if applicable) are required and will appear in PubMed.

***First Name and Middle Initial(s)**	***Last Name**	***Suffix (eg, Jr, III)**	Academic Degrees	Institution	Location (city, state/province, country)	Role or Contribution, eg, chair, principal investigator	Group (if more than 1 Group listed in the byline) and/or Subgroup (eg, Steering Committee)
Ryan	Brown			University of Oklahoma Health Sciences Center	Oklahoma City, OK, USA	Sub-Investigator	RECOVER-Pediatric
Ryan	Butchee			University of Oklahoma Health Sciences Center	Oklahoma City, OK, USA	Sub-Investigator	RECOVER-Pediatric
Gina	Fergeson			University of Oklahoma Health Sciences Center	Oklahoma City, OK, USA	RN	RECOVER-Pediatric
Ryan	McKee			University of Oklahoma Health Sciences Center	Oklahoma City, OK, USA	Sub-Investigator	RECOVER-Pediatric
Brandon	Mohler			University of Oklahoma Health Sciences Center	Oklahoma City, OK, USA	Lab Coordinator	RECOVER-Pediatric
Tiffany	Moore			University of Oklahoma Health Sciences Center	Oklahoma City, OK, USA	Lab Coordinator	RECOVER-Pediatric
Valorie	Owens			University of Oklahoma Health Sciences Center	Oklahoma City, OK, USA	Lead Coordinator	RECOVER-Pediatric
Sarah	Stubbs			University of Oklahoma Health Sciences Center	Oklahoma City, OK, USA	Research Coordinator	RECOVER-Pediatric
Timothy	VanWagoner			University of Oklahoma Health Sciences Center	Oklahoma City, OK, USA	Sub-Investigator	RECOVER-Pediatric
Meghan	Bethel			University of Vermont Medical Center	Burlington, VT, USA	Coordinator	RECOVER-Pediatric
Laurie	Chassereau			University of Vermont Medical Center	Burlington, VT, USA	Coordinator	RECOVER-Pediatric
Thomas	Lahiri			University of Vermont Medical Center	Burlington, VT, USA	Sub-Investigator	RECOVER-Pediatric
Lauren	Lake			West Virginia University	Morgantown, WV, USA	Reg. Manager	RECOVER-Pediatric
Kathy	Moffett			West Virginia University	Morgantown, WV, USA	Sub-I	RECOVER-Pediatric
Emily	Polak			West Virginia University	Morgantown, WV, USA	Reg. Assistant	RECOVER-Pediatric
Sarah	Stutler			West Virginia University	Morgantown, WV, USA	Coordinator	RECOVER-Pediatric
Charlotte	Workman			West Virginia University	Morgantown, WV, USA	Coordinator	RECOVER-Pediatric
Emma	Carpenter			Children's Hospital Los Angeles	Los Angeles, CA, USA	CRA	RECOVER-Pediatric

Supplemental Online Content: Nonauthor Collaborators
*First name, last name, and suffix (if applicable) are required and will appear in PubMed.

***First Name and Middle Initial(s)**	***Last Name**	***Suffix (eg, Jr, III)**	Academic Degrees	Institution	Location (city, state/province, country)	Role or Contribution, eg, chair, principal investigator	Group (if more than 1 Group listed in the byline) and/or Subgroup (eg, Steering Committee)
Isabelle	Dhindsa			Children's Hospital Los Angeles	Los Angeles, CA, USA	CRA	RECOVER-Pediatric
Samantha	Mejia			Children's Hospital Los Angeles	Los Angeles, CA, USA	CRC I	RECOVER-Pediatric
Nelly	Moghadam			Children's Hospital Los Angeles	Los Angeles, CA, USA	CRC	RECOVER-Pediatric
Candice	Mulder			Children's Hospital Los Angeles	Los Angeles, CA, USA	Regulatory Supervisor	RECOVER-Pediatric
Sharon	O'Neil			Children's Hospital Los Angeles	Los Angeles, CA, USA	Director of the Neuropsychology Core	RECOVER-Pediatric
Alisha	Osornio			Children's Hospital Los Angeles	Los Angeles, CA, USA	CRC I	RECOVER-Pediatric
Adrian	Rios			Children's Hospital Los Angeles	Los Angeles, CA, USA	CRC I	RECOVER-Pediatric
Sydney	Rosen			Children's Hospital Los Angeles	Los Angeles, CA, USA	CRC III	RECOVER-Pediatric
Andrea	Smith			Children's Hospital Los Angeles	Los Angeles, CA, USA	Lead Coordinator	RECOVER-Pediatric
Deeba	Tabibi			Children's Hospital Los Angeles	Los Angeles, CA, USA	CRA	RECOVER-Pediatric
Sharon	Tang			Children's Hospital Los Angeles	Los Angeles, CA, USA	Associate Director CRSO	RECOVER-Pediatric
Ariana	Teame			Children's Hospital Los Angeles	Los Angeles, CA, USA	CRA	RECOVER-Pediatric
Melissa	Stockwell			Columbia University Irving Medical Center	New York, NY, USA	Principal Investigator	RECOVER-Pediatric
Brett	Anderson			Columbia University Irving Medical Center	New York, NY, USA	Co-Investigator	RECOVER-Pediatric
Tawanda	Aquino			Columbia University Irving Medical Center	New York, NY, USA	Hub/Site Coordinator	RECOVER-Pediatric
Elizabeth	Berg			Columbia University Irving Medical Center	New York, NY, USA	Co-Investigator	RECOVER-Pediatric
Steve	Caddle			Columbia University Irving Medical Center	New York, NY, USA	Co-Investigator	RECOVER-Pediatric
Marina	Catallozzi			Columbia University Irving Medical Center	New York, NY, USA	Co-Investigator	RECOVER-Pediatric
Wendy	Chung			Columbia University Irving Medical Center	New York, NY, USA	Co-Investigator	RECOVER-Pediatric

*First name, last name, and suffix (if applicable) are required and will appear in PubMed.

*First Name and Middle Initial(s)	*Last Name	*Suffix (eg, Jr, III)	Academic Degrees	Institution	Location (city, state/province, country)	Role or Contribution, eg, chair, principal investigator	Group (if more than 1 Group listed in the byline) and/or Subgroup (eg, Steering Committee)
Tom	Connors			Columbia University Irving Medical Center	New York, NY, USA	Co-Investigator	RECOVER-Pediatric
Aliva	De			Columbia University Irving Medical Center	New York, NY, USA	Co-Investigator	RECOVER-Pediatric
Anny	Diaz Perez			Columbia University Irving Medical Center	New York, NY, USA	Research Assistant	RECOVER-Pediatric
Michael	DiLorenzo			Columbia University Irving Medical Center	New York, NY, USA	Co-Investigator	RECOVER-Pediatric
Dani	Dumitriu			Columbia University Irving Medical Center	New York, NY, USA	Co-Investigator	RECOVER-Pediatric
Kanwal	Farooqi			Columbia University Irving Medical Center	New York, NY, USA	Co-Investigator	RECOVER-Pediatric
Michael	Fremed			Columbia University Irving Medical Center	New York, NY, USA	Co-Investigator	RECOVER-Pediatric
Sylvie	Goldman			Columbia University Irving Medical Center	New York, NY, USA	Co-Investigator	RECOVER-Pediatric
Kayla	Kaplan			Columbia University Irving Medical Center	New York, NY, USA	Hub/Site Coordinator	RECOVER-Pediatric
Usha	Krishnan			Columbia University Irving Medical Center	New York, NY, USA	Co-Investigator	RECOVER-Pediatric
Aimee	Layton			Columbia University Irving Medical Center	New York, NY, USA	Co-Investigator	RECOVER-Pediatric
Angela	Lignelli-Dipple			Columbia University Irving Medical Center	New York, NY, USA	Co-Investigator	RECOVER-Pediatric
Son	McClaren			Columbia University Irving Medical Center	New York, NY, USA	Co-Investigator	RECOVER-Pediatric
Jonathan	Overdevest			Columbia University Irving Medical Center	New York, NY, USA	Co-Investigator	RECOVER-Pediatric
Michelle	Rodriguez			Columbia University Irving Medical Center	New York, NY, USA	Coordinator	RECOVER-Pediatric

Supplemental Online Content: Nonauthor Collaborators
*First name, last name, and suffix (if applicable) are required and will appear in PubMed.

*First Name and Middle Initial(s)	*Last Name	*Suffix (eg, Jr, III)	Academic Degrees	Institution	Location (city, state/province, country)	Role or Contribution, eg, chair, principal investigator	Group (if more than 1 Group listed in the byline) and/or Subgroup (eg, Steering Committee)
Jay	Selman			Columbia University Irving Medical Center	New York, NY, USA	Co-Investigator	RECOVER-Pediatric
Wendy	Silver			Columbia University Irving Medical Center	New York, NY, USA	Co-Investigator	RECOVER-Pediatric
Raul	Silverio			Columbia University Irving Medical Center	New York, NY, USA	Coordinator	RECOVER-Pediatric
Ana	Valdez de Romero			Columbia University Irving Medical Center	New York, NY, USA	Research Assistant	RECOVER-Pediatric
Celibell	Vargas			Columbia University Irving Medical Center	New York, NY, USA	Coordinator	RECOVER-Pediatric
Daniella	Caputo			Best Healthcare Inc.	New York, NY, USA	Research Nurse	RECOVER-Pediatric
Camille	Leggieri			Best Healthcare Inc.	New York, NY, USA	Research Nurse	RECOVER-Pediatric
Pamela	Pretsch			Best Healthcare Inc.	New York, NY, USA	Site Coordinator	RECOVER-Pediatric
Kristin	Davis			American Academy of Pediatrics	Itasca, IL, USA	Project Coordinator	RECOVER-Pediatric
Miranda	Griffith			American Academy of Pediatrics	Itasca, IL, USA	Research Assistant	RECOVER-Pediatric
Donna	Harris			American Academy of Pediatrics	Itasca, IL, USA	Research Associate	RECOVER-Pediatric
Everly	Macario			American Academy of Pediatrics	Itasca, IL, USA	Co-Research Coordinator	RECOVER-Pediatric
Jennifer	Steffes			American Academy of Pediatrics	Itasca, IL, USA	Research Assistant	RECOVER-Pediatric
Alessandra	Torres			American Academy of Pediatrics	Itasca, IL, USA	Supervisor	RECOVER-Pediatric
Chris	Day			Childrens Mercy	Kansas City, MO, USA	Co-Investigator	RECOVER-Pediatric
Kelsye	Howell			Childrens Mercy	Kansas City, MO, USA	Research Coordinator	RECOVER-Pediatric
Megan	Mains			Childrens Mercy	Kansas City, MO, USA	Research Coordinator	RECOVER-Pediatric

*First name, last name, and suffix (if applicable) are required and will appear in PubMed.

***First Name and Middle Initial(s)**	***Last Name**	***Suffix (eg, Jr, III)**	Academic Degrees	Institution	Location (city, state/province, country)	Role or Contribution, eg, chair, principal investigator	Group (if more than 1 Group listed in the byline) and/or Subgroup (eg, Steering Committee)
Emily	Bean			Connecticut Children's Medical Center	Hartford, CT, USA	Research Assistant	RECOVER-Pediatric
Carlie	DeFelice			Connecticut Children's Medical Center	Hartford, CT, USA	Research Assistant	RECOVER-Pediatric
Hassan	El Chebib			Connecticut Children's Medical Center	Hartford, CT, USA	Co-I	RECOVER-Pediatric
Katherine W.	Herbst			Connecticut Children's Medical Center	Hartford, CT, USA	Co-I	RECOVER-Pediatric
Stephanie	Lesmes			Connecticut Children's Medical Center	Hartford, CT, USA	Research Assistant	RECOVER-Pediatric
Melissa	Santos			Connecticut Children's Medical Center	Hartford, CT, USA	Co-I	RECOVER-Pediatric
Noah	Schulman			Connecticut Children's Medical Center	Hartford, CT, USA	Coordinator	RECOVER-Pediatric
Alicia	Brooks-Greien			DARTNet Institute/American Academy of Family Physicians, AAFP, NRN,		Research Coordinator	RECOVER-Pediatric
Karinne	Colin			DARTNet Institute/American Academy of Family Physicians, AAFP, NRN,		Research Coordinator	RECOVER-Pediatric
Ariadna	Juarez-Colunga			DARTNet Institute/American Academy of Family Physicians, AAFP, NRN,		Research Coordinator	RECOVER-Pediatric
Brian	Manning			DARTNet Institute/American Academy of Family Physicians, AAFP, NRN,		Coordinator	RECOVER-Pediatric
Joel	Shields			DARTNet Institute/American Academy of Family Physicians, AAFP, NRN,		Research Coordinator	RECOVER-Pediatric

Supplemental Online Content: Nonauthor Collaborators
*First name, last name, and suffix (if applicable) are required and will appear in PubMed.

*First Name and Middle Initial(s)	*Last Name	*Suffix (eg, Jr, III)	Academic Degrees	Institution	Location (city, state/province, country)	Role or Contribution, eg, chair, principal investigator	Group (if more than 1 Group listed in the byline) and/or Subgroup (eg, Steering Committee)
Daphne	York			DARTNet Institute/American Academy of Family Physicians, AAFP, NRN,		Study Coordinator	RECOVER-Pediatric
Justine	Griswold			Hackensack Meridian Health Hospitals Corporation	Hackensack, NJ, USA	Project Manager	RECOVER-Pediatric
Donna	Lee			Hackensack Meridian Health Hospitals Corporation	Hackensack, NJ, USA	Co-Investigator	RECOVER-Pediatric
Amanda	Nowakowski			Hackensack Meridian Health Hospitals Corporation	Hackensack, NJ, USA	Research Nurse	RECOVER-Pediatric
Maryellen	Riordan			Hackensack Meridian Health Hospitals Corporation	Hackensack, NJ, USA	Research Nurse	RECOVER-Pediatric
Rozina	Aamir			MetroHealth System	Cleveland, OH, USA	Research Coordinator	RECOVER-Pediatric
Mohammed	Abuzahrieh			MetroHealth System	Cleveland, OH, USA	Lab assiocate	RECOVER-Pediatric
Nandini	Bangalore			MetroHealth System	Cleveland, OH, USA	Research Coordinator	RECOVER-Pediatric
Alexis	Brown			MetroHealth System	Cleveland, OH, USA	Lab Associate	RECOVER-Pediatric
Wendy	Dalton			MetroHealth System	Cleveland, OH, USA	Lead Study Nurse	RECOVER-Pediatric
Suzanne	Fortuna			MetroHealth System	Cleveland, OH, USA	Research Nurse	RECOVER-Pediatric
Judi	Minium			MetroHealth System	Cleveland, OH, USA	Lab manager	RECOVER-Pediatric
Bonnie	Rosolowski			MetroHealth System	Cleveland, OH, USA	Coordinator	RECOVER-Pediatric
Amal	Ahmed			New York Medical College, Westchester Medical Center	Valhalla, NY, USA	Research Coordinator	RECOVER-Pediatric
Suzanne	Braniecki			New York Medical College, Westchester Medical Center	Valhalla, NY, USA	Co-I	RECOVER-Pediatric
Montserrat	Contreras			New York Medical College, Westchester Medical Center	Valhalla, NY, USA	Research Coordinator	RECOVER-Pediatric
Supriya	Jain			New York Medical College, Westchester Medical Center	Valhalla, NY, USA	Co-I	RECOVER-Pediatric

Supplemental Online Content: Nonauthor Collaborators
*First name, last name, and suffix (if applicable) are required and will appear in PubMed.

*First Name and Middle Initial(s)	*Last Name	*Suffix (eg, Jr, III)	Academic Degrees	Institution	Location (city, state/province, country)	Role or Contribution, eg, chair, principal investigator	Group (if more than 1 Group listed in the byline) and/or Subgroup (eg, Steering Committee)
Suzanne	Kaseta			New York Medical College, Westchester Medical Center	Valhalla, NY, USA	Co-I	RECOVER-Pediatric
Zachary	Messer			New York Medical College, Westchester Medical Center	Valhalla, NY, USA	Research Manager	RECOVER-Pediatric
Armando	Ramirez			New York Medical College, Westchester Medical Center	Valhalla, NY, USA	Research Coordinator	RECOVER-Pediatric
Aalok	Singh			New York Medical College, Westchester Medical Center	Valhalla, NY, USA	Co-I	RECOVER-Pediatric
Randy	WIlliams			New York Medical College, Westchester Medical Center	Valhalla, NY, USA	Research Coordinator	RECOVER-Pediatric
Almary	Akerlundh			Rady Children's/UCSD	San Diego, CA, USA	Research Coordinator	RECOVER-Pediatric
Natacha	Akshoomoff			Rady Children's/UCSD	San Diego, CA, USA	Co-Investigator	RECOVER-Pediatric
Maria	Arroyo			Rady Children's/UCSD	San Diego, CA, USA	Research Coordinator	RECOVER-Pediatric
Wendy	Barrientos			Rady Children's/UCSD	San Diego, CA, USA	Community Partners	RECOVER-Pediatric
Rakesh	Bhattacharjee			Rady Children's/UCSD	San Diego, CA, USA	Co-Investigator	RECOVER-Pediatric
Bryant Y.	Chao			Rady Children's/UCSD	San Diego, CA, USA	Study Admin	RECOVER-Pediatric
Maricela	Diaz			Rady Children's/UCSD	San Diego, CA, USA	Community Partners	RECOVER-Pediatric
Sergio	Garcia			Rady Children's/UCSD	San Diego, CA, USA	Research Coordinator	RECOVER-Pediatric
Sonia	Garcia			Rady Children's/UCSD	San Diego, CA, USA	Research Coordinator	RECOVER-Pediatric
Guadalupe	Gomez			Rady Children's/UCSD	San Diego, CA, USA	Community Partners	RECOVER-Pediatric
Trinidad	Herrera			Rady Children's/UCSD	San Diego, CA, USA	Community Partners	RECOVER-Pediatric
Margarita	Holguin			Rady Children's/UCSD	San Diego, CA, USA	Community Leader	RECOVER-Pediatric

Supplemental Online Content: Nonauthor Collaborators
*First name, last name, and suffix (if applicable) are required and will appear in PubMed.

***First Name and Middle Initial(s)**	***Last Name**	***Suffix (eg, Jr, III)**	Academic Degrees	Institution	Location (city, state/province, country)	Role or Contribution, eg, chair, principal investigator	Group (if more than 1 Group listed in the byline) and/or Subgroup (eg, Steering Committee)
Manaswitha	Khare			Rady Children's/UCSD	San Diego, CA, USA	Co-Investigator	RECOVER-Pediatric
Elizabeth	Kiernan			Rady Children's/UCSD	San Diego, CA, USA	Research Coordinator	RECOVER-Pediatric
Jeremy	Landeo Gutierrez			Rady Children's/UCSD	San Diego, CA, USA	Co-Investigator	RECOVER-Pediatric
Ileana	Matta			Rady Children's/UCSD	San Diego, CA, USA	Research Assistant	RECOVER-Pediatric
Sofia	Reyes			Rady Children's/UCSD	San Diego, CA, USA	Community Partners	RECOVER-Pediatric
Julie	Ryu			Rady Children's/UCSD	San Diego, CA, USA	Co-Investigator	RECOVER-Pediatric
Cinthia	Sanchez			Rady Children's/UCSD	San Diego, CA, USA	Research Coordinator	RECOVER-Pediatric
Andrea	Schreck			Rady Children's/UCSD	San Diego, CA, USA	Research Coordinator	RECOVER-Pediatric
Megan R.	Warner			Rady Children's/UCSD	San Diego, CA, USA	Sr. Research Coordinator	RECOVER-Pediatric
Lisa	Cerracchio			Rutgers Robert Wood Johnson Medical School	New Brunswick, NJ, USA	Nurse Manager	RECOVER-Pediatric
Amber	Folnagy			Rutgers Robert Wood Johnson Medical School	New Brunswick, NJ, USA	Hub Coordinator	RECOVER-Pediatric
Sherri	Gzemski			Rutgers Robert Wood Johnson Medical School	New Brunswick, NJ, USA	Financial Coordinator	RECOVER-Pediatric
Yue	Hao			Rutgers Robert Wood Johnson Medical School	New Brunswick, NJ, USA	Reg. Coordinator	RECOVER-Pediatric
Simon	Li			Rutgers Robert Wood Johnson Medical School	New Brunswick, NJ, USA	Co-I	RECOVER-Pediatric
Sandee	Moroso			Rutgers Robert Wood Johnson Medical School	New Brunswick, NJ, USA	Temp. Coordinator	RECOVER-Pediatric
Manette	Ness-Cochinwala			Rutgers Robert Wood Johnson Medical School	New Brunswick, NJ, USA	Sub-I	RECOVER-Pediatric

Supplemental Online Content: Nonauthor Collaborators
*First name, last name, and suffix (if applicable) are required and will appear in PubMed.

***First Name and Middle Initial(s)**	***Last Name**	***Suffix (eg, Jr, III)**	Academic Degrees	Institution	Location (city, state/province, country)	Role or Contribution, eg, chair, principal investigator	Group (if more than 1 Group listed in the byline) and/or Subgroup (eg, Steering Committee)
Akhil	Patel			Rutgers Robert Wood Johnson Medical School	New Brunswick, NJ, USA	Research Assistant	RECOVER-Pediatric
Benjamin	Richlin			Rutgers Robert Wood Johnson Medical School	New Brunswick, NJ, USA	Research Coordinator	RECOVER-Pediatric
Harsh	Sharma			Rutgers Robert Wood Johnson Medical School	New Brunswick, NJ, USA	Research Assistant	RECOVER-Pediatric
Damaris	Soto			Rutgers Robert Wood Johnson Medical School	New Brunswick, NJ, USA	Study Assistant	RECOVER-Pediatric
Christian	Suarez			Rutgers Robert Wood Johnson Medical School	New Brunswick, NJ, USA	Research Coordinator	RECOVER-Pediatric
Bibiana	Vargas Forero			Rutgers Robert Wood Johnson Medical School	New Brunswick, NJ, USA	Research Coordinator	RECOVER-Pediatric
Elizabeth	Ricciardi			Saint Barnabas Medical Center, NBI	Newark, NJ, USA	Research Coordinator	RECOVER-Pediatric
Vanessa	Trespalacios			Saint Barnabas Medical Center, NBI	Newark, NJ, USA	Co-I	RECOVER-Pediatric
Matthew	Kluko			Yale School of Medicine	New Haven, CT, USA	Research Coordinator	RECOVER-Pediatric
Kyung E.	Rhee		MD, MSc, MA	University of California San Diego (Tantisira)	La Jolla, CA, USA	Hub/Site MPI	RECOVER-Pediatric
Kelan G.	Tantisira		MD, MPH	University of California San Diego (Tantisira)	La Jolla, CA, USA	Hub/Site PI	RECOVER-Pediatric
Diana	Acosta Valle			University of California San Diego (Tantisira)	La Jolla, CA, USA		RECOVER-Pediatric
Almary	Akerlundh		BS,IMG	University of California San Diego (Tantisira)	La Jolla, CA, USA	Hub/Site Coordinator	RECOVER-Pediatric
Natacha	Akshoomoff		PhD	University of California San Diego (Tantisira)	La Jolla, CA, USA	Hub/Site Co-I	RECOVER-Pediatric
Wendy	Barrientos			University of California San Diego (Tantisira)	La Jolla, CA, USA	Promotora	RECOVER-Pediatric

Supplemental Online Content: Nonauthor Collaborators
*First name, last name, and suffix (if applicable) are required and will appear in PubMed.

*First Name and Middle Initial(s)	*Last Name	*Suffix (eg, Jr, III)	Academic Degrees	Institution	Location (city, state/province, country)	Role or Contribution, eg, chair, principal investigator	Group (if more than 1 Group listed in the byline) and/or Subgroup (eg, Steering Committee)
Rakesh	Bhattacharjee		MD	University of California San Diego (Tantisira)	La Jolla, CA, USA	Hub/Site Co-I	RECOVER-Pediatric
Bryant	Chao		BS	University of California San Diego (Tantisira)	La Jolla, CA, USA	Volunteer	RECOVER-Pediatric
Ashvin	Choudhary			University of California San Diego (Tantisira)	La Jolla, CA, USA		RECOVER-Pediatric
Maricela	Diaz			University of California San Diego (Tantisira)	La Jolla, CA, USA	Promotora	RECOVER-Pediatric
Sonia	Garcia			University of California San Diego (Tantisira)	La Jolla, CA, USA	Research Phlebotomist/Medical Assistant	RECOVER-Pediatric
Sergio	Garcia			University of California San Diego (Tantisira)	La Jolla, CA, USA	Hub/Site Coordinator	RECOVER-Pediatric
Maria	Glenn-Arroyo			University of California San Diego (Tantisira)	La Jolla, CA, USA	Research Phlebotomist/Medical Assistant	RECOVER-Pediatric
Guadalupe	Gomez			University of California San Diego (Tantisira)	La Jolla, CA, USA	Promotora	RECOVER-Pediatric
Trinidad	Herrera			University of California San Diego (Tantisira)	La Jolla, CA, USA	Promotora	RECOVER-Pediatric
Margarita	Holguin			University of California San Diego (Tantisira)	La Jolla, CA, USA	Promotora	RECOVER-Pediatric
Manaswitha	Khare		MD	University of California San Diego (Tantisira)	La Jolla, CA, USA	Hub/Site Co-I	RECOVER-Pediatric
Elizabeth A.	Kiernan		MPH	University of California San Diego (Tantisira)	La Jolla, CA, USA	Hub/Site Coordinator	RECOVER-Pediatric
Jeremy	Landeo-Gutierrez		MD, MPH	University of California San Diego (Tantisira)	La Jolla, CA, USA	Hub/Site Co-I	RECOVER-Pediatric
Anika	Madan		BSc	University of California San Diego (Tantisira)	La Jolla, CA, USA	Hub/Site Coordinator	RECOVER-Pediatric

Supplemental Online Content: Nonauthor Collaborators
*First name, last name, and suffix (if applicable) are required and will appear in PubMed.

***First Name and Middle Initial(s)**	***Last Name**	***Suffix (eg, Jr, III)**	Academic Degrees	Institution	Location (city, state/province, country)	Role or Contribution, eg, chair, principal investigator	Group (if more than 1 Group listed in the byline) and/or Subgroup (eg, Steering Committee)
Katelyn	McConnaughey			University of California San Diego (Tantisira)	La Jolla, CA, USA		RECOVER-Pediatric
Lisa	Ramos Vallejo		BA	University of California San Diego (Tantisira)	La Jolla, CA, USA	Hub/Site Coordinator	RECOVER-Pediatric
Sofia	Reyes			University of California San Diego (Tantisira)	La Jolla, CA, USA	Promotora	RECOVER-Pediatric
Julie	Ryu		MD	University of California San Diego (Tantisira)	La Jolla, CA, USA	Hub/Site Co-I	RECOVER-Pediatric
Cinthia E.	Sanchez		BS	University of California San Diego (Tantisira)	La Jolla, CA, USA	Hub/Site Coordinator	RECOVER-Pediatric
Andrea	Schreck		BS	University of California San Diego (Tantisira)	La Jolla, CA, USA	Hub/Site Coordinator	RECOVER-Pediatric
Maira	Suarez		BS	University of California San Diego (Tantisira)	La Jolla, CA, USA	Hub/Site Coordinator	RECOVER-Pediatric
Megan R.	Warner		MS	University of California San Diego (Tantisira)	La Jolla, CA, USA	Hub/Site Coordinator	RECOVER-Pediatric
Stephanie V.	Caldas		PhD	New York University Grossman School of Medicine	New York, NY	Site Co-I	RECOVER-Pediatric
Thomas Dylan	Castro Ovalle		BS	New York University Grossman School of Medicine	New York, NY	Research Associate	RECOVER-Pediatric
Anthony	Chung		BS	New York University Grossman School of Medicine	New York, NY	Research Associate	RECOVER-Pediatric
Jonathan S	Farkas		MD	New York University Grossman School of Medicine	New York, NY	Site Co-I	RECOVER-Pediatric
Maria	Isidoro-Chino		BA	New York University Grossman School of Medicine	New York, NY	Research Associate	RECOVER-Pediatric
Deniz	Kesebir		MD	New York University Grossman School of Medicine	New York, NY	Site Co-I	RECOVER-Pediatric
Eugenia	Kim		BA	New York University Grossman School of Medicine	New York, NY	Research Associate	RECOVER-Pediatric

Supplemental Online Content: Nonauthor Collaborators
*First name, last name, and suffix (if applicable) are required and will appear in PubMed.

***First Name and Middle Initial(s)**	***Last Name**	***Suffix (eg, Jr, III)**	Academic Degrees	Institution	Location (city, state/province, country)	Role or Contribution, eg, chair, principal investigator	Group (if more than 1 Group listed in the byline) and/or Subgroup (eg, Steering Committee)
Ashley	Quarless		BA	New York University Grossman School of Medicine	New York, NY	Research Volunteer	RECOVER-Pediatric
Alan	Schlechter		MD, MS	New York University Grossman School of Medicine	New York, NY	Site Co-I	RECOVER-Pediatric
Ranjini	Srinivasan		MD	New York University Grossman School of Medicine	New York, NY	Site Co-I	RECOVER-Pediatric
Fatoumata	Barry			Rhode Island Hospital	Providence, RI, USA	Site Coordinator	RECOVER-Pediatric
Phoebe	Burton			Rhode Island Hospital	Providence, RI, USA	Site Coordinator	RECOVER-Pediatric
Rosa	Cano Lorente			Rhode Island Hospital	Providence, RI, USA	Research Assistant	RECOVER-Pediatric
Caroline	Cummins			Rhode Island Hospital	Providence, RI, USA	Research Assistant	RECOVER-Pediatric
Stephanie	Wehbe			Rhode Island Hospital	Providence, RI, USA	Research Assistant	RECOVER-Pediatric
Jocelyn	Espinoza			Virginia Commonwealth University	Richmond, VA, USA	Research Coordinator	RECOVER-Pediatric
Sara	Moyer			Virginia Commonwealth University	Richmond, VA, USA	Licensed Clinical Research Coordinator	RECOVER-Pediatric
Amy	Rider			Virginia Commonwealth University	Richmond, VA, USA	Licensed Clinical Research Coordinator	RECOVER-Pediatric
Sally	Russell			Virginia Commonwealth University	Richmond, VA, USA	Data Manager	RECOVER-Pediatric
Michael	Schecter			Virginia Commonwealth University	Richmond, VA, USA	Clinical consultant	RECOVER-Pediatric
Lindsey	Stevenson			Virginia Commonwealth University	Richmond, VA, USA	Licensed Clinical Research Coordinator	RECOVER-Pediatric
Jeffrey	Burns		MD, MPH	Boston Children's Hospital	Boston, MA, USA	Co-Chair	Observational Consortium Steering Committee (OCSC)

Supplemental Online Content: Nonauthor Collaborators
*First name, last name, and suffix (if applicable) are required and will appear in PubMed.

***First Name and Middle Initial(s)**	***Last Name**	***Suffix (eg, Jr, III)**	Academic Degrees	Institution	Location (city, state/province, country)	Role or Contribution, eg, chair, principal investigator	Group (if more than 1 Group listed in the byline) and/or Subgroup (eg, Steering Committee)
Serena	Spudich		MD, MA	Yale School of Medicine	New Haven, CT, USA	Co-Chair	Observational Consortium Steering Committee (OCSC)
Charles	Bailey		MD, PhD	Children's Hospital of Philadelphia	Philadelphia, PA, USA	Convening Chair of the EHR Studies Coordinating Committee	Observational Consortium Steering Committee (OCSC)
Mine	Cicek		PhD	Mayo Clinic	Rochester, MN, USA	Principal Investigator, Biorepository Core	Observational Consortium Steering Committee (OCSC)
Melissa M.	Cortez		DO	University of Utah	Salt Lake City, UT, USA	Subject Matter Expert	Observational Consortium Steering Committee (OCSC)
Felicia	Davis Blakley				USA	Patient, Caregiver and Community Representative	Observational Consortium Steering Committee (OCSC)
David	Goff		MD, PhD		USA	Senior Scientific Program Director From the National Institutes of Health (NIH)	Observational Consortium Steering Committee (OCSC)
Jessica	Lasky-Su		DSc, MS		USA	Subject Matter Expert	Observational Consortium Steering Committee (OCSC)
Lisa T.	Newman		MSPH	RTI International	, MD, USA	Principal Investigator, Administrative Coordinating Center	Observational Consortium Steering Committee (OCSC)

Supplemental Online Content: Nonauthor Collaborators
*First name, last name, and suffix (if applicable) are required and will appear in PubMed.

***First Name and Middle Initial(s)**	***Last Name**	***Suffix (eg, Jr, III)**	Academic Degrees	Institution	Location (city, state/province, country)	Role or Contribution, eg, chair, principal investigator	Group (if more than 1 Group listed in the byline) and/or Subgroup (eg, Steering Committee)
Igho	Ofotokun		MD	Emory University	Atlanta, GA, USA	Convening Chair of the Adult Cohort Coordinating Committee	Observational Consortium Steering Committee (OCSC)
Sudha	Seshadri		MD, DM		USA	Subject Matter Expert	Observational Consortium Steering Committee (OCSC)
James	Stone		MD, PhD	Massachusetts General Hospital, Harvard Medical School	Boston, MA, USA	Convening Chair of the Autopsy Cohort Coordinating Committee	Observational Consortium Steering Committee (OCSC)
Brittany D.	Taylor		MPH		USA	Patient, Caregiver and Community Representative	Observational Consortium Steering Committee (OCSC)
PJ	Utz		MD	Stanford University School of Medicine	Stanford, CA, USA	Subject Matter Expert	Observational Consortium Steering Committee (OCSC)
Neely A.	Williams		MDiv, EdD		USA	Patient, Caregiver and Community Representative	Observational Consortium Steering Committee (OCSC)
Lisa T.	Newman			RTI International	MD, USA	Principal Investigator/Project Director	Administrative Coordinating Center
Julie	Abella		MA, PMP	RTI International	NC, USA	Oversight & Monitoring Lead, Project Manager	Administrative Coordinating Center
Quinn	Barnette			RTI International	NC, USA	COG and BAC Committee Coordinator	Administrative Coordinating Center

Supplemental Online Content: Nonauthor Collaborators
*First name, last name, and suffix (if applicable) are required and will appear in PubMed.

***First Name and Middle Initial(s)**	***Last Name**	***Suffix (eg, Jr, III)**	Academic Degrees	Institution	Location (city, state/province, country)	Role or Contribution, eg, chair, principal investigator	Group (if more than 1 Group listed in the byline) and/or Subgroup (eg, Steering Committee)
Christine	Bevc		PhD	RTI International	FL, USA	Application Review Lead, R3 Seminar Moderator	Administrative Coordinating Center
Jennifer	Beverly		BS, BA	RTI International	NC, USA	Autospy CC Facilitator	Administrative Coordinating Center
Patricia	Ceger			RTI International	NC, USA	Interventions Task Force Facilitator	Administrative Coordinating Center
Julie	Croxford		MPH	RTI International	MD, USA	ASOC Facilitator; Systems Biology WG Facilitator	Administrative Coordinating Center
Emily	Cunningham			RTI International	NC, USA	Project Administration Specialist, Investigator Review Payments	Administrative Coordinating Center
Mike	Enger			RTI International	NC, USA	Omics Task Force Coordinator	Administrative Coordinating Center
Katie	Fain			RTI International	NC, USA	Integrative Physiology Task Force Coordinator	Administrative Coordinating Center
Tonya	Farris			RTI International	DC, USA	Governance Committee Support Lead	Administrative Coordinating Center
Sean	Hanlon			RTI International	NC, USA	Informatics Co-Lead and Web Portal Architect	Administrative Coordinating Center
David	Hines			RTI International	NC, USA	Mechanistic Pathways Task Force Facilitator	Administrative Coordinating Center

Supplemental Online Content: Nonauthor Collaborators
*First name, last name, and suffix (if applicable) are required and will appear in PubMed.

***First Name and Middle Initial(s)**	***Last Name**	***Suffix (eg, Jr, III)**	Academic Degrees	Institution	Location (city, state/province, country)	Role or Contribution, eg, chair, principal investigator	Group (if more than 1 Group listed in the byline) and/or Subgroup (eg, Steering Committee)
Vicki	Johnson-Lawrence		PhD	RTI International	NC, USA	Representative Engagement Co-Lead	Administrative Coordinating Center
Kevin	Jordan			RTI International	OR, USA	Mechanistic Pathways Task Force Coordinator	Administrative Coordinating Center
Craig	Lefebvre		PhD	RTI International	AZ, USA	Communications Lead	Administrative Coordinating Center
Beth	Linas			RTI International	DC, USA	Lead Science Communication Expert	Administrative Coordinating Center
Bryan	Luukinen		MSPH	RTI International	NC, USA	Communications, Content Lead	Administrative Coordinating Center
Meisha	Mandal			RTI International	NC, USA	Omics and Integrative Physiology Task Force Facilitator	Administrative Coordinating Center
Nikki J.	McKoy		MBA, MPH	RTI International	GA, USA	Representative Engagement Co-Lead	Administrative Coordinating Center
Susan	Nance			RTI International	NC, USA	Population Science Task Force Coordinator, PIPP Behavioral and Rehabilitation Subcommittee Coordinator	Administrative Coordinating Center

Supplemental Online Content: Nonauthor Collaborators
*First name, last name, and suffix (if applicable) are required and will appear in PubMed.

***First Name and Middle Initial(s)**	***Last Name**	***Suffix (eg, Jr, III)**	Academic Degrees	Institution	Location (city, state/province, country)	Role or Contribution, eg, chair, principal investigator	Group (if more than 1 Group listed in the byline) and/or Subgroup (eg, Steering Committee)
Ashleigh	Oakland			RTI International	NC, USA	Project Administration Specialist, Representative Review Payments	Administrative Coordinating Center
Demian	Pasquarelli		BA	RTI International	NC, USA	Informatics Co-Lead /REDCap Data Collection	Administrative Coordinating Center
Claire	Quiner			RTI International	NC, USA	Commonalities with Other Post-Viral Syndromes Task Force Facilitator & PIPP Biologics Subcommittee Facilitator	Administrative Coordinating Center
Rita	Sembajwe			RTI International	GA, USA	Committee Support Sub-task Lead and PIPP Drug and Rehabilitation Subcommittee Facilitator	Administrative Coordinating Center
Gwendolyn	Shaw			RTI International	NC, USA	Interventions Task Force Coordinator	Administrative Coordinating Center

Supplemental Online Content: Nonauthor Collaborators
*First name, last name, and suffix (if applicable) are required and will appear in PubMed.

***First Name and Middle Initial(s)**	***Last Name**	***Suffix (eg, Jr, III)**	Academic Degrees	Institution	Location (city, state/province, country)	Role or Contribution, eg, chair, principal investigator	Group (if more than 1 Group listed in the byline) and/or Subgroup (eg, Steering Committee)
Vanessa	Thornburg			RTI International	NC, USA	Commonalities with Other Post-Viral Syndromes Task Force Coordinator and PIPP Complementary and Alternative Medicine Subcomittee Facilitator	Administrative Coordinating Center
Kendall	Tosco			RTI International	NC, USA	OSMB Coordinator	Administrative Coordinating Center
Hannah	Wright		MSPH	RTI International	CA, USA	Application Review Co-Lead	Administrative Coordinating Center
Judith S.	Hochman		MD	NYU Grossman School of Medicine	New York, NY, USA	mPI	Clinical Science Core
Leora I.	Horwitz		MD	NYU Grossman School of Medicine	New York, NY, USA	mPI	Clinical Science Core
Stuart D.	Katz		MD	NYU Grossman School of Medicine	New York, NY, USA	mPI	Clinical Science Core
Andrea B.	Troxel		MD	NYU Grossman School of Medicine	New York, NY, USA	mPI	Clinical Science Core
Lenard	Adler			NYU Langone Health	New York, NY, USA	Co-Investigator	Clinical Science Core
Precious	Akinbo			NYU Langone Health	New York, NY, USA	Clinical Research Associate, Research Progam Manager	Clinical Science Core

*First name, last name, and suffix (if applicable) are required and will appear in PubMed.

***First Name and Middle Initial(s)**	***Last Name**	***Suffix (eg, Jr, III)**	Academic Degrees	Institution	Location (city, state/province, country)	Role or Contribution, eg, chair, principal investigator	Group (if more than 1 Group listed in the byline) and/or Subgroup (eg, Steering Committee)
Ramona	Almenana			NYU Langone Health	New York, NY, USA	Assistant Program Director of Comms and OEC Liaison	Clinical Science Core
Ola	Bello			NYU Langone Health	New York, NY, USA	Data Analyst/SAS Programmer	Clinical Science Core
Sultana	Bhuiyan			NYU Langone Health	New York, NY, USA	Clinical Trial Assistant	Clinical Science Core
Nina	Blachman			NYU Langone Health	New York, NY, USA	Co-Investigator	Clinical Science Core
Ryan	Branski			NYU Langone Health	New York, NY, USA	Co-Investigator	Clinical Science Core
Jasmine	Briscoe			NYU Langone Health	New York, NY, USA	Research Coordinator	Clinical Science Core
Shari	Brosnahan			NYU Langone Health	New York, NY, USA	Co-Investigator	Clinical Science Core
Elliott	Bueler			NYU Langone Health	New York, NY, USA	Senior Research Project Manager	Clinical Science Core
Yvette	Burgos			NYU Langone Health	New York, NY, USA	Senior Program Coordinator	Clinical Science Core
Nina	Caplin			NYU Langone Health	New York, NY, USA	Co-Investigator	Clinical Science Core
Domonique N.	Chaplin		MS	NYU Langone Health	New York, NY, USA	Senior Research Project Manager, Publications & External Affairs	Clinical Science Core
Yu	Chen			NYU Langone Health	New York, NY, USA	Co-Investigator	Clinical Science Core
Shen	Cheng			NYU Langone Health	New York, NY, USA	Data Analyst	Clinical Science Core
Peter	Choe			NYU Langone Health	New York, NY, USA	Financial Analyst	Clinical Science Core
Jess	Choi			NYU Langone Health	New York, NY, USA	Project Manager	Clinical Science Core
Alicia	Chung			NYU Langone Health	New York, NY, USA	Co-Investigator	Clinical Science Core
Richard	Church			NYU Langone Health	New York, NY, USA	MCIT Technical Project Manager	Clinical Science Core

Supplemental Online Content: Nonauthor Collaborators
*First name, last name, and suffix (if applicable) are required and will appear in PubMed.

***First Name and Middle Initial(s)**	***Last Name**	***Suffix (eg, Jr, III)**	Academic Degrees	Institution	Location (city, state/province, country)	Role or Contribution, eg, chair, principal investigator	Group (if more than 1 Group listed in the byline) and/or Subgroup (eg, Steering Committee)
Stanley	Cobos			NYU Langone Health	New York, NY, USA	Clinical Research Associate, Research Progam Manager	Clinical Science Core
Nakia	Croft			NYU Langone Health	New York, NY, USA	Clinical Research Associate	Clinical Science Core
Angelique	Cruz Irving			NYU Langone Health	New York, NY, USA	Clinical Research Associate	Clinical Science Core
Phoebe	Del Boccio			NYU Langone Health	New York, NY, USA	Assistan Program Director Autopsy/Peds/Compliance Peds	Clinical Science Core
Iván	Díaz			NYU Langone Health	New York, NY, USA	Co-Investigator	Clinical Science Core
Vishal	Doshi			NYU Langone Health	New York, NY, USA	Co-Investigator	Clinical Science Core
Samantha	Ebel			NYU Langone Health	New York, NY, USA	Director Contracts	Clinical Science Core
Arline	Faustin			New York University Grossman School of Medicine, NYU Tisch Hospital	New York, NY, USA	Co-Investigator	Clinical Science Core
Elias	Febres			NYU Langone Health	New York, NY, USA	Senior Program Coordinator	Clinical Science Core
Jeffrey	Fine			NYU Langone Health	New York, NY, USA	Co-Investigator	Clinical Science Core
Sandra	Fink			NYU Langone Health	New York, NY, USA	Contracts Manager	Clinical Science Core
Jennifer	Frontera			NYU Langone Health	New York, NY, USA	Co-Investigator	Clinical Science Core
Alejandra	Gonzalez-Duarte			NYU Langone Health	New York, NY, USA	Co-Investigator	Clinical Science Core
Sophia	Hill			NYU Langone Health	New York, NY, USA	Project Manager	Clinical Science Core
Shahidul	Islam			NYU Langone Health	New York, NY, USA	Co-Investigator	Clinical Science Core
Stephen	Johnson			NYU Langone Health	New York, NY, USA	Co-Investigator	Clinical Science Core
Neha	Kansal			NYU Langone Health	New York, NY, USA		Clinical Science Core
Rachel	Kenney			NYU Langone Health	New York, NY, USA	Co-Investigator	Clinical Science Core

Supplemental Online Content: Nonauthor Collaborators
*First name, last name, and suffix (if applicable) are required and will appear in PubMed.

*First Name and Middle Initial(s)	*Last Name	*Suffix (eg, Jr, III)	Academic Degrees	Institution	Location (city, state/province, country)	Role or Contribution, eg, chair, principal investigator	Group (if more than 1 Group listed in the byline) and/or Subgroup (eg, Steering Committee)
Deepshikha	Kewlani			New York University Grossman School of Medicine	New York, NY, USA	Research Coordinator	Clinical Science Core
Gregory	Laynor			NYU Langone Health	New York, NY, USA	Co-Investigator	Clinical Science Core
Terry	Leon			NYU Langone Health	New York, NY, USA	Neuropsych Adult neurocognitive eval (for nih toolbox and cognitive testing)	Clinical Science Core
Zoe A.	Lewczak		BS	New York University Grossman School of Medicine	New York, NY, USA	Program Coordinator	Clinical Science Core
Janelle	Linton			NYU Langone Health	New York, NY, USA	Assistant Program Director of CE and CE Liaison	Clinical Science Core
Max	Logan			NYU Langone Health	New York, NY, USA	Senior Program Coordinator	Clinical Science Core
Nadia	Malik			NYU Langone Health	New York, NY, USA	Clinical Research Associate	Clinical Science Core
Lia	Mamistvalova			NYU Langone Health	New York, NY, USA	Research Nurse	Clinical Science Core
Hannah	Mandel			NYU Langone Health	New York, NY, USA	Senior Research Scientist	Clinical Science Core
Gabrielle	Maranga			NYU Langone Health	New York, NY, USA	Assistant Program Director of Adult/Compliance, Adult	Clinical Science Core
Patenne D.	Mathews		MPH	New York University Grossman School of Medicine	New York, NY, USA	Program Coordinator	Clinical Science Core
Aprajita	Mattoo			NYU Langone Health	New York, NY, USA	Co-Investigator	Clinical Science Core
Tony	Mei			NYU Langone Health	New York, NY, USA	Core Data personnel	Clinical Science Core
Alan	Mendelsohn			NYU Langone Health	New York, NY, USA	Co-Investigator	Clinical Science Core

Supplemental Online Content: Nonauthor Collaborators
*First name, last name, and suffix (if applicable) are required and will appear in PubMed.

***First Name and Middle Initial(s)**	***Last Name**	***Suffix (eg, Jr, III)**	Academic Degrees	Institution	Location (city, state/province, country)	Role or Contribution, eg, chair, principal investigator	Group (if more than 1 Group listed in the byline) and/or Subgroup (eg, Steering Committee)
Emmanuelle	Mercier			NYU Langone Health	New York, NY, USA	Contracts Manager	Clinical Science Core
Patricio	Millar Vernetti			NYU Langone Health	New York, NY, USA	Co-Investigator	Clinical Science Core
Marc	Miller			NYU Langone Health	New York, NY, USA	Financial Analyst	Clinical Science Core
Maika	Mitchell			NYU Langone Health	New York, NY, USA	Senior Director	Clinical Science Core
Andre	Moreira			NYU Langone Health	New York, NY, USA	Co-Investigator	Clinical Science Core
Praveen C.	Mudumbi		MD	New York University Grossman School of Medicine	New York, NY, USA	Project Manager	Clinical Science Core
Erica	Nahin			NYU Langone Health	New York, NY, USA	Neuropsych PhD	Clinical Science Core
Nandini	Nair			NYU Langone Health	New York, NY, USA	Co-Investigator	Clinical Science Core
Joseph	Nekulak			NYU Langone Health	New York, NY, USA	MCIT Senior Programmer	Clinical Science Core
Kellie	Owens			NYU Langone Health	New York, NY, USA	Co-Investigator	Clinical Science Core
Brendan	Parent			NYU Langone Health	New York, NY, USA	Co-Investigator	Clinical Science Core
Nandan	Patibandla			NYU Langone Health	New York, NY, USA	MCIT System Administrator	Clinical Science Core
Peter	Petrov			NYU Langone Health	New York, NY, USA	Senior Financial Analyst	Clinical Science Core
Radu	Postelnicu			NYU Langone Health	New York, NY, USA	Co-Investigator	Clinical Science Core
Isabelle	Randall			NYU Langone Health	New York, NY, USA	Clinical Trial Assistant	Clinical Science Core
Priyatha	Rao			NYU Langone Health	New York, NY, USA	Senior Contracts Specialist	Clinical Science Core
Amy	Rapkiewicz			NYU Langone Health	New York, NY, USA	Co-Investigator	Clinical Science Core
JohnRoss	Rizzo			NYU Langone Health	New York, NY, USA	Co-Investigator	Clinical Science Core
Johana	Rosas			NYU Langone Health	New York, NY, USA	Neuropsych PhD	Clinical Science Core
Chelsea	Rose			NYU Langone Health	New York, NY, USA	Program Coordinator	Clinical Science Core
Christina	Saint-Jean			NYU Langone Health	New York, NY, USA	Project Manager	Clinical Science Core

Supplemental Online Content: Nonauthor Collaborators
*First name, last name, and suffix (if applicable) are required and will appear in PubMed.

***First Name and Middle Initial(s)**	***Last Name**	***Suffix (eg, Jr, III)**	Academic Degrees	Institution	Location (city, state/province, country)	Role or Contribution, eg, chair, principal investigator	Group (if more than 1 Group listed in the byline) and/or Subgroup (eg, Steering Committee)
Michelle	Santacatterina			NYU Langone Health	New York, NY, USA	Co-Investigator	Clinical Science Core
Binita	Shah			NYU Langone Health	New York, NY, USA	Co-Investigator	Clinical Science Core
Aasma	Shaukat			NYU Langone Health	New York, NY, USA	Co-Investigator	Clinical Science Core
Naomi	Simon			NYU Langone Health	New York, NY, USA	Co-Investigator	Clinical Science Core
Aylin	Simsir			NYU Langone Health	New York, NY, USA	Co-Investigator	Clinical Science Core
Miranda	Stinson			NYU Langone Health	New York, NY, USA	Program Coordinator	Clinical Science Core
Wenfei	Tang			NYU Langone Health	New York, NY, USA	Senior Financial Analyst	Clinical Science Core
Vasishta	Tatapudi			NYU Langone Health	New York, NY, USA	Co-Investigator	Clinical Science Core
Sujata	Thawani			NYU Langone Health	New York, NY, USA	Co-Investigator	Clinical Science Core
Mary	Thomas			NYU Langone Health	New York, NY, USA	Administrative Manager	Clinical Science Core
Lorna	Thorpe			New York University Grossman School of Medicine	New York, NY, USA	Co-Investigator	Clinical Science Core
MeeLee	Tom			NYU Langone Health	New York, NY, USA		Clinical Science Core
Ethan	Treiha			NYU Langone Health	New York, NY, USA	Research Coordinator	Clinical Science Core
Jennifer	Truong			NYU Langone Health	New York, NY, USA	Senior Project Manager	Clinical Science Core
Mmekom	Udosen			NYU Langone Health	New York, NY, USA		Clinical Science Core
Jessica	Velazquez-Perez			NYU Langone Health	New York, NY, USA	Program Coordinator	Clinical Science Core
Patricio M.	Vernetti			NYU Langone Health	New York, NY, USA		Clinical Science Core
Crystal	Vidal			NYU Langone Health	New York, NY, USA	Senior Research Project Manager	Clinical Science Core
Anand	Viswanathan			NYU Langone Health	New York, NY, USA	Co-Investigator	Clinical Science Core
Crystal	Wong			NYU Langone Health	New York, NY, USA		Clinical Science Core

Supplemental Online Content: Nonauthor Collaborators
*First name, last name, and suffix (if applicable) are required and will appear in PubMed.

***First Name and Middle Initial(s)**	***Last Name**	***Suffix (eg, Jr, III)**	Academic Degrees	Institution	Location (city, state/province, country)	Role or Contribution, eg, chair, principal investigator	Group (if more than 1 Group listed in the byline) and/or Subgroup (eg, Steering Committee)
Marion J.	Wood		MPH, BS	New York University Grossman School of Medicine	New York, NY, USA	Research Coordinator	Clinical Science Core
Shannon W.	Wuller			NYU Langone Health	New York, NY, USA	Project Manager	Clinical Science Core
Chloe	Young			New York University Grossman School of Medicine	New York, NY, USA	Program Coordinator	Clinical Science Core
Jonah	Zaretsky			NYU Langone Health	New York, NY, USA	Co-Investigator	Clinical Science Core
Susanna	Zavlunova			NYU Langone Health	New York, NY, USA	Senior Project Manager Safety Monitoring	Clinical Science Core
Andrea	Foulkes		ScD	Massachusetts General Hospital	Boston, MA, USA	Principal Investigator	Data Resource Core
Shawn	Murphy		MD, PhD	Massachusetts General Hospital	Boston, MA, USA	Principal Investigator	Data Resource Core
Shreya	Ahirwar			Massachusetts General Hospital	Boston, MA, USA	Biostats	Data Resource Core
Layne L.	Ainsworth			Brigham and Women's Hospital	Boston, MA, USA	Project Manager	Data Resource Core
Sonya	Ajani			Massachusetts General Hospital	Boston, MA, USA	Biostats	Data Resource Core
Ahmad	Alsheikh			Massachusetts General Hospital	Boston, MA, USA	Biostats	Data Resource Core
Rachel	Atchley-Challenner		PhD	Massachusetts General Hospital	Boston, MA, USA	Biostats	Data Resource Core
Paul	Avilach			Harvard Medical School	Boston, MA, USA		Data Resource Core
Trisha T.	Balan			Massachusetts General Hospital	Boston, MA, USA	Biostats	Data Resource Core
Nicholas	Benik			Massachusetts General Hospital	Boston, MA, USA	Data Portals	Data Resource Core
Barbara	Benoit			Massachusetts General Hospital	Boston, MA, USA	Data Portals	Data Resource Core

Supplemental Online Content: Nonauthor Collaborators
*First name, last name, and suffix (if applicable) are required and will appear in PubMed.

***First Name and Middle Initial(s)**	***Last Name**	***Suffix (eg, Jr, III)**	Academic Degrees	Institution	Location (city, state/province, country)	Role or Contribution, eg, chair, principal investigator	Group (if more than 1 Group listed in the byline) and/or Subgroup (eg, Steering Committee)
William J.	Bonaventura			Massachusetts General Hospital	Boston, MA, USA	Biostats	Data Resource Core
Natalie	Boutin			Massachusetts General Hospital	Boston, MA, USA	Leadership	Data Resource Core
Beverly	Brion			Massachusetts General Hospital	Boston, MA, USA	Biostats	Data Resource Core
Andrew	Cagan			Massachusetts General Hospital	Boston, MA, USA	Data Portals	Data Resource Core
Tianrun	Cai			Brigham and Women's Hospital	Boston, MA, USA	Biostats	Data Resource Core
Tingyi	Cao			Massachusetts General Hospital	Boston, MA, USA	Biostats	Data Resource Core
Victor M.	Castro			Massachusetts General Hospital	Boston, MA, USA	Data Portals	Data Resource Core
Xander R.	Cerretani			Brigham and Women's Hospital	Boston, MA, USA	Project Management	Data Resource Core
Mark	Ciriello			Harvard Medical School	Boston, MA, USA	Data Portals	Data Resource Core
Karen	Costenbader		MD, MPH	Brigham and Women's Hospital	Boston, MA, USA	Biostats	Data Resource Core
Dimitar S.	Dimitrov			Massachusetts General Hospital	Boston, MA, USA	Cloud & FISMA	Data Resource Core
Hossein	Estiri		PhD	Massachusetts General Hospital	Boston, MA, USA	Data Portals	Data Resource Core
Meng	Fang			Brigham and Women's Hospital	Boston, MA, USA	Biostats	Data Resource Core
Maria	Fayad			Massachusetts General Hospital	Boston, MA, USA	Biostats	Data Resource Core
Candace H.	Feldman		MD, ScD	Brigham and Women's Hospital	Boston, MA, USA	Biostats	Data Resource Core
Vivian	Gainer			Massachusetts General Hospital	Boston, MA, USA	Project Manager	Data Resource Core
Bhaswati	Ghosh			Massachusetts General Hospital	Boston, MA, USA	Data Portals	Data Resource Core

Supplemental Online Content: Nonauthor Collaborators
*First name, last name, and suffix (if applicable) are required and will appear in PubMed.

*First Name and Middle Initial(s)	*Last Name	*Suffix (eg, Jr, III)	Academic Degrees	Institution	Location (city, state/province, country)	Role or Contribution, eg, chair, principal investigator	Group (if more than 1 Group listed in the byline) and/or Subgroup (eg, Steering Committee)
Randy	Gollub			Massachusetts General Hospital	Boston, MA, USA	Data Portals	Data Resource Core
Alan	Harris			Harvard Medical School	Boston, MA, USA	Data Portals	Data Resource Core
Karl	Helmer			Massachusetts General Hospital	Boston, MA, USA	Data Portals	Data Resource Core
Andrew	Hendrix	III		Harvard Medical School	Boston, MA, USA	Data Portals	Data Resource Core
Ana	Holzbach			Brigham and Women's Hospital	Boston, MA, USA	Data Portals	Data Resource Core
Weixing	Huang			Massachusetts General Hospital	Boston, MA, USA	Biostats	Data Resource Core
Daniel	Kaufman			Massachusetts General Hospital	Boston, MA, USA	Biostats	Data Resource Core
Diane	Keogh			Harvard Medical School	Boston, MA, USA	Data Portals	Data Resource Core
James D.	Kerr			Brigham and Women's Hospital	Boston, MA, USA	Project Management	Data Resource Core
Jeffrey G.	Klann			Massachusetts General Hospital	Boston, MA, USA	Data Portals	Data Resource Core
Jessica A.	Lasky-Su		ScD	Brigham and Women's Hospital	Boston, MA, USA	Biostats	Data Resource Core
Katherine P.	Liao		MD, MPH	Brigham and Women's Hospital	Boston, MA, USA	Biostats	Data Resource Core
Doug	MacFadden			Harvard Medical School	Boston, MA, USA	Data Portals	Data Resource Core
Anupama	Maram			Harvard Medical School	Boston, MA, USA	Data Portals	Data Resource Core
Megan W.	Martel			Massachusetts General Hospital	Boston, MA, USA	Biostats	Data Resource Core
Michael	Mendis			Massachusetts General Hospital	Boston, MA, USA	Data Portals	Data Resource Core
Reeta	Metta			Massachusetts General Hospital	Boston, MA, USA	Data Portals	Data Resource Core
Jonathan	Monteiro			Massachusetts General Hospital	Boston, MA, USA	Biostats	Data Resource Core
Eduardo	Morales			Massachusetts General Hospital	Boston, MA, USA	Data Portals	Data Resource Core

Supplemental Online Content: Nonauthor Collaborators
*First name, last name, and suffix (if applicable) are required and will appear in PubMed.

***First Name and Middle Initial(s)**	***Last Name**	***Suffix (eg, Jr, III)**	Academic Degrees	Institution	Location (city, state/province, country)	Role or Contribution, eg, chair, principal investigator	Group (if more than 1 Group listed in the byline) and/or Subgroup (eg, Steering Committee)
Marc-Danie	Nazaire			Harvard Medical School	Boston, MA, USA	Data Portals	Data Resource Core
Gregory	Neils			Massachusetts General Hospital	Boston, MA, USA	Cloud & FISMA	Data Resource Core
James	Norman			Harvard Medical School	Boston, MA, USA	Cloud & FISMA	Data Resource Core
Henry H.	Paik			Massachusetts General Hospital	Boston, MA, USA	Biostats	Data Resource Core
Heekyong	Park			Massachusetts General Hospital	Boston, MA, USA	Cloud & FISMA	Data Resource Core
Zihan	Qian			Massachusetts General Hospital	Boston, MA, USA	Biostats	Data Resource Core
Kathleen	Rossi-Roh			Massachusetts General Hospital	Boston, MA, USA	Project Management	Data Resource Core
Leah M.	Santacroce		MA	Brigham and Women's Hospital	Boston, MA, USA	Biostats	Data Resource Core
Caitlin A.	Selvaggi			Massachusetts General Hospital	Boston, MA, USA	Biostats	Data Resource Core
William	Simons			Massachusetts General Hospital	Boston, MA, USA	Biostats	Data Resource Core
Lynn A.	Simpson			Massachusetts General Hospital	Boston, MA, USA	Cloud & FISMA	Data Resource Core
Zachary	Strasser			Massachusetts General Hospital	Boston, MA, USA	Data Portals	Data Resource Core
Ayesha	Tariq			Massachusetts General Hospital	Boston, MA, USA	Biostats	Data Resource Core
Madeleine	Thorn			Massachusetts General Hospital	Boston, MA, USA	Biostats	Data Resource Core
Philip	Trewett			Harvard Medical School	Boston, MA, USA	Data Portals	Data Resource Core
Dustin	Van Fleet			Brigham and Women's Hospital	Boston, MA, USA	Project Management	Data Resource Core
Kavishwar B.	Wagholikar			Massachusetts General Hospital	Boston, MA, USA	Data Portals	Data Resource Core

Supplemental Online Content: Nonauthor Collaborators
*First name, last name, and suffix (if applicable) are required and will appear in PubMed.

***First Name and Middle Initial(s)**	***Last Name**	***Suffix (eg, Jr, III)**	Academic Degrees	Institution	Location (city, state/province, country)	Role or Contribution, eg, chair, principal investigator	Group (if more than 1 Group listed in the byline) and/or Subgroup (eg, Steering Committee)
Haoyang	Wang			Massachusetts General Hospital	Boston, MA, USA	Biostats	Data Resource Core
Taowei D.	Wang			Massachusetts General Hospital	Boston, MA, USA	Data Portals	Data Resource Core
Nich	Wattanasin			Massachusetts General Hospital	Boston, MA, USA	Cloud & FISMA	Data Resource Core
Griffin	Weber			Massachusetts General Hospital	Boston, MA, USA	Data Portals	Data Resource Core
Michael A.	Williams			Massachusetts General Hospital	Boston, MA, USA	Data Portals	Data Resource Core
Mine	Cicek		PhD	Mayo Clinic	Rochester, MN, USA	Principal Investigator	PASC Biorepository Core
Thomas J.	Flotte		MD	Mayo Clinic	Rochester, MN, USA	Co-Principal Investigator	PASC Biorepository Core
Erik M.	Boysen			Mayo Clinic	Rochester, MN, USA		PASC Biorepository Core
Nancy	Chang			Mayo Clinic	Rochester, MN, USA		PASC Biorepository Core
Evan	Ellingworth			Mayo Clinic	Rochester, MN, USA	Program Manager	PASC Biorepository Core
Erika	Frisch			Mayo Clinic	Rochester, MN, USA		PASC Biorepository Core
Gary	Welch			Mayo Clinic	Rochester, MN, USA		PASC Biorepository Core
Abdullahi	Yusuf		MBA	Mayo Clinic	Rochester, MN, USA	Quality Specialist	PASC Biorepository Core
Nicole	Zahnle		MEd, MT(ASCP)	Mayo Clinic	Rochester, MN, USA	Education Specialist	PASC Biorepository Core

Supplemental Online Content: Nonauthor Collaborators
*First name, last name, and suffix (if applicable) are required and will appear in PubMed.

***First Name and Middle Initial(s)**	***Last Name**	***Suffix (eg, Jr, III)**	Academic Degrees	Institution	Location (city, state/province, country)	Role or Contribution, eg, chair, principal investigator	Group (if more than 1 Group listed in the byline) and/or Subgroup (eg, Steering Committee)
Marta	Cerda				USA	Co-Chair	National Community Engagement Group
Victor H.	Clash				USA	Co-Chair	National Community Engagement Group
Felicia	Davis Blakley				USA	Co-Chair	National Community Engagement Group
Brittany	Taylor				USA	Co-Chair	National Community Engagement Group
Mike	Zissis				USA	Co-Chair	National Community Engagement Group
Teresa	Akintonwa				USA		National Community Engagement Group
Heather-Elizabeth	Brown				USA		National Community Engagement Group
Debra	Copeland				USA		National Community Engagement Group
Yvonka	Hall				USA		National Community Engagement Group
kevin	kondo				USA		National Community Engagement Group
Lydia	Lerma				USA		National Community Engagement Group
Jacqui	Lindsay				USA		National Community Engagement Group
Heather	Marti				USA		National Community Engagement Group
Christine	Maughan				USA		National Community Engagement Group
Tony	Minor				USA		National Community Engagement Group

Supplemental Online Content: Nonauthor Collaborators
*First name, last name, and suffix (if applicable) are required and will appear in PubMed.

***First Name and Middle Initial(s)**	***Last Name**	***Suffix (eg, Jr, III)**	Academic Degrees	Institution	Location (city, state/province, country)	Role or Contribution, eg, chair, principal investigator	Group (if more than 1 Group listed in the byline) and/or Subgroup (eg, Steering Committee)
Hyatt	Vincent				USA		National Community Engagement Group
Andra L.	Blomkalns		MD, MBA	Stanford University	Stanford, CA, USA	Co-Chair	Presentations and Publications Oversight Committee
Ingrid V.	Bassett		MD, MPH	Massachusetts General Hospital	Boston, MA, USA		Presentations and Publications Oversight Committee
Rebecca G.	Clifton		PhD	The George Washington University	Washington, DC, USA		Presentations and Publications Oversight Committee
Hannah	Davis				USA		Presentations and Publications Oversight Committee
Nathan	Erdmann		MD, PhD	University of Alabama at Birmingham	Birmingham, AL, USA		Presentations and Publications Oversight Committee
Margot	Gage Witvliet		PhD		USA		Presentations and Publications Oversight Committee
Mark P.	Goldberg		MD		USA		Presentations and Publications Oversight Committee
Zoe	Guan			Massachusetts General Hospital	Boston, MA, USA		Presentations and Publications Oversight Committee
Mamta K.	Jain		MD, MPH	UT Southwestern	Dallas, TX, USA		Presentations and Publications Oversight Committee

Supplemental Online Content: Nonauthor Collaborators
*First name, last name, and suffix (if applicable) are required and will appear in PubMed.

***First Name and Middle Initial(s)**	***Last Name**	***Suffix (eg, Jr, III)**	Academic Degrees	Institution	Location (city, state/province, country)	Role or Contribution, eg, chair, principal investigator	Group (if more than 1 Group listed in the byline) and/or Subgroup (eg, Steering Committee)
Jonathan D.	Klein		MD, MPH	Illinois Research Network (ILLInet)	Chicago, IL, USA		Presentations and Publications Oversight Committee
Gregory	Laynor				USA		Presentations and Publications Oversight Committee
Thomas	Martinez				USA		Presentations and Publications Oversight Committee
Rebecca	McGrath				USA		Presentations and Publications Oversight Committee
Sairam	Parthasarathy		MD	The University of Arizona College of Medicine Tucson	Tucson, AZ, USA		Presentations and Publications Oversight Committee
Priscilla	Pemu		MD, MS	Morehouse School of Medicine	Atlanta, GA, USA		Presentations and Publications Oversight Committee
Jacqueline	Rutter				USA		Presentations and Publications Oversight Committee
