# Supplementary material for: Characterizing Long COVID Symptoms During Early Childhood
Source: JAMA Pediatr. 2025 May 27;179(7):781–92. doi: 10.1001/jamapediatrics.2025.1066 (PMC12117493; doi:10.1001/jamapediatrics.2025.1066)
Supplement: Supplement 3. — Data Sharing Statement. [file jamapediatr-e251066-s003.pdf]

Data Sharing Statement

Gross. Characterizing Long COVID Symptoms During Early Childhood. *JAMA Pediatr*. Published May 27, 2025. doi:10.1001/jamapediatrics.2025.1066

Data
Data available: Yes
Data types: Deidentified participant data, Data dictionary
How to access data: Because the RECOVER Pediatric Cohort Observational Study is in progress and the datasets are updated dynamically, individual patient/participant data will not be provided at the time of publication. However, the data dictionary is published on the RECOVER website and a link will be made available. Furthermore, NHLBI has undertaken a significant effort to release harmonized data from all RECOVER observational cohort studies via their BioDataCatalyst platform. When these data are released, the datasets will include participant-level data collected for the present study, as well as the data dictionary, and will be released following approval of a methodologically sound proposal with a signed data access agreement. https://recovercovid.org; https://biodatacatalyst.nhlbi.nih.gov.
When available: With publication

Supporting Documents
Document types: None

Additional Information
Who can access the data: Researchers with a signed access agreement to the BioDataCatalyst platform.
Types of analyses: Data access will be made available for a specified purpose.
Mechanisms of data availability: Data will be released following approval of a methodologically sound proposal with a signed data access agreement.
Any additional restrictions: None.
